# Automatic Forward Model Parameterization with Bayesian Inference of Conformational Populations

**Published:** 2024-05-28

**Authors:** Robert M. Raddi, Tim Marshall, Vincent A. Voelz

**Affiliations:** Department of Chemistry, Temple University, Philadelphia, PA 19122, USA.

## Abstract

To quantify how well theoretical predictions of structural ensembles agree with experimental measurements, we depend on the accuracy of forward models. These models are computational frameworks that generate observable quantities from molecular configurations based on empirical relationships linking specific molecular properties to experimental measurements. Bayesian Inference of Conformational Populations (BICePs) is a reweighting algorithm that reconciles simulated ensembles with ensemble-averaged experimental observations, even when such observations are sparse and/or noisy. This is achieved by sampling the posterior distribution of conformational populations under experimental restraints as well as sampling the posterior distribution of uncertainties due to random and systematic error. In this study, we enhance the algorithm for the refinement of empirical forward model (FM) parameters. We introduce and evaluate two novel methods for optimizing FM parameters. The first method treats FM parameters as nuisance parameters, integrating over them in the full posterior distribution. The second method employs variational minimization of a quantity called the BICePs score that reports the free energy of “turning on” the experimental restraints. This technique, coupled with improved likelihood functions for handling experimental outliers, facilitates force field validation and optimization, as illustrated in recent studies (Raddi et al. 2023, 2024). Using this approach, we refine parameters that modulate the Karplus relation, crucial for accurate predictions of J-coupling constants based on dihedral angles (ϕ) between interacting nuclei. We validate this approach first with a toy model system, and then for human ubiquitin, predicting six sets of Karplus parameters for JHNHα3, JHαC′3, JHNCβ3, JHNC′3, JC′Cβ3, JC′C′3. This approach, which does not rely on any predetermined parameterization, enhances predictive accuracy and can be used for many applications.

## I. INTRODUCTION

In the field of molecular modeling and dynamics, the accuracy of theoretical predictions that reflect real-world observations is crucial. Quantifying the agreement between theory and experiment is highly dependent on the accuracy of forward models—computational frameworks that predict observable quantities from molecular configurations. These models often depend on empirical relationships that link specific molecular properties to experimental measurements.

Model validation and refinement of structural ensembles against NMR observables critically depends on reliable forward models (FMs) that have been robustly parameterized, so that FM error is minimal in the validation/refinement process. An important challenge in the parameterization of FMs is presented by random and systematic errors inherent to the experimental data. These errors need to be considered in the comparison and integration of experimental data with computational models for objective model selection and accurate uncertainty representation.

A further challenge is presented by missing or insufficient examples of known structures than can be used to train forward models. For NMR observables that depend on backbone ϕ-angles, such as J-coupling constants, the reference data from X-ray crystallography may be missing or dynamically averaged, creating large uncertainties in the correct ϕ-angles. Numerous approaches^1–4^ have been developed to address some of these challenges. Some algorithms rely heavily on X-ray crystal structure data; others have many hyperparameters that need to be determined.

To address these challenges, we extend the Bayesian Inference of Conformational Populations (BICePs) algorithm^5,6^ to refine FM parameters. BICePs, a reweighting algorithm, refines structural ensembles against sparse and/or noisy experimental observables, and has been used in many previous applications.^7–10^ BICePs infers all possible sources of error by sampling the posterior distribution of these parameters directly from the data through MCMC sampling BICePs also computes a free energy-like quantity called the BICePs score that can be used for model selection and model parameterization.^6,11,12^

Recently, BICePs was enhanced with a replica-averaging forward model, making it a maximum-entropy (MaxEnt) reweighting method, and unique in that no adjustable regularization parameters are required to balance experimental information with the prior.^6^ With this new approach, the BICePs score becomes a powerful objective function to parameterize optimal models. Here, we show that the BICePs score, which reflects the total evidence for a model, can be used for variational optimization of FM parameters. The BICePs score contains a form of inherent regularization, and has specialized likelihood functions that allow for the automatic detection and down-weighting of the importance of experimental observables subject to systematic error.^6^

To effectively refine FM parameters, we sample over the full posterior distribution of FM parameters. Through this approach, BICePs performs ensemble reweighting and FM parameter refinement simultaneously. Additionally, we show that by variational minimization of the BICePs score, we obtain the same result and show that the two approaches are equivalent, with each method requiring particular considerations. We first demonstrate our method’s effectiveness on a toy model system, and then optimize six distinct sets of Karplus parameters for the human protein ubiquitin, and compare our findings with previously established results. Through this, we aim to showcase a systematic and robust approach to enhancing the accuracy of theoretical predictions, thereby bridging the gap between computational models and experimental observations.

## II. THEORY

*Posterior sampling of forward model parameters gives reliable parameter uncertainties.* BICePs uses a Bayesian statistical framework, inspired by Inferential Structure Determination (ISD)^13^, to model the posterior distribution p(X,σ), for conformational states X, and nuisance parameters σ, which characterize the extent of uncertainty in the experimental observables D:

(1) $$p\left(X,\sigma |D\right)\propto p\left(D|X,\sigma \right)p\left(X\right)p\left(\sigma \right).$$

Here, p(D∣X,σ) is a likelihood function that uses a forward model to enforce the experimental restraints, p(X) is a prior distribution of conformational populations from some theoretical model, and $p(\sigma )∼\sigma^{-1}$ is a non-informative Jeffrey’s prior.

We now consider a specific forward model g(X,θ) with a set of FM parameters θ that we wish to additionally include in the posterior,

(2) p(X,σ,θ∣D)∝p(D∣X,σ,θ)p(X)p(σ)p(θ)

### Replica-averaging.

When BICePs is equipped with a replica-averaged forward model, it becomes a MaxEnt reweighting method in the limit of large numbers of replicas^14–19^. Consider a set of N replicas, $X=\left\{X_{r}\right\}$, where $X_{r}$ is the conformational state being sampled by replica r. To compare the sampled replicas with ensemble-averaged experimental observables, we define a replica-averaged forward model $g(X,\theta )=\frac{1}{N}\sum_{r}^{N}\;g\left(X_{r},\theta \right)$. This quantity is an estimator of the true ensemble-average, with an error due to finite sampling for observable j estimated using standard error of the mean (SEM):14,19 $\sigma_{j}^{SEM}=\sqrt{\frac{1}{N}\sum_{r}^{N}\;{\left(g_{j}\left(X_{r},\theta \right)-\left\langle g_{j}(X,\theta )\right\rangle \right)}^{2}}$. Thus, $\sigma_{j}^{SEM}$ decreases as the square root of the number of replicas.

In the scenario that observables can be collected into different types, e.g., a particular type of vicinal J-coupling, then each collection can be described with its own set of parameters and error distribution. For K distinct sets of FM parameters $\theta =\left\{\theta_{k}\right\}$, the joint posterior distribution for all parameters is

(3) $$p\left(X,\sigma ,\theta |D\right)\propto \prod_{r=1}^{N}p\left(X_{r}\right)\prod_{k=1}^{K}p\left(D_{k}|g\left(X,\theta_{k}\right),\sigma_{k}\right)p\left(\sigma_{k}\right)p\left(\theta_{k}\right)$$

where X is a set of N conformation replicas, and $\theta_{k}$ is the $k^{th}$ set of FM parameters. The $k^{th}$ set has an uncertainty parameter $\sigma_{k}=\sqrt{{\left(\sigma_{k}^{SEM}\right)}^{2}+{\left(\sigma_{k}^{B}\right)}^{2}}$, that describes the total error, arising from both finite sampling ${\left(\sigma_{k}^{SEM}\right)}^{2}$, and uncertainty in the experimental measurements, known as a Bayesian uncertainty parameter $\sigma_{k}^{B}$. The prior distribution of uncertainties $p\left(\sigma_{k}\right)$ is treated as a non-informative Jeffrey’s prior $\left(\sigma_{k}^{-1}\right)$ for each collection of observables, and the posterior of FM parameters p(θ∣D) is recovered by marginalization over all X and σ:

(4) $$p(\theta |D)=\sum_{X}\int p(X,\sigma ,\theta |D)d\sigma$$

### Gradients speed up convergence.

In our methodology, Markov chain Monte Carlo (MCMC) is used to sample the posterior with acceptances following the Metropolis-Hastings (M-H) criterion. Our algorithm can be used with or without gradients. However, significantly faster convergence, especially in higher dimensions, is achieved through an integration of stochastic gradient descent approach. Our gradient descent approach allows for informed updates to the FM parameters, incorporating stochastic noise to facilitate the escape from local minima and enhance exploration of the parameter space.

The update mechanism is succinctly encapsulated in the equation:

(5) $$\theta_{\text{trial}}=\theta_{\text{old}}-l_{\text{rate}}\cdot \nabla u+\eta \cdot 𝒩(0,\;1)$$

where $\theta_{\text{trial}}$ and $\theta_{\text{old}}$ denote the trial parameters and previous parameters, respectively. The learning rate is denoted by $l_{\text{rate}}$, ∇u signifies the computed gradient of BICePs energy function with respect to the parameters θ, and η scales the noise drawn from a standard normal distribution 𝒩(0,1).

This strategic parameter update protocol is designed to satisfy the M-H criterion, ensuring that each step in the parameter space not only moves towards minimizing the energy of the forward model but also adheres to the probabilistic acceptance of potentially non-optimal moves to avoid local optima traps. Ergodic sampling is ensured by "turning off" the gradient after burn-in. The sampling procedure involves: (1) acquiring derivatives of the FM parameters, (2) perturbing these parameters based on the derived information, (3) predict observables using perturbed FM parameters and compute the total energy, and (4) assessing the new energy against the previous to determine acceptance based on the M-H criterion. This ensures a thorough and effective search of the parameter space, leveraging both the landscape topology and stochastic elements to guide the exploration.

### The Good-Bad model accounts for systematic error due to outlier measurements

BICePs now is equipped with sophisticated likelihood models that are extremely robust in the presence of systematic error^6^. Recently, we demonstrated the ability of the Student’s model to account for systematic error for force field optimization^12^. In this work, we use a likelihood function called the Good-Bad model to demonstrate the validity of forward model refinement. The derivatives of the Good-Bad model are far less complicated than the Student’s model.

The Good-Bad likelihood model^6^ assumes that the level of noise is mostly uniform, except for a few erratic measurements. This limits the number of uncertainty parameters that need to be sampled, while still capturing outliers. Consider a model where uncertainties $\sigma_{j}$ for particular observables j are distributed about some typical uncertainty $\sigma^{B}$ according to a conditional probability $p\left(\sigma_{j}|\sigma^{B}\right)$. We derive a posterior for the $k^{th}$ parameter set having a single uncertainty parameter $\sigma^{B}$ by marginalizing over all $\sigma_{j}$

(6) $$p\left(X,\sigma_{0},\theta_{k}|D\right)\propto \prod_{r=1}^{N}p\left(X_{r}\right)\prod_{j=1}^{N_{d}}\int_{\sigma^{SEM}}^{\infty }p\left(d_{j}|g_{j}\left(X,\theta_{k}\right),\sigma_{j}\right)p\left(\sigma_{j}|\sigma_{0}\right)d\sigma_{j}$$

where $\sigma_{0}=\sqrt{{\left(\sigma^{B}\right)}^{2}+{\left(\sigma^{SEM}\right)}^{2}}$. Under the Good-Bad model, we say that the "good" data consists of observables normally distributed about their true values with effective variance $\sigma_{0}^{2}$, while the "bad" data is subject to systematic error, leading to a larger effective variance $\varphi^{2}\sigma_{0}^{2}$, where φ≥1.

By this assignment, $p\left(\sigma_{j}|\sigma_{0}\right)$ from equation 6 becomes

(7) $$p\left(\sigma_{j}|\sigma_{0},\omega ,\varphi \right)=\omega \delta \left(\sigma_{j}-\varphi \sigma_{0}\right)+(1-\omega )\delta \left(\sigma_{j}-\sigma_{0}\right)$$

where 0≤ω<1 describes the fraction of "bad" observables. Since the value of ω is unknown, it is treated as a nuisance parameter, and marginalized over its range. The resulting posterior is

(8) $$\begin{array}{l} p\left(X,\sigma_{0},\varphi ,\theta_{k}|D\right)\propto \prod_{r=1}^{N}\left\{p\left(X_{r}\right)\prod_{j=1}^{N_{d}}\int_{0}^{1}d\omega \int_{\sigma^{\mathrm{SEM}}}^{\infty }\exp\left(-\frac{{\left(d_{j}\;-\;g_{j}\left(X,\;\theta_{k}\right)\right)}^{2}}{2\sigma_{j}^{2}}\right)\frac{\omega \delta \left(\sigma_{j}\;-\;\varphi \sigma_{0}\right)\;+\;(1\;-\;\omega )\delta \left(\sigma_{j}\;-\;\sigma_{0}\right)}{\sqrt{2\pi }\sigma_{j}}d\sigma_{j}\right\}\\ \;=\prod_{r=1}^{N}\left\{p\left(X_{r}\right)\prod_{j=1}^{N_{d}}\left(\frac{\left(1\;-\;H\left(\sigma^{SEM}\;-\;\sigma_{0}\right)\right)}{2\sqrt{2\pi }\sigma_{0}}\exp\left(-\frac{{\left(d_{j}\;-\;g_{j}\left(X,\;\theta_{k}\right)\right)}^{2}}{2\sigma_{0}^{2}}\right)\;+\;\frac{\left(1\;-\;H\left(\sigma^{SEM}\;-\;\varphi \sigma_{0}\right)\right)}{2\varphi \sqrt{2\pi }\sigma_{0}}\exp\left(\;-\;\frac{{\left(d_{j}\;-\;g_{j}\left(X,\;\theta_{k}\right)\right)}^{2}}{2\varphi^{2}\sigma_{0}^{2}}\right)\right)\right\}, \end{array}$$

where H is the Heaviside step function. After marginalization, we are left with the Bayesian uncertainty parameter $\sigma_{0}^{B}$, and an additional parameter φ. Both parameters are sampled in the posterior. When φ=1, the model reverts to a Gaussian likelihood model. When considering the full posterior, this extra nuisance parameter is given a non-informative Jeffrey’s prior, $p(\varphi )∼\varphi^{-1}$.

For a single set of FM parameters (for simplicity), the BICePs energy function, $u=-log\;p\left(X,\sigma_{0},\varphi ,\theta |D\right)$, the negative logarithm of the posterior in its full form is given by

(9) $$u=\sum_{r=1}^{N}-\log\left(p\left(X_{r}\right)\right)-N\sum_{j=1}^{N_{d}}\log\left[\frac{\left(1\;-\;H\left(\sigma^{SEM}\;-\;\sigma_{0}\right)\right)}{2\sqrt{2\pi }\sigma_{0}}\exp\left(\;-\;\frac{{\left(d_{j}\;-\;g_{j}(X,\;\theta )\right)}^{2}}{2\sigma_{0}^{2}}\right)\;+\;\frac{\left(1\;-\;H\left(\sigma^{SEM}\;-\;\varphi \sigma_{0}\right)\right)}{2\varphi \sqrt{2\pi }\sigma_{0}}\exp\left(\;-\;\frac{{\left(d_{j}\;-\;g_{j}(X,\;\theta )\right)}^{2}}{2\varphi^{2}\sigma_{0}^{2}}\right)\right],$$

and when φ=1 our energy function becomes

(10) $$u=\sum_{r=1}^{N}-\log\left(p\left(X_{r}\right)\right)+N\left[\sum_{j=1}^{N_{d}}-\log\left(\frac{1}{\sqrt{2\pi }\sigma_{j}}\right)+\frac{{\left(d_{j}\;-\;g_{j}\left(X,\;\theta \right)\right)}^{2}}{2\sigma_{j}^{2}}-\log\left(p\left(\sigma_{j}\right)\right)\right].$$

The first derivative of equation 9 with respect to the $i^{\text{th}}$ FM parameter $\theta_{i}$ is

(11) $$\frac{\partial u}{\partial \theta_{i}}=N\sum_{j=1}^{N_{d}}\frac{\partial g_{j}(X,\;\theta )}{\partial \theta_{i}}\frac{\left(d_{j}\;-\;g_{j}(X,\;\theta )\right)}{\varphi^{2}\sigma_{0}^{2}}\frac{\left\{\varphi^{3}\left(1\;-\;H\left(\sigma^{SEM}\;-\;\sigma_{0}\right)\right)\exp\left(\frac{{\left(d_{j}-g_{j}(X,\theta )\right)}^{2}}{2\varphi^{2}\sigma_{0}^{2}}\right)\;+\;\left(1\;-\;H\left(-\varphi \sigma_{0}\;+\;\sigma^{SEM}\right)\right)\exp\left(\frac{{\left(d_{j}-g_{j}(X,\theta )\right)}^{2}}{2\sigma_{0}^{2}}\right)\right\}}{\left\{\varphi \left(H\left(\sigma^{SEM}-\sigma_{0}\right)\;-\;1\right)\exp\left(\frac{{\left(d_{j}-g_{j}(X,\theta )\right)}^{2}}{2\varphi^{2}\sigma_{0}^{2}}\right)\;+\;\left(H\left(-\varphi \sigma_{0}\;+\;\sigma^{SEM}\right)\;-\;1\right)\exp\left(\frac{{\left(d_{j}-g_{j}(X,\theta )\right)}^{2}}{2\sigma_{0}^{2}}\right)\right\}},$$

and in the case of φ=1 the gradient becomes

(12) $$\frac{\partial u}{\partial \theta_{i}}=-N\left[\sum_{j=1}^{N_{d}}\frac{\partial g_{j}\left(X,\;\theta \right)}{\partial \theta_{i}}\frac{\left(d_{j}\;-\;g_{j}\left(X,\;\theta \right)\right)}{\sigma_{j}^{2}}\right].$$

Second derivatives of the BICePs energy function and the BICePS score are useful for descent and uncertainty quantification using other forward models. We refrain from writing out the second derivative here, since the specific class of forward models we consider below all have second derivatives that go to zero. For more general cases, see Appendix A for more details. The energy of the Good-Bad likelihood model and its first and second derivatives are shown in Figure S1.

## III. RESULTS/DISCUSSION

### Testing algorithm performance on a toy model

**Figure 1.**
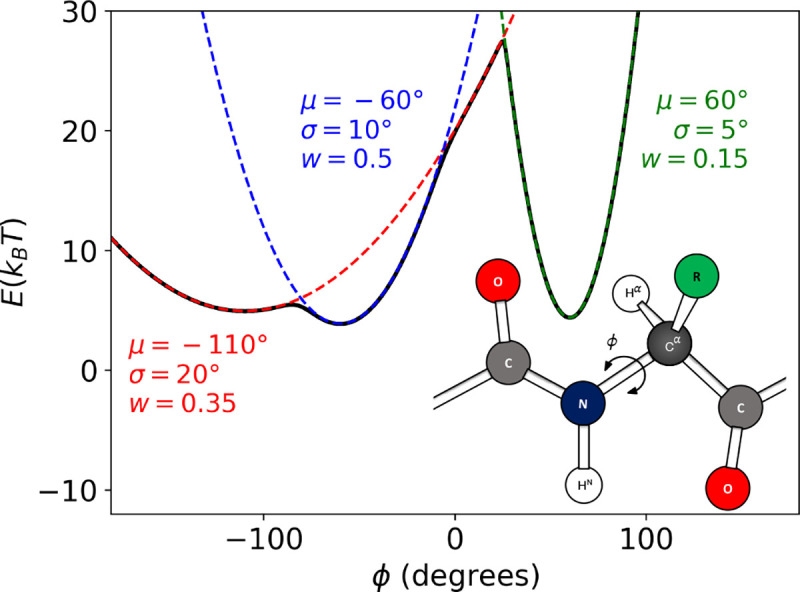
A versatile toy model for measuring the performance of forward model optimization. The ϕ-angles for each conformational state is pulled from a multi-modal distribution and corresponding energies. (a) This multi-modal distribution of ϕ-angles was intended to represent configurations with different secondary structure elements having three distinct modes described by the mean (μ), standard deviation (σ) and weight (w): beta sheets ($\mu =-110^{\circ }$, $\sigma =20^{\circ }$, w=0.35), right-handed helices ($\mu =-60^{\circ }$, $\sigma =10^{\circ }$, w=0.5), and left-handed helices ($\mu =60^{\circ }$, $\sigma =5^{\circ }$, w=0.15). (b) Cartoon representation of the backbone torsion angle, ϕ.

To investigate the efficacy of BICePs for this optimization problem, we introduce a simplified, yet comprehensive toy model. This model is designed to mimic the complexity of protein structure elements by generating ϕ-angles from a multimodal distribution, thereby emulating configurations characteristic of different secondary structure elements (Figure 1). This distribution encompasses three distinct modes, each characterized by a mean (μ), standard deviation (σ), and weight (w): beta sheets ($\mu =-110^{\circ }$, $\sigma =20^{\circ }$, w=0.35), right-handed helices ($\mu =-60^{\circ }$, $\sigma =10^{\circ }$, w=0.5), and left-handed helices ($\mu =60^{\circ }$, $\sigma =5^{\circ }$, w=0.15). These parameters were chosen to accurately reflect the structural variability found in proteins. Angles $\phi_{i}$ were sampled from the multi-modal distribution,

(13) $$p\left(\phi |\mu ,\sigma \right)=\sum_{l}w_{l}\frac{1}{\sqrt{2\pi \sigma_{l}^{2}}}\exp\left(-\frac{{\left(\phi \;-\;\mu_{l}\right)}^{2}}{2\sigma_{l}^{2}}\right).$$

The sampled $\phi_{i}$ were then used to calculate experimental J-coupling constants J(ϕ) using the Karplus relation with the *true* Karplus coefficients $\left(A^{*},B^{*},C^{*}\right)$.


(14) J3ϕ=Acos2ϕ+Bcos(ϕ)+C

Synthetic experimental J-coupling data is generated to represent a mixture of all conformational states, djExp=∑X J3ϕX,j⋅p(X), with FM parameters θ={A,B,C} set to their true values ({A=6.51,B=-1.76,C=1.6}). The initial forward model data is generated using reference Karplus parameters $\left(A_{0},B_{0},C_{0}\right)$ and refined through the optimization process to showcase the algorithm’s adaptability and precision in parameter estimation.

### BICePs robustly finds optimal Karplus parameters in the presence of experimental errors.

To evaluate the resilience of our algorithm against experimental inaccuracies, we introduced random and systematic errors of varying magnitudes ($\sigma_{\text{data}}$) into the synthetic experimental scalar couplings. The performance of our Good-Bad likelihood model, a Gaussian likelihood model, and singular value decomposition (SVD) was compared under these conditions.

*SVD calculations.* Using methods similar to previous efforts by others,^20^ we derived the Karplus parameters θ={A,B,C} using a weighted singular value decomposition (SVD) fitting approach to optimally fit the J-coupling values as a function of dihedral angles. For each observation j across $N_{d}$ measurements, the matrix M was constructed with rows for each ϕ angle:

(15) $$M=\left[\begin{array}{ccc} \sum_{X}\;p(X){cos}^{2}\left(\phi_{1,X}+\phi_{0}\right) & \sum_{X}\;p(X)cos\left(\phi_{1,X}+\phi_{0}\right) & 1\\ \sum_{X}\;p(X){cos}^{2}\left(\phi_{2,X}+\phi_{0}\right) & \sum_{X}\;p(X)cos\left(\phi_{2,X}+\phi_{0}\right) & 1\\ \vdots & \vdots & \vdots \\ \sum_{X}\;p(X){cos}^{2}\left(\phi_{N_{d},X}+\phi_{0}\right) & \sum_{X}\;p(X)cos\left(\phi_{N_{d},X}+\phi_{0}\right) & 1 \end{array}\right]$$

where p(X) represents the true populations for state X, and $\phi_{0}$ is the phase shift of −60°.

SVD was applied to decompose the matrix as $M=U\Sigma V^{T}$, and Karplus coefficients were derived using:

(16) $$\theta =V^{T}(\Sigma +\varepsilon I{)}^{-1}U^{T}J^{exp},$$

where ε=1e-6 a small regularization term added to the diagonal of Σ to ensure stability of the pseudo-inverse, and $J^{\text{exp}}$ represents the vector of experimental J-coupling values. This method ensures robust estimation of θ under ideal experimental conditions, given the true conformational populations. In practice, the true populations are not known *a priori*. The uncertainty in SVD coefficients was determined through 1k iterations of fitting, each omitting 10% of the data points chosen at random.

Typical uncertainties in NMR frequency measurements range from 0.1 to 1.0 Hz, primarily influenced by magnetic field strength, instrument quality, sample conditions, and the specifics of the pulse sequence used. In these experiments, 100 conformational states and 60 synthetic experimental scalar couplings were used. We introduced systematic error by shifting the experimental J3 values by +2.0 Hz to +4.0 Hz for up to 20% of the data points. BICePs calculations were performed by averaging FM parameters over three chains of MCMC stating from different initial parameters ({A=9,B=-1,C=1}, {A=4,B=0,C=3}, {A=0,B=0,C=0}). Regardless of different starting parameters, posterior sampling universally converges to "true" optimal FM parameters. In these calculations, we used 32 BICePs replicas, and burned 10k steps followed by 50k steps of MCMC sampling.

We evaluated model performance by the root-mean-square error (RMSE) between the true J-coupling values with parameters $\left\{A^{*}=6.51,B^{*}=-1.76,C^{*}=1.6\right\}$ and the J-coupling values using predicted Karplus coefficients for all 60 synthetic measurements, performed over 1k independent trials of random generations of toy model data. Average RMSE results, computed over 100 BICePs calculations, highlight the algorithm’s robustness and its ability to accurately predict FM parameters even in the presence of data perturbations. Error bars in our results represent the standard deviation across these calculations, providing a comprehensive measure of the algorithm’s reliability under various experimental accuracy.

**Figure 2.**
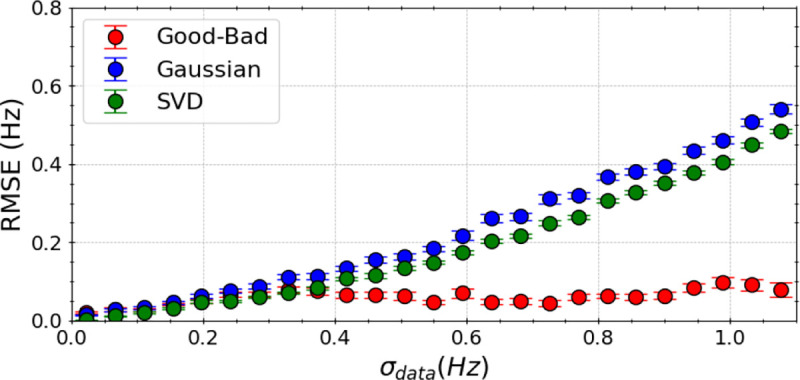
Comparative analysis in performance of the Good-Bad likelihood model (red), a Gaussian likelihood model (blue), and singular value decomposition (SVD) using the "true" ϕ angles with synthetic experimental data. Here, we induced random and systematic error of varying magnitude $\left(\sigma_{\text{data}}\right)$ to the experimental scalar couplings. Model performance was measured by computing RMSE (Hz) between the "true" scalar couplings and the couplings generated from the Karplus relations with predicted Karplus coefficients over 1,500 random perturbations to the experimental data, and represent the average of 100 BICePs calculations. Error bars represent the standard deviation. Predictions from SVD and the Gaussian likelihood model become notably less dependable when data incorporates errors, especially when $\sigma_{\text{data}}$ exceeds 0.5 Hz.

Our findings indicate that the Good-Bad likelihood model (red) exhibits superior resilience to experimental errors compared to a traditional Gaussian likelihood model (blue) and SVD (green) approaches (Figure 2). Predictions from SVD and the Gaussian likelihood model become notably less dependable when data incorporates errors, especially when $\sigma_{\text{data}}$ exceeds 0.5 Hz. On average, error in predictions (RMSE) from the Good-Bad model does not exceed 0.1 Hz over the full range of $\sigma_{\text{data}}$.

An example of a single trial of forward model parameter refinement using the toy model is shown in Figure S2, where BICePs predicts Karplus coefficients by posterior sampling over FM parameters. Both BICePs and Singular Value Decomposition (SVD) methods successfully reproduce the "true" Karplus curve. However, BICePs excels by accurately identifying the error present in the data $\left(\sigma_{\text{data}}=0.471\right)$, as indicated in the marginal posterior of uncertainty $p\left(\sigma_{J}\right)$. The BICePs predicted maximum a posteriori uncertainty was found to be $\sigma_{J}=0.272$ with a variance scaling parameter of $\varphi_{J}=1.98$. The marginal posterior distributions of FM parameters for the Good-Bad model were {A=6.6±0.04,B=-1.8±0.02,C=1.5±0.03}, and for SVD, {A=6.11±0.06,B=-1.63±0.04,C=1.80±0.04}.

In addition to the Good-bad model, we refined parameters using the Student’s model ({A=6.8±0.03,B=-1.9±0.03,C=1.4±0.03}) to demonstrate that the Student’s model yields similar performance (Figure S3). The computed Gelman-Rubin $\left(\overset{ˆ}{R}\right)$ statistic for these calculations was found to be $\overset{ˆ}{R}=1.01$ for each of the marginal posterior distributions of Karplus coefficients, which demonstrates that our chains converge to the same parameter location with similar variance.

Furthermore, we assessed model performance across varying qualities of prior structural ensembles as illustrated in Figure S4. By introducing varying levels of prior error $\sigma_{prior}$ (measured in degrees) through perturbations to the "true" ϕ angles, even in the presence of random and systematic error, we observed strong correlation between the BICePs score and the quality of the structural ensemble, with a coefficient of determination $R^{2}$ of 0.99. For these calculations, we employed the Good-Bad model, utilizing 32 replicas, and conducted 1,000 random perturbations to the ϕ angles with errors up to $\sigma_{prior}=4^{\circ }$, and perturbations to the experimental data $\sigma_{data}=0.68\pm 0.24\;Hz$. Karplus’s warning about the perils of precise angle estimation^21^ underscores our approach’s necessity and performance in error aware modeling in structural biology.

The comprehensive evaluation of our algorithm with this toy model underscores its efficacy in accurately determining FM parameters, reflecting scenarios commonly encountered in real-world applications. The robust performance of the algorithm, even in the face of random and systematic errors, can be attributed to BICePs’ sophisticated error-handling within its likelihood models. This approach also ensures that predicted FM parameters derived from sub-optimal structural ensembles remain reliable. Additionally, our findings reveal a strong correlation between the BICePs score and the quality of the structural ensemble, demonstrating an immense utility in this context.

### Variational minimization of the BICePs score to find optimal parameters.

Treating the forward model parameters as nuisance parameters, and sampling over them with the full posterior is an efficient strategy that grants the ability to include all sources of error while refining the structural ensemble with FM parameters. However, in the limit of large number of FM parameters, the dimensionality of the posterior may ultimately become unwieldy and present the curse of dimensionality. Here, we introduce an alternative strategy for refining FM parameters that has previously demonstrated to be a viable approach to automated force field optimization^12^.

In this approach, the FM parameters are no longer part of the joint posterior density. Instead, the posterior is conditioned on the set of FM parameters θ, that is, equation 2 becomes

(17) p(X,σ∣D,θ)∝p(D,θ∣X,σ)p(X)p(σ)

In this view, ensemble refinement is performed with a static set of FM parameters for each BICePs calculation.

BICePs evaluates model quality by calculating a free energy-like quantity called the BICePs score. For a forward model with parameters θ, the BICePs score f(θ) is computed as the negative logarithm of a Bayes factor comparing the total evidence of a given model against a well-defined reference, marginalizing over all uncertainty,

(18) $$f(\theta )=-ln\left(Z(\theta )/Z_{0}\right),$$

where

(19) Z(θ)=∬exp(-u(X,σ∣D,θ))dXdσ

is the evidence for FM parameters θ, $Z_{0}$ is the evidence for a suitable reference state, and u is the unchanged BICePs energy function (equation 9). To construct the reference state, we consider a series of likelihoods $p_{\xi }(D,\theta |X,\sigma )∼[p(D|X,\sigma ){]}^{\xi }$ parameterized by ξ∈[0,1], and set the reference state as the thermodynamic ensemble corresponding to ξ=0. The BICePs score is then calculated as the change in free energy of "turning on" experimental restraints (ξ=0→1).

It should be noted that in other applications of BICePs,^6,12^ the reference state for the BICePs score is defined using the λ=0 state for a series of a priors $p_{\lambda }{\left(X)∼[p(X)\right]}^{\lambda }$, and the BICePs score is computed as the free energy of (λ=0→1) and (ξ=0→1) transformations. Here, since we are only interested in evaluating and/or parameterizing the likelihood functions, we set p(X) to be uniform. Constructing p(X) is thus very straight-forward: it’s a collection of conformations all having equal statistical weight.

The derivative of the BICePs score with respect to the FM parameters θ reduces to the difference of Boltzmann averaged values of ∂u/∂θ shown as

(20) $$\frac{\partial f(\theta )}{\partial \theta_{i}}=\iint \;\frac{1}{Z(\theta )}\left[\frac{\partial u}{\partial \theta_{i}}\right]exp(-u)dXd\sigma =\langle \frac{\partial u}{\partial \theta_{i}}\rangle$$

In this study, we demonstrate our methodology using first-order optimization methods, such as L-BFGS-B. For more complex forward models, the employment of second derivatives might become necessary. Interested readers are directed to the Supporting Information for second derivatives of the BICePs score with respect to FM parameters.

Calculation of the BICePs score (a free energy difference) and its derivatives (expectation values of energy derivative observables) is performed using the MBAR free energy estimator,^22^ by sampling at several intermediates ξ=0→1, which enables accurate estimates of all quantities.

#### Optimizing ξ-values.

The accuracy of the BICePs score-depends on converged sampling and sufficient thermodynamic overlap of intermediates (ξ=0→1) in the BICePs computation. To ensure strong overlap, we optimize the ξ-values by spacing ensembles equidistantly in thermodynamic length, employing a strategy akin to the "thermodynamic trailblazing" method proposed by Rizzi et al.^23^ Our approach is facilitated by a custom optimization algorithm called <monospace>pylambdaopt</monospace> (Zhang et al., in preparation).

The optimization process is a two-step process: First, a preliminary BICePs calculation is performed using provisional ξ-values, yielding estimates of the thermodynamic length $\left|\ell \left(\xi_{n+1}\right)-\ell \left(\xi_{n}\right)\right|$ for each pair of intermediates^24,25^, derived from the variance in distributions $p\left(\Delta u_{n,n+1}\right)$, where $\Delta u_{n,n+1}=u_{n+1}-u_{n}$ represents the change in the (reduced) BICePs energy incurred by bringing a sample from thermodynamic ensemble n to thermodynamic ensemble n+1.

Second, cubic spline fitting is employed to derive a smooth and differentiable function ℓ(ξ) that accurately interpolates the computed $\ell \left(\xi_{i}\right)$. Optimization through steepest-descent minimization is then applied to determine new $\xi_{i}^{*}$ values that minimize the loss function $ℒ=\sum_{n}\;{\left|\ell \left(\xi_{n+1}\right)-\ell \left(\xi_{n}\right)\right|}^{2}$. This results in $\xi_{i}^{*}$ values uniformly spaced in terms of thermodynamic length, thus maximizing the thermodynamic overlap between adjacent ensembles and enhancing the precision of free energy calculations. These optimized $\xi_{i}^{*}$ values are subsequently used in production runs. An illustration of the ξ-values pre- and post-optimization is depicted in Figure S5. Refer to figures S6&S7 for overlap matrices pre- and post- optimization.

### Comparison of variational minimization of the BICePs score vs. sampling the full joint posterior

In the comparison of the two approaches for parameter estimation and optimization in our model, we utilized a toy model (Figure S2) to evaluate the efficacy of each method under the same data conditions. Prior to FM parameter refinement, 11 ξ-values were optimized from {1.0, 0.9, 0.8, …, 0.0} to {1.0, 0.7, 0.56, 0.45, 0.36, 0.28, 0.2, 0.14, 0.08, 0.04, 0.0} (Figures S5-S7). Variational minimization using the Good-Bad model with 4 replicas (for reduced computational cost), where each evaluation of the objective function consisted of running 10k MCMC steps. Optimal parameters were determined to be {A=6.31±0.02,B=-1.69±0.03,C=1.69±0.01}, averaged over 3 independent runs with very low variance between runs, shown in Figure S8. Regardless of different starting parameters ({A=9,B=-1,C=1}, {A=4,B=0,C=3}, {A=0,B=0,C=0}), variational minimization converges to “true” optimal FM parameters. This analysis demonstrated that both the joint posterior sampling approach and variational minimization yield near equivalent performance when applied to this model.

As a method for forward model optimization, variational minimization of the BICePs score has advantages and disadvantages. This method is particularly advantageous for handling many FM parameters, offering a potential solution to the curse of dimensionality faced by Monte Carlo Markov Chain (MCMC) methods. Additionally, it is easier for users to adapt different forward models, and performs exceptionally well in convex landscapes. When landscapes are non-convex, however, the inverse Hessian may not provide a comprehensive view of the parameter space’s uncertainty; instead, uncertainty estimation could be computed using the variance across multiple BICePs runs starting from different initial parameters. The variational minimization approach also requires careful consideration of disperse starting parameters to ensure global minimization.

The joint posterior sampling method, which involves sampling the joint posterior distribution of forward model (FM) parameters, has several advantages. One significant benefit is that the posterior distribution provides a direct estimate of the uncertainties in forward model parameters and their covariance. Compared to variational minimization, this method generally has a faster runtime and is particularly effective in handling non-convex landscapes, allowing for robust parameter estimation even in complex scenarios. However, it is not without drawbacks. As the number of FM parameters increases, the posterior sampling method may encounter the curse of dimensionality, which makes it computationally challenging to explore the parameter space efficiently.

In summary, while both approaches are valuable tools for parameter estimation in parameter and ensemble refinement, each has its strengths and weaknesses. The choice between these methods should be guided by the specific characteristics of the problem at hand, such as the landscape’s convexity and the number of parameters involved.

### Determination of optimal Karplus coefficients for ubiquitin

To evaluate the performance of our algorithm, we applied BICePs to human ubiquitin to predict Karplus coefficients for six sets of scalar coupling constants: JHNHα3, JHαC′3, JHNCβ3, JHNC′3, JC′Cβ3, and JC′C′3. To test our algorithm’s robustness, we conducted a comprehensive evaluation for predicting optimal Karplus coefficients using three different structural ensembles as priors, each derived from distinct computational approaches: (1) 10 conformations from the NMR-refined structural ensemble, 1D3Z^26^, (2) 144 conformations from NMR-restrained simulations, 2NR2^27^, and (3) 25 conformations from the RosettaFold2 (RF2) algorithm.^28^

We then validated the forward model parameters derived from each prior using the BICePs score, *R*^2^ and mean absolute errors (MAE) for forward model predictions. As priors for these calculations, we used three independent structural ensembles: 1D3Z, 2NR2, and a 500-state conformational ensemble derived from a millisecond-long simulation of ubiquitin using CHARMM22*.^29^ For further details on these ensembles, refer to the SI methods section.

To refine the forward model (FM) parameters, we employed full joint posterior distribution sampling. This method was chosen to navigate the non-convex parameter space efficiently, given its relatively low dimensionality (18 FM parameters). BICePs calculations were executed by averaging the FM parameters over four Markov Chain Monte Carlo (MCMC) chains, each starting from distinct initial parameters: {A=9,B=-1,C=1}, {A=4,B=0,C=3}, {A=0,B=0,C=0}, and {A=6,B=-1,C=0}. Flexible residues were excluded from the calculations, consistent with previous studies^2,3^. As a result, a total of 346 J-couplings were used in these refinements. We used the Good-Bad model with 32 BICePs replicas, discarding the first 50k steps as burn-in, followed by 50k steps for MCMC sampling. Unlike the parameters derived from 1D3Z and RF2, the Karplus coefficients obtained by using the 2NR2 ensemble required a burn-in of 100k steps to appropriately converge due to a larger number of conformational states. The six sets of refined Karplus coefficients resulting from the 1D3Z, 2NR2 and RF2 ensembles are presented in Table I.

**Figure 3.**
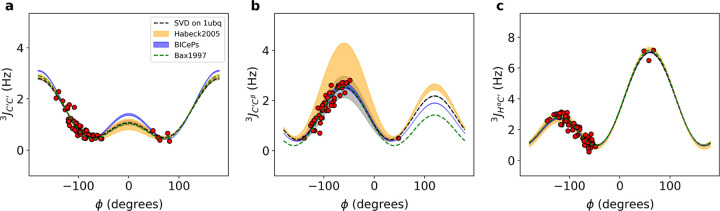
Karplus curves with BICePs-refined Karplus coefficients using the 1d3z ensemble for (a-c) JC′C′3, JC′Cβ3, and JHαC′3. For comparison, SVD on 1ubq using experimental scalar coupling constants with ϕ-angles derived from the X-ray structure (black dashed line), and red dots correspond to the fitted data points. Additionally, parameterizations from Bax et al. 1997 (green) and parameterization from Habeck et al. 2005 (yellow) were overlaid for comparison. The thickness of the line corresponds to the uncertainty.

Figure 3 compares the Karplus curves derived from BICePs using the 1D3Z ensemble with previously published parameters obtained from NMR refinements, showing subtle differences. Both the marginal posterior distributions of the FM parameters and the Karplus curves for each scalar coupling demonstrate significant congruence with the historical NMR refinement results^3,26^. For all six types of J-coupling, see Figure S9.

The predicted parameters, better represented by the marginal posterior distributions of the FM parameters, have large similarities across structural ensembles. BICePs-predicted coefficients using the 1D3Z ensemble (Figure S10) and predicted coefficients using the RF2 ensemble (Figure S11) are found to have very strong overlap. Furthermore, the traces of the FM parameters ober time (Figure S12) confirm convergence.

One advantage of BICePs is that as FM parameters are being sampled, the posterior densities of FM uncertainties, p(σ), are also revealed (Figure S13). For certain sets of J-coupling constants (e.g., JHNHα3 and JHNC′3) the marginal posterior distribution of the variance scaling parameter p(φ) has a sampled mean slightly larger than 1.0, indicating that the functional form of the likelihood opted for long tails to account for a few outlier data points deviating from the mean.

#### The BICePs free energy landscape for JHNC′3 Karplus parameters.

**Figure 4.**
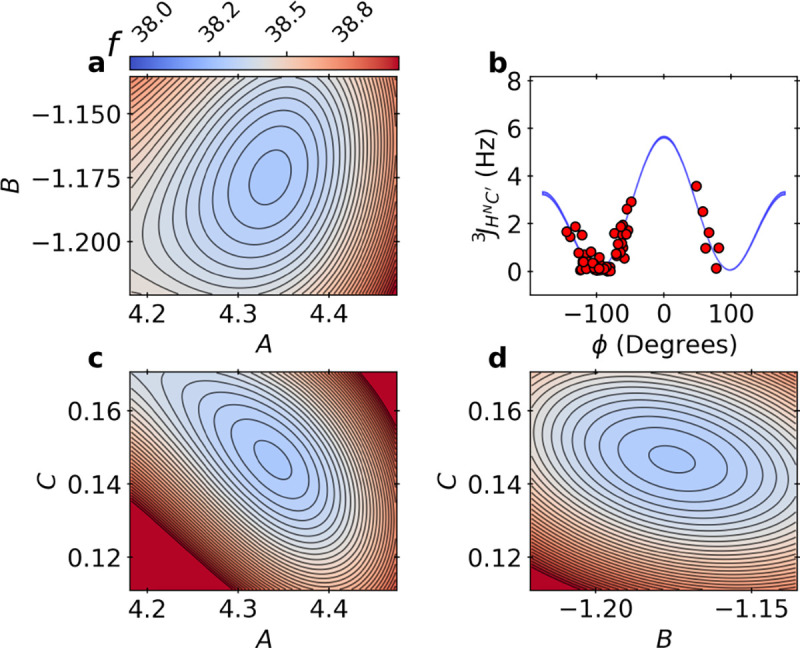
Landscapes of the BICePs score with respect to the predicted Karplus coefficients for JHNC′3. Panels a, c and d illustrate the energy landscape f for pairs of Karplus coefficients when using the 1D3Z structural ensemble during refinement.

In Figure 4, we show the free energy landscape, which is also equivalent to the BICePs score landscape $f_{\xi =0\to 1}$. The Karplus curve for JHNC′3 was found to overlap strongly with the results obtained by SVD when using ϕ angles from the X-ray crystal structure (Figure S9). Red data points are shown using the experimental J-couplings with ϕ angles derived from X-ray crystal pose 1UBQ^30^. The joint BICePs score landscape for the six sets of parameters is too complex to visualize. In an attempt to do our best, we constructed a smooth 2-D landscape for each pair of parameters within a set of scalar couplings by training a Gaussian process on the BICePs energy trace using a radial basis function (RBF) kernel. The landscape matches the computed BICePs scores, and shows minima in the correct locations. All BICePs score landscapes for each of the six sets of Karplus coefficients are illustrated in Figure S14.

**Figure 5.**
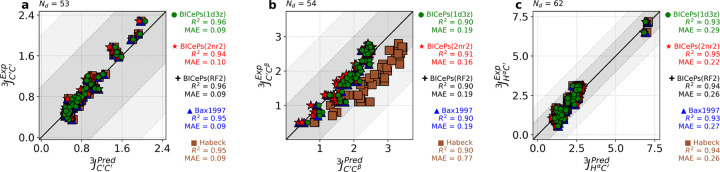
Validation of BICePs-predicted Karplus coefficients perform similarly to Bax1997 and achieve minor improvements over Habeck2005 for scalar coupling predictions for the simulated ensemble of CHARMM22*. Each panel for (a) JHαC′3, (b) JC′Cβ3, and (c) JC′C′3 shows strong correlations between predictions and experiment. Karplus coefficients derived from BICePs using the 2NR2 ensemble gives the best performance for CHARMM22*. For the remaining sets of J-coupling, please see Figure S17.

To demonstrate the transferability across different generative models and validate our parameters, we evaluated the accuracy of the back-calculated scalar couplings using the different sets of Karplus coefficients. In Figure 5, we illustrate how the various sets of parameters derived from different techniques and different structural ensembles exhibit similar performance metrics. Interestingly, applying BICePs-refined Karplus parameters to an ensemble generated by a molecular dynamics simulation (CHARMM22*),^29^ some parameter sets are revealed to be more transferable than others. The mean absolute error (MAE) and coefficient of determination (*R*^2^) for all six types of scalar couplings across different structural ensembles are shown in Figures S15-S17. On average, the BICePs-refined parameters derived from the 2NR2 ensemble (BICePs(2NR2)) give the lowest MAE between experiment and predictions for the CHARMM22* simulated ensemble, closely followed by BICePs(RF2) parameters, whereas Habeck 2005 has the highest due to known difficulties with JC′Cβ3.

To objectively quantify which parameters produce the best predictions for ubiquitin, we compute BICePs scores, $f_{\xi =0\to 1}$ for each of the structural ensembles. This score directly relates to the quality of FM parameters and their predictive accuracy at reproducing experimental scalar couplings, while taking into consideration all sources of potential error. Lower BICePs scores indicate better agreement with experiment. Each row in Table II corresponds to BICePs scores using all six sets of Karplus coefficients used on different structural ensembles. The lowest score is shown in bold.

BICePs scores, $f_{\xi =0\to 1}$ were computed to objectively rank the quality of FM parameters and their predictive accuracy at reproducing experimental scalar couplings (Table II). The left-most column in Table II corresponds to the parameters, where *BICePs(1D3Z)* are the parameters in Table I (set 1), which used 1D3Z ensemble to obtain Karplus coefficients. BICePs score columns, e.g., $f_{\xi =0\to 1}^{1d3z}$ corresponds to BICePs scores evaluated for the 1D3Z ensemble. That is, the superscript corresponds to the structural ensemble used as a validation step. BICePs scores, f for each structural ensemble over all sets of parameters, averaged over five independent rounds of validation each. BICePs calculations burned for 1k steps, followed by 50k steps of MCMC sampling.

Note that the BICePs score is an extensive quantity that grows linearly with the number of replicas. For this reason, our results report the *reduced* BICePs score, $f(\theta )/N_{r}$. We can confirm that the BICePs score, $f_{\xi =0\to 1}^{1\;d3z}=38.14\pm 0.08$ (Table II) is equivalent (within error) with the most probable landscape basin f=38.15±0.19 from sampling the energy landscape, computed as an averarge across four chains; an example for one chain is shown in Figure 4. This is additional evidence of the algorithm’s reliability and quality of the BICePs score, corroborating that the results from variational minimization of the BICePs score and full joint posterior sampling are equivalent.

For both the 2NR2 and CHARMM22* structural ensembles, Bax 1997, BICePs(RF2) and BICePs(1D3Z) parameters give very similar BICePs scores, which suggests robust accuracy of FM parameters in reproducing experimental scalar couplings and the transferability of FM parameters across different prior structural ensembles. Furthermore, when it comes to the CHARMM22* simulated ensemble, the BICePs(2NR2) parameters give the lowest BICePs score. However, it is important to note that the structural ensemble 2NR2 was generated using CHARMM22 force field with additional experimental restraints during simulation.

It is difficult to say which of the model parameters are the best for ubiquitin, so we compare the top four: BICePs(RF2), Bax 1997, BICePs(1D3Z), and BICePs(2NR2). The BICePs(2NR2) parameters are objectively better at predicting J-couplings from structures of ubiquitin generated from simulations using CHARMM22* force field. Futhermore, our BICePs(RF2) parameters have slightly better transferability across structural ensembles and have a better BICePs score over Bax 1997 parameters. In general, when looking across structural ensembles, the lowest BICePs scores come from the 1D3Z structural ensemble $\left(f_{\xi =0\to 1}^{1d3z}\right)$ except for BICePs parameters derived from the 2NR2 ensemble (BICePs(2NR2)). This confirms that the 1D3Z structural ensemble gives the strongest agreement with experimental NMR observations.

#### Ensembles from generative models like RosettaFold2 can be used for parameter refinement.

The booming field of machine learning and artificial intelligence is swiftly transforming the field of modeling structure and dynamics in biological systems. Recent advancements in generative models, such as AlphaFold^31^, RosettaFold^28^ and others, have heralded a new era in the accurate prediction of structural ensembles. Leveraging the predictive power of these models as structural priors is expected to help refine ensemble predictions when integrated with similar algorithms to BICePs^32^. Here, we have demonstrated that structural ensembles generated from RosettaFold2 (RF2) can be reweighted to better align with experimental measurements, while simultaneously refining Karplus parameters. Validation of these parameters by the BICePs score and other statistics demonstrates improved accuracy across a varity of structural ensembles of ubiquitin.

#### Automatic determination of unknown errors.

Our method provides a notable advantage by automatically estimating all potential error sources throughout the ensemble refinement process. This estimation is facilitated through the analysis of posterior distributions, which are instrumental in deriving accurate error assessments for the Karplus coefficients. Consequently, this negates the need for cross-validation techniques commonly used in other approaches^1,33^.

In the context of model validation, the BICePs score emerges as a superior metric over the traditional $\chi^{2}$ test. Unlike $\chi^{2}$, which presupposes a fixed and known error, BICePs dynamically ascertains the level of uncertainty, thereby providing a more nuanced and accurate measure of model quality.

#### Bayesian ranking of Karplus-type relations

The Karplus equation, a cornerstone for interpreting NMR spectroscopy data, comes in multiple forms to accommodate the diverse characteristics of molecular structures, from rigid to flexible^34^. The BICePs algorithm can determine coefficients and their uncertainties for any functional form, including those with additional parameters. Although we do pursue this aim in our current work, it is straightforward to use Bayesian model selection to objectively rank empirical models based on their BICePs scores, while automatically accounting for model complexity, thus providing a balance of model accuracy and parsimony.

#### Adaptive variance as simulated annealing.

As an alternative approach to determine optimal FM parameters, we propose that future work might utilize an annealing approach, in which the variance parameter $\sigma^{2}$ is akin to the temperature. Initially, a high $\sigma^{2}$ would enable extensive exploration of the parameter space to circumvent local optima. This exploration phase mimics the high-temperature regime in annealing, allowing for a broad search. Subsequently, we suggest a schedule of stepwise reduction in $\sigma^{2}$, similar to cooling in simulated annealing, to gradually narrow the search area and determine the optimum solution. This method balances between wide-ranging search and focused refinement, potentially enhancing search efficiency and robustness in FM parameter optimization.

## IV. CONCLUSION

In the quest for accurate forward model predictions, specifically for J-coupling, researchers often navigate the vast literature seeking Karplus parameters that align with their specific systems, occasionally settling for less-than-ideal solutions. Our work demonstrates BICePs as a robust tool for determining forward model (FM) parameters by sampling over their full posterior distribution. We used a toy model to demonstrate that variational minimization of the BICePs score is also a valid approach for FM parameter refinement.

We have shown how the BICePs score–the free energy of “turning on” the restraints tethering the forward model predictions to the experimental values–serves as an effective validation metric for FM parameters. Using structural ensembles and experimental data for ubiquitin, BICePs determined six different sets of Karplus coefficients using different types of J-coupling measurements, while effectively addressing both random and systematic errors. From these results, one can see how this algorithm can be applied more generally to find other optimal forward model parameters. These advances not only contribute to the refinement of molecular simulations but also hold promise for a wide range of applications within the scientific community, particularly among those analyzing structural dynamics and performing model validation.

## Supplementary Material

Supplement 1

## Figures and Tables

**Table I.** Coefficients for the Karplus relation J3ϕ=Acos2ϕ+ϕ0+Bcos(ϕ+ϕ0)+C, determined by BICePs sampling the joint posterior of FM parameters.

		$\phi_{0}$	A (Hz)	B (Hz)	C (Hz)

JC′C3	1	0°	1.71±0.02	−0.85±0.01	0.54±0.00
	2	0°	1.30±0.03	−0.91±0.01	0.62±0.01
	3	0°	1.62±0.03	−0.87±0.01	0.63±0.01
JC′Cβ3	1	60°	1.83±0.04	0.34±0.05	0.41±0.02
	2	60°	2.20±0.04	0.34±0.04	0.04±0.02
	3	60°	1.81±0.04	0.38±0.04	0.31±0.02
JHαC′3	1	120°	3.64±0.02	−2.14±0.02	1.27±0.02
	2	120°	4.10±0.03	−2.00±0.02	0.95±0.02
	3	120°	3.78±0.02	−2.12±0.02	1.21±0.02
JHNC′3	1	180°	4.33±0.04	−1.17±0.01	0.14±0.01
	2	180°	4.60±0.12	−0.57±0.03	−0.10±0.01
	3	180°	4.57±0.09	−1.20±0.03	0.13±0.01
JHNCβ3	1	60°	2.72±0.03	−0.35±0.03	0.12±0.01
	2	60°	3.00±0.04	−0.26±0.03	−0.28±0.02
	3	60°	2.52±0.03	−0.03±0.02	−0.09±0.02
JHNHα3	1	−60°	7.11±0.05	−1.38±0.03	1.43±0.04
	2	−60°	7.50±0.07	−1.50±0.02	1.50±0.06
	3	−60°	6.97±0.07	−1.49±0.04	1.63±0.05

1 1D3Z as the structural ensemble

2 2NR2 as the structural ensemble

3 RosettaFold2 (RF2) as the structural ensemble

**Table II.** BICePs scores (32 replicas), *f* for each structural ensemble over all sets of parameters, averaged over five independent rounds of validation each.

Parameters	$f_{\xi =0\to 1}^{1d3z}$	$f_{\xi =0\to 1}^{2nr2}$	$f_{\xi =0\to 1}^{\mathrm{CHARMM22*}}$

Bax 1997^2,26^	61.12±0.08	132.49±0.09	99.27±1.91
Habeck 2005^3^	135.66±0.08	199.32±0.24	165.64±0.47
BICePs(1D3Z)	**38.14±0.08**	141.69±0.16	105.00±0.74
BICePs(2NR2)	118.42±0.14	**113.15±1.34**	**76.27±0.66**
BICePs(RF2)	68.07±0.60	129.42±0.15	88.04±0.21
