# Automatic Forward Model Parameterization with Bayesian Inference of Conformational Populations

**Published:** 2025-06-18

**Authors:** Robert M. Raddi, Tim Marshall, Vincent A. Voelz

**Affiliations:** Department of Chemistry, Temple University, Philadelphia, PA 19122, USA

**Keywords:** Bayesian Inference, Conformational Populations, Forward Model Optimization, Karplus Relation, Neural Network-based Forward Models, Machine Learning for Structural Biology

## Abstract

To quantify how well theoretical predictions of structural ensembles agree with experimental measurements, we depend on the accuracy of forward models. These models are computational frameworks that generate observable quantities from molecular configurations based on empirical relationships linking specific molecular properties to experimental measurements. Bayesian Inference of Conformational Populations (BICePs) is a reweighting algorithm that reconciles simulated ensembles with ensemble-averaged experimental observations, even when such observations are sparse and/or noisy. This is achieved by sampling the posterior distribution of conformational populations under experimental restraints as well as sampling the posterior distribution of uncertainties due to random and systematic error. In this study, we enhance the algorithm for the refinement of empirical forward model (FM) parameters. We introduce and evaluate two novel methods for optimizing FM parameters. The first method treats FM parameters as nuisance parameters, integrating over them in the full posterior distribution. The second method employs variational minimization of a quantity called the BICePs score that reports the free energy of “turning on” the experimental restraints. This technique, coupled with improved likelihood functions for handling experimental outliers, facilitates force field validation and optimization, as illustrated in recent studies (Raddi et al. 2023, 2024). Using this approach, we refine parameters that modulate the Karplus relation, crucial for accurate predictions of *J*-coupling constants based on dihedral angles (*φ*) between interacting nuclei. We validate this approach first with a toy model system, and then for human ubiquitin, predicting six sets of Karplus parameters for ^3^*J_H^N^H^α^_*, ^3^*J_H^α^C′_*, ^3^*J_H^N^C^β^_*, ^3^*J_H^N^C′_*, ^3^*J_C′C^β^_*, ^3^*J_C′C′_*. Finally, we demonstrate that our framework naturally generalizes optimization to any differentiable forward model, such as those constructed with neural networks. This approach, which does not rely on any predetermined parameterization, enhances predictive accuracy and can be used for many applications.

## Graphical Abstract

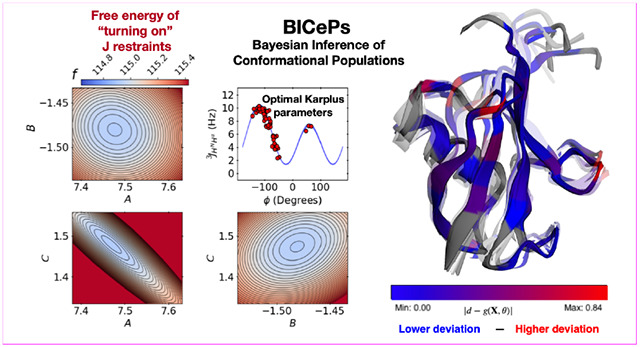

## I. INTRODUCTION

In the field of molecular modeling and dynamics, the accuracy of theoretical predictions is crucial. The quantitative agreement between theory and experiment is typically characterized through the use of accurate *forward models*—computational frameworks that predict observable quantities from molecular configurations. These models often depend on empirical relationships that link specific molecular properties to experimental measurements.

Model validation and refinement of structural ensembles against NMR observables critically depends on reliable forward models (FMs) that have been robustly parameterized to minimize FM error. In refining these FMs, it is important to consider random and systematic errors inherent to the experimental data, which in turn contribute to overall uncertainty of integrative models. It is usually challenging to obtain complete knowledge of such errors; instead, such errors typically need to be inferred from the data.

A further challenge is presented by missing or insufficient examples of known structures that can be used to train forward models. For NMR observables that depend on backbone *ϕ*-angles, such as *J*-coupling constants, the reference data from X-ray crystallography may be missing or dynamically averaged, creating large uncertainties in the correct *ϕ*-angles. Numerous approaches have been developed to address some of these challenges.^1–4^ Some algorithms rely heavily on X-ray crystal structure data; others have many hyperparameters that need to be determined.

To address these challenges, we extend the Bayesian Inference of Conformational Populations (BICePs) algorithm^5,6^ to refine FM parameters. BICePs is a reweighting algorithm to refine structural ensembles against sparse and/or noisy experimental observables, which has been used in many previous applications.^7–10^ BICePs infers all possible sources of error by sampling the posterior distribution of these parameters directly from the data through MCMC sampling. BICePs also computes a free energy-like quantity called the BICePs score that can be used for model selection and model parameterization.^6,11,12^

Recently, BICePs was enhanced with a replica-averaging forward model, making it a maximum-entropy (MaxEnt) reweighting method, and unique in that no adjustable regularization parameters are required to balance experimental information with the prior.^6^ With this new approach, the BICePs score becomes a powerful objective function to parameterize optimal models. Here, we show that the BICePs score, which reflects the total evidence for a model, can be used for variational optimization of FM parameters. The BICePs score contains a form of inherent regularization, and is particularly powerful when used with specialized likelihood functions that allow for the automatic detection and down-weighting of the importance of experimental observables subject to systematic error.^6^

To effectively refine FM parameters, we use BICePs to sample over the entire posterior distribution of FM parameters, enabling simultaneous ensemble reweighting and parameter refinement. Additionally, we show that by variational minimization of the BICePs score, we obtain the same result and show that the two approaches are equivalent, with each method requiring particular considerations. We first demonstrate our method’s effectiveness using a toy model and then apply it to optimize six distinct sets of Karplus parameters for the human protein ubiquitin. As a proof-of-concept for more complex models, we also train a neural network to learn *J*-coupling constants from dihedral angles using the BICePs score as a loss function. We then discuss how this idea could be applied to neural networks for chemical shift prediction.^13–16^ Of course, our framework naturally extends to other empirical forward models with tunable parameters—for example, hydrogen-deuterium exchange (HDX) models with adjustable hydrogen-bonding and burial weights, or paramagentic relaxation enhancement (PRE) models with system-specific correlation times and scaling factors. These examples illustrate the generality and flexibility of our approach for refining a wide range of forward models to improve agreement between simulations and experiment.

## II. THEORY

In this section, we develop two different theoretical approaches to optimize forward models. In the first method, we include forward model parameters in the posterior, and sample the full posterior distribution of all parameters. In the second approach, we optimize the forward model by minimizing the BICePs score, a free energy-like quantity that can be used for variational optimization.

### A. Forward model optimization by posterior sampling of forward model parameters

BICePs uses a Bayesian statistical framework, inspired by Inferential Structure Determination (ISD)^17^, to model the posterior distribution *p*(*X, σ*), for conformational states *X*, and nuisance parameters *σ*, which characterize the extent of uncertainty in the experimental observables *D*:

(1) p(X,σ∣D)∝p(D∣X,σ)p(X)p(σ).

Here, p(D∣X,σ) is a likelihood function that uses a forward model to enforce the experimental restraints, p(X) is a prior distribution of conformational populations from some theoretical model, and $p(\sigma )∼\sigma^{-1}$ is a non-informative Jeffrey’s prior.

For a specific forward model *g*(*X, θ*) with a set of FM parameters *θ* = (*θ*_1_, *θ*_2_, …, *θ_m_*), where *m* is the number of parameters, we can additionally include the parameters in the posterior,

(2) p(X,σ,θ∣D)∝p(D∣X,σ,θ)p(X)p(σ)p(θ).

#### Replica-averaging.

When BICePs is used with a replica-averaged forward model, it becomes a MaxEnt reweighting method in the limit of large numbers of replicas^18–23^. Consider a set of *N* replicas, $X=\left\{X_{r}\right\}$, where $X_{r}$, is the conformational state being sampled by replica *r*. To compare the sampled replicas with ensemble-averaged experimental observables, we define a replica-averaged forward model $g(X,\theta )=\frac{1}{N}\sum_{r}^{N}g\left(X_{r},\theta \right)$. This quantity is an estimator of the true ensemble average, with an error due to finite sampling for the observable *j* estimated using the standard error of the mean (SEM):^18,23^ $\sigma_{j}^{SEM}=\sqrt{\frac{1}{N}\sum_{r}^{N}{\left(g_{j}\left(X_{r},\theta \right)-\left\langle g_{j}(X,\theta )\right\rangle \right)}^{2}}$. Thus, $\sigma_{j}^{\text{SEM}}$ decreases as the square root of the number of replicas.

For a single forward model with parameters *θ*, the joint posterior distribution for all parameters is

(3) $$p(X,\sigma ,\theta |D)\propto \prod_{r=1}^{N}p\left(X_{r}\right)p(D|g(X,\theta ),\sigma )p(\sigma )p(\theta )$$

where **X** is a set of *N* conformation replicas. In many cases, we wish to optimize several forward models at once. For example, there are unique sets of Karplus parameters in the forward models for vicinal *J*-coupling constant observables ^3^*J_H^N^H^α^_*, ^3^*J_H^N^C′_*,^3^*J_H^N^C^β^_*, etc. If there are *K* different forward models $\theta^{\left(1\right)},\theta^{\left(2\right)},\ldots \theta^{\left(K\right)}$, each with their own set of parameters and error distributions, we can describe their full joint posterior distribution as

(4) $$p\left(X,\sigma ,\theta^{\left(1\right)},\theta^{\left(2\right)},\ldots \theta^{\left(K\right)}|D\right)\;\propto \prod_{r=1}^{N}p\left(X_{r}\right)\prod_{k=1}^{K}p\left(D_{k}|g\left(X,\theta^{\left(k\right)}\right),\sigma_{k}\right)p\left(\sigma_{k}\right)p\left(\theta^{\left(k\right)}\right)$$

where $D_{k}$ are the experimental observables predicted by the $k^{\mathrm{th}}$ forward model, $\theta^{\left(k\right)}$ are the parameters for the $k^{\mathrm{th}}$ FM, and $\sigma_{k}=\sqrt{{\left(\sigma_{k}^{SEM}\right)}^{2}+{\left(\sigma_{k}^{B}\right)}^{2}}$ describing the total error for the $k^{\mathrm{th}}$ observable. The total error originates from both finite sampling through the standard error of the mean ($\sigma_{k}^{SEM}$), and uncertainty in the experimental measurements, known as a Bayesian uncertainty parameter $\sigma_{k}^{B}$. The prior distribution of uncertainties $p\left(\sigma_{k}\right)$ is treated as a non-informative Jeffrey’s prior ($\sigma_{k}^{-1}$) for each collection of observables, and the posterior of FM parameters p(θ∣D) is recovered by marginalization over all X and *σ*:

(5) $$p(\theta |D)=\sum_{X}\int \;p(X,\sigma ,\theta |D)d\sigma$$

#### Gradients speed up convergence.

In our methodology, Markov chain Monte Carlo (MCMC) is used to sample the posterior with acceptances following the Metropolis-Hastings criterion. Our algorithm can be used with or without gradients. However, significantly faster convergence, especially in higher dimensions, is achieved through an integration of stochastic gradient descent approach. Our gradient descent approach allows for informed updates to the FM parameters, incorporating stochastic noise to facilitate the escape from local minima and enhance exploration of the parameter space.

The update mechanism is succinctly encapsulated in the equation:

(6) $$\theta_{\text{trial}}=\theta_{\text{old}}-l_{\text{rate}}\cdot \nabla u+\eta \cdot 𝒩(0,1)$$

where $\theta_{\text{trial}}$ and $\theta_{\text{old}}$ denote the trial parameters and previous parameters, respectively. The learning rate is denoted by $l_{\text{rate}}$, ∇u signifies the calculated gradient of BICePs energy function u=−lnp(X,σ,θ∣D) with respect to the parameters θ, and η scales the noise drawn from a standard normal distribution 𝒩(0,1).

This parameter update strategy is designed to satisfy the Metropolis-Hastings criterion, ensuring that each step in the parameter space not only moves towards minimizing the energy of the forward model but also adheres to the probabilistic acceptance of potentially non-optimal moves to avoid local optima traps. Ergodic sampling is ensured by “turning off” the gradient after burn-in. The sampling procedure involves: (1) acquiring derivatives of the FM parameters, (2) perturbing these parameters based on the derived information, (3) predicting observables using perturbed FM parameters and computing the total energy, and (4) assessing the new energy against the previous to determine acceptance based on the Metropolis-Hastings criterion. This ensures a thorough and effective search of the parameter space, taking advantage of both the landscape topology and stochastic elements to guide the exploration.

#### The Good-Bad model accounts for systematic error due to outlier measurements.

Recent work has developed sophisticated likelihood models for BICePs, designed to detect outliers, that are robust in the presence of systematic error^6^ Raddi et al. (2025) developed two likelihood models for this purpose: the Student’s model and the Good-Bad model. In related work using BICePs to perform automated force field optimization, we used the Student’s model.^7^ Here, in this work, we use the Good-Bad model, primarily because the derivatives of the Good-Bad model are less complicated than the Student’s model.

The Good-Bad likelihood model^6^ assumes that the level of noise is mostly uniform, except for a few erratic measurements. This limits the number of uncertainty parameters that need to be sampled, while still capturing outliers. Consider a model in which the uncertainties $\sigma_{j}$ for particular observables *j* are distributed with respect to some typical uncertainty $\sigma^{B}$ according to a conditional probability $p\left(\sigma_{j}|\sigma^{B}\right)$. We derive a posterior for the parameters of the *k^th^* FM having a single uncertainty parameter $\sigma^{B}$ by marginalizing over all $\sigma_{j}$,

(7) $$p\left(X,\sigma_{0},\theta^{\left(k\right)}|D\right)\propto \prod_{r=1}^{N}p\left(X_{r}\right)\prod_{j=1}^{N_{d}}\int_{\sigma^{SEM}}^{\infty }p\left(d_{j}|g_{j}\left(X,\theta^{\left(k\right)}\right),\sigma_{j}\right)p\left(\sigma_{j}|\sigma_{0}\right)d\sigma_{j}$$

where $\sigma_{0}=\sqrt{{\left(\sigma^{B}\right)}^{2}+{\left(\sigma^{SEM}\right)}^{2}}$. Under the Good-Bad model, we say that the “good” data consist of observables normally distributed about their true values with effective variance $\sigma_{0}^{2}$, while the “bad” data are subject to systematic error, leading to a larger effective variance $\varphi^{2}\sigma_{0}^{2}$, where φ≥1.

By this assignment, $p\left(\sigma_{j}|\sigma_{0}\right)$ from equation 7 becomes

(8) $$p\left(\sigma_{j}|\sigma_{0},\omega ,\varphi \right)=\omega \delta \left(\sigma_{j}-\varphi \sigma_{0}\right)+(1-\omega )\delta \left(\sigma_{j}-\sigma_{0}\right)$$

where 0≤ω<1 describes the fraction of “bad” observables. Since the value of ω is unknown, it is treated as a nuisance parameter, and marginalized over its range. The resulting posterior is

(9) $$p\left(X,\sigma_{0},\varphi ,\theta^{\left(k\right)}|D\right)\propto \prod_{r=1}^{N}\left\{p\left(X_{r}\right)\prod_{j=1}^{N_{d}}\int_{0}^{1}d\omega \int_{\sigma^{\text{SEM}}}^{\infty }\text{exp}\left(-\frac{{\left(d_{j}-g_{j}\left(X,\theta^{\left(k\right)}\right)\right)}^{2}}{2\sigma_{j}^{2}}\right)\frac{\omega \delta \left(\sigma_{j}-\varphi \sigma_{0}\right)+(1-\omega )\delta \left(\sigma_{j}-\sigma_{0}\right)}{\sqrt{2\pi }\sigma_{j}}d\sigma_{j}\right\}\;=\prod_{r=1}^{N}\left\{p\left(X_{r}\right)\prod_{j=1}^{N_{d}}\left(\frac{1-H\left(\sigma^{SEM}-\sigma_{0}\right)}{2}𝒩\left(g_{j}\left(X,\theta^{\left(k\right)}\right)|d_{j},\sigma_{0}^{2}\right)+\frac{1-H\left(\sigma^{SEM}-\varphi \sigma_{0}\right)}{2\varphi }𝒩\left(g_{j}\left(X,\theta^{\left(k\right)}\right)|d_{j},\varphi^{2}\sigma_{0}^{2}\right)\right)\right\},$$

where H is the Heaviside step function. After marginalization, we are left with the Bayesian uncertainty parameter $\sigma_{0}^{B}$, and an additional parameter φ. Both parameters are sampled in the posterior. When φ=1, the model reverts to a Gaussian likelihood model. When considering the full posterior, this extra nuisance parameter is given a non-informative Jeffrey’s prior, $p(\varphi )∼\varphi^{-1}$.

For a single set of FM parameters (for simplicity), the BICePs energy function, $u=-log\;p\left(X,\sigma_{0},\varphi ,\theta^{\left(k\right)}|D\right)$, the negative logarithm of the posterior in its full form is given by

(10) $$u=\sum_{r=1}^{N}-\text{log}\left(p\left(X_{r}\right)\right)-N\sum_{j=1}^{N_{d}}\text{log}\left[\frac{1-H\left(\sigma^{SEM}-\sigma_{0}\right)}{2}𝒩\left(g_{j}\left(X,\theta^{\left(k\right)}\right)|d_{j},\sigma_{0}^{2}\right)+\frac{1-H\left(\sigma^{SEM}-\varphi \sigma_{0}\right)}{2\varphi }𝒩\left(g_{j}\left(X,\theta^{\left(k\right)}\right)|d_{j},\varphi^{2}\sigma_{0}^{2}\right)\right].$$

When φ=1, this energy function is the same the result we would get if we used a Gaussian likelihood function,

(11) $$u=\sum_{r=1}^{N}-\text{log}\left(p\left(X_{r}\right)\right)+N\left[\overset{N_{d}}{\underset{j=1}{\sum }}-\text{log}\left(\frac{1}{\sqrt{2\pi }\sigma_{0}}\right)+\frac{{\left(d_{j}-g_{j}(X,\theta )\right)}^{2}}{2\sigma_{0}^{2}}-\text{log}\left(p\left(\sigma_{0}\right)\right)\right].$$

The first derivative of equation 10 with respect to the $i^{\mathrm{th}}FM$ parameter in the parameter set $\theta^{\left(k\right)}$ is

(12) $$\frac{\partial u}{\partial \theta_{i}^{\left(k\right)}}=N\sum_{j=1}^{N_{d}}\frac{\partial g_{j}\left(X,\theta^{\left(k\right)}\right)}{\partial \theta_{i}^{\left(k\right)}}\frac{\left(d_{j}-g_{j}\left(X,\theta^{\left(k\right)}\right)\right)}{\sigma_{0}^{2}}\left(\frac{\varphi^{2}\left(1-H\left(\sigma^{SEM}-\sigma_{0}\right)\right){𝒩}_{\varphi^{2}}^{-1}+\left(1-H\left(-\varphi \sigma_{0}+\sigma^{SEM}\right)\right){𝒩}_{1}^{-1}}{\varphi^{2}\left(H\left(\sigma^{SEM}-\sigma_{0}\right)-1\right){𝒩}_{\varphi^{2}}^{-1}+\varphi^{2}\left(H\left(-\varphi \sigma_{0}+\sigma^{SEM}\right)-1\right){𝒩}_{1}^{-1}}\right),$$

where ${𝒩}_{1}^{-1}=𝒩{\left(g_{j}\left(X,\theta^{\left(k\right)}\right)|d_{j},\sigma_{0}^{2}\right)}^{-1}$ and ${𝒩}_{\varphi^{2}}^{-1}=𝒩{\left(g_{j}\left(X,\theta^{\left(k\right)}\right)|d_{j},\varphi^{2}\sigma_{0}^{2}\right)}^{-1}$. Both represent the reciprocal of Gaussian likelihoods, where ${𝒩}_{1}^{-1}$ and ${𝒩}_{\varphi^{2}}^{-1}$ denote inverse-weighted forms of normal distributions with variances $\sigma_{0}^{2}$ and $\varphi^{2}\sigma_{0}^{2}$, respectively.

When φ=1, the gradient becomes

(13) $$\frac{\partial u}{\partial \theta_{i}^{\left(k\right)}}=-N\overset{N_{d}}{\underset{j=1}{\sum }}\frac{\partial g_{j}\left(X,\theta^{\left(k\right)}\right)}{\partial \theta_{i}^{\left(k\right)}}\frac{\left(d_{j}-g_{j}\left(X,\theta^{\left(k\right)}\right)\right)}{\sigma_{0}^{2}}.$$

Second derivatives of the BICePs energy function and the BICePs score are useful for descent and uncertainty quantification using other forward models. We refrain from writing out the second derivative here, but the derivation is straightforward. For the forward models we consider below (Karplus relations), all have second derivatives that go to zero. For more general cases, see Appendix V for more details. The energy of the Good-Bad likelihood model and its first and second derivatives are shown in Figure S1.

### B. Forward model optimization by variational minimization of the BICePs score

As we show in Results and Discussion, treating the forward model parameters as nuisance parameters and sampling them as part of the full posterior is an efficient strategy to refine FM parameters. However, in the limit of large numbers of FM parameters, the dimensionality of the posterior may become unwieldy and may suffer from the “curse of dimensionality”.

As an alternative strategy, we consider optimization of forward model parameters using variational minimization of the BICePs score, an approach that we have used to perform automated force field optimization^12^. In this approach, the FM parameters are no longer part of the joint posterior density. Instead, the posterior is conditioned on the set of FM parameters *θ*, that is, equation 2 becomes

(14) p(X,σ∣D,θ)∝p(D,θ∣X,σ)p(X)p(σ)

In this view, ensemble refinement is performed with a static set of FM parameters for each BICePs calculation. The BICePs score then enables the selection of the optimal model parameterized by *θ*.

BICePs evaluates model quality by calculating a free energy-like quantity called the BICePs score. For a forward model with parameters *θ*, the BICePs score *f* (*θ*) is computed as the negative logarithm of a Bayes factor comparing the total evidence of a given model against a well-defined reference, marginalizing over all uncertainty,

(15) $$f(\theta )=-ln\left(Z(\theta )/Z_{0}\right),$$

where

(16) Z(θ)=∬exp(−u(X,σ∣D,θ))dXdσ

is the evidence for FM parameters *θ*, $Z_{0}$ is the evidence for a suitable reference state, and *u* is the unchanged BICePs energy function (equation 10). To construct the reference state, we consider a series of likelihoods $p_{\xi }(D,\theta |X,\sigma )∼[p(D|X,\sigma ){]}^{\xi }$ parameterized by *ξ* ∈ [0,1], and set the reference state as the thermodynamic ensemble corresponding to *ξ* = 0. The BICePs score is then calculated as the free energy of “turning on” experimental restraints (*ξ* = 0→1).

It should be noted that in other applications of BICePs,^6,12^ the reference state for the BICePs score is defined using the *λ* = 0 state for a series of a priors $p_{\lambda }(X)∼[p(X){]}^{\lambda }$, and the BICePs score is computed as the free energy of (*λ* = 0→1) and (*ξ* = 0→1) transformations. Here, since we are only interested in evaluating and/or parameterizing the likelihood functions, we set *p*(*X*) to be uniform.

To optimize forward model parameters, the BICePs score is used as an objective function that is estimated given a candidate value of *θ*. Based on the estimate (and possibly other previous estimates), new candidates for *θ* are iteratively proposed and accepted or rejected, with the goal of finding the values θ* that minimize the BICePs score.

For this purpose, gradient-based searches are very helpful. Conveniently, the derivative of the BICePs score *f* (*θ*) with respect to the FM parameters reduces to the Boltzmann-averaged values of the gradient of the *∂u/∂θ*:

(17) $$\frac{\partial f(\theta )}{\partial \theta_{i}}=\iint \frac{1}{Z(\theta )}\left[\frac{\partial u}{\partial \theta_{i}}\right]exp(-u)dXd\sigma =\left\langle \frac{\partial u}{\partial \theta_{i}}\right\rangle$$

In our results below, we use the first-order optimization method L-BFGS-B,^24^ which only requires first derivatives. For more complex forward models, second-order methods may be needed. The Hessian matrix (of second partial derivatives of the BICePs score with respect to FM parameters) can be used for uncertainty estimation when *f* (*θ*) is convex. Assuming this is true, we estimate uncertainties in the FM model parameters using covariances obtained through inversion of the Hessian. Conceptually, this means that the estimated uncertainties in the best-fit values represent the widths of the basins in the BICePs score landscape. For non-convex problems, this can sometimes lead to an under-estimation of uncertainty depending upon the curvature of the basins of the local minima in the BICePs score landscape.

Calculation of the BICePs score (a free energy difference) and its derivatives (expectation values of energy derivatives) is performed using the MBAR free energy estimator,^25^ by sampling at several intermediates *ξ* = 0→1, which enables accurate estimates of all quantities. The accuracy of the BICePs score depends on converged sampling and sufficient thermodynamic overlap of intermediates (*ξ* = 0→1) in the BICePs computation. To ensure strong overlap, we optimize the *ξ*-values by spacing ensembles equidistantly in thermodynamic length,^26,27^ employing a strategy akin to the “thermodynamic trailblazing” method proposed by Rizzi et al.^28^ This optimization is performed using an algorithm we have developed called pylambdaopt.^29^

## III. RESULTS

### A. toy model to test the performance of forward model optimization

**Figure 1.**
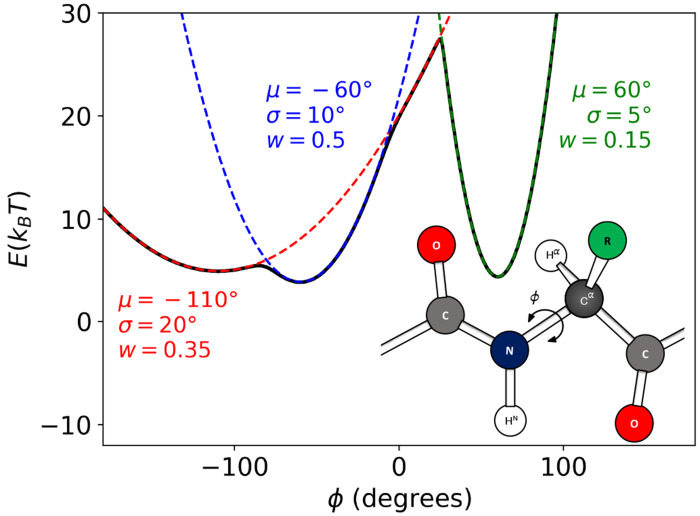
A toy model for measuring the performance of forward model optimization. The *ϕ*-angles for each conformational state is pulled from a multi-modal distribution and corresponding energies. (a) This multi-modal distribution of *ϕ*-angles was intended to represent configurations with different secondary structure elements having three distinct modes described by the mean (*μ*), standard deviation (σ) and weight (*w*): beta sheets (*μ* = −110°, σ = 20°, *w* = 0.35), right-handed helices (*μ* = −60°, σ = 10°, *w* = 0.5), and left-handed helices (μ = 60°, σ = 5°, *w* = 0.15). (b) Cartoon representation of the backbone torsion angle, *ϕ*.

To investigate the performance of BICePs in optimizing forward models, we introduce a simplified, yet physical, toy model. This model is designed to mimic the complexity of protein backbone structures by generating *ϕ*-angles from a multi-modal distribution, thereby emulating configurations characteristic of different secondary structural elements (Figure 1). This distribution encompasses three distinct modes, each characterized by a mean (*μ*), standard deviation (*σ*), and weight (*w*): beta sheets (*μ* = −110°, *σ* = 20°, *w* = 0.35), right-handed helices (μ =−60°, *σ* =10°, *w*=0.5), and left-handed helices (*μ* =60°, *σ* = 5°, *w* = 0.15). These parameters were chosen to accurately reflect the structural variability found in proteins. Angles *φ_i_* were sampled from the multi-modal distribution,

(18) $$p(\phi |\mu ,\sigma )=\sum_{l}w_{l}\frac{1}{\sqrt{2\pi \sigma_{l}^{2}}}\text{exp}\left(-\frac{{\left(\phi -\mu_{l}\right)}^{2}}{2\sigma_{l}^{2}}\right).$$

The sampled $\phi_{i}$ were then used to calculate synthetic ensemble-averaged experimental *J*-coupling constants ^3^*J*(*ϕ*), using the Karplus relation

(19) J3(ϕ)=Acos2(ϕ)+Bcos(ϕ)+C,

with the coefficients set to their chosen *true* values: (A*=6.51, B*=−1.76, C*=1.6). The synthetic *J*-coupling constant observables were calculated using the ensemble-average, as ∑_*i*_^3^*J*(*ϕ_i_*)*p*(*ϕ_i_*).

### Including forward model parameters in the BICePs posterior is a robust method to optimize Karplus parameters in the presence of experimental errors.

Using the toy model described above, we next perform tests to examine whether including forward model parameters in the BICePs posterior can be used to find the optimal Karplus parameters starting from an initial reference forward model with parameters ($A_{0},B_{0},C_{0}$). Through these tests, we evaluate the precision of the results, and how robust the algorithm is in the presence of noise.

To evaluate the robustness of our algorithm in the presence experimental inaccuracies, we introduced random and systematic errors of varying magnitudes ($\sigma_{\text{data}}$) into the synthetic experimental scalar couplings. The performance of our Good-Bad likelihood model, a Gaussian likelihood model, and singular value decomposition (SVD) was compared under these conditions.

#### SVD calculations.

Parameters of many reference Karplus relations were previously determined using a singular value determination approach.^30^ To test this approach against our BICePs method, we derived Karplus parameters *θ* = {A,B,C} using a weighted singular value decomposition (SVD) fitting approach to optimally fit the *J*-coupling values as a function of dihedral angles. For each observation *j* across $N_{d}$ measurements, the matrix *M* was constructed with rows for each ϕ angle:

(20) $$M=\left[\begin{array}{ccc} \sum_{X}p(X){cos}^{2}\left(\phi_{1,X}+\phi_{0}\right) & \sum_{X}p(X)cos\left(\phi_{1,X}+\phi_{0}\right) & 1\\ \sum_{X}p(X){cos}^{2}\left(\phi_{2,X}+\phi_{0}\right) & \sum_{X}p(X)cos\left(\phi_{2,X}+\phi_{0}\right) & 1\\ \vdots & \vdots & \vdots \\ \sum_{X}p(X){cos}^{2}\left(\phi_{N_{d},X}+\phi_{0}\right) & \sum_{X}p(X)cos\left(\phi_{N_{d},X}+\phi_{0}\right) & 1 \end{array}\right]$$

where *p*(*X*) represents the true populations for state *X*, and *ϕ*_0_ is the phase shift of −60°.

SVD was applied to decompose the matrix $M=U\Sigma V^{T}$, and Karplus coefficients were derived using:

(21) $$\theta =V(\Sigma +\varepsilon I{)}^{-1}U^{T}J^{exp},$$

where *ε* = 1 × 10^−6^ is a small regularization term added to the diagonal of Σ to ensure stability of the pseudo-inverse, and $J^{exp}$ represents the vector of experimental *J*-coupling values. This method ensures robust estimation of *θ* under ideal experimental conditions, given the true conformational populations. In practice, the true populations are not known *a priori*. The uncertainty in SVD coefficients was determined through 1000 iterations of fitting, each omitting 10% of the data points chosen at random.

Typical uncertainties in NMR frequency measurements range from 0.1 to 1.0 Hz, primarily influenced by magnetic field strength, instrument quality, sample conditions, and the specifics of the pulse sequence used. In these experiments, 100 conformational states and 60 synthetic experimental scalar couplings were used. We introduced systematic error by shifting the experimental ^3^*J* values by +2.0 Hz to +4.0 Hz for up to 20% of the data points. BICePs calculations were performed by averaging FM parameters over three chains of MCMC starting from different initial parameters ({*A* = 9,*B* = −1,*C* = 1}, {*A* = 4,*B* = 0,*C* = 3}, {*A* = 0,*B* = 0,*C* = 0}). Regardless of different starting parameters, posterior sampling universally converges to “true” optimal FM parameters. In these calculations, we used 32 BICePs replicas, and burned 10k steps followed by 50k steps of MCMC sampling.

We evaluated model performance by the root-mean-square error (RMSE) between the true *J*-coupling values with parameters {A* = 6.51, B* = −1.76, C* = 1.6} and the *J*-coupling values using predicted Karplus coefficients for all 60 synthetic measurements, performed over 1k independent trials of random generations of toy model data. Average RMSE results, computed over 100 BICePs calculations, highlight the algorithm’s robustness and its ability to accurately predict FM parameters even in the presence of data perturbations. Error bars in our results represent the standard deviation across these calculations, providing a comprehensive measure of the algorithm’s reliability under various experimental accuracy.

**Figure 2.**
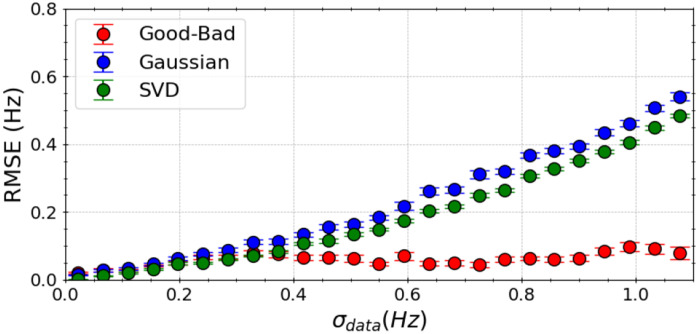
Comparative analysis in performance of the Good-Bad likelihood model (red), a Gaussian likelihood model (blue), and singular value decomposition (SVD) using the “true” *ϕ* angles with synthetic experimental data. Here, we induced random and systematic error of varying magnitude (σ_data_) to the experimental scalar couplings. Model performance was measured by computing RMSE (Hz) between the “true” scalar couplings and the couplings generated from the Karplus relations with predicted Karplus coefficients over 1,500 random perturbations to the experimental data, and represent the average of 100 BICePs calculations. Error bars represent the standard deviation. Predictions from SVD and the Gaussian likelihood model become notably less dependable when data incorporates errors, especially when σ_data_ exceeds 0.5 Hz.

Our findings indicate that the Good-Bad likelihood model (red) exhibits superior insensitivity to experimental errors compared to a traditional Gaussian likelihood model (blue) and SVD (green) approaches (Figure 2). Predictions from SVD and the Gaussian likelihood model become notably less dependable when the experimental data contains errors, especially when σ_data_ exceeds 0.5 Hz. On average, error in predictions (RMSE) from the Good-Bad model does not exceed 0.1 Hz over the full range of $\sigma_{\text{data}}$.

An example of a single trial of forward model parameter refinement using the toy model is shown in Figure S2, where BICePs predicts Karplus coefficients by posterior sampling over FM parameters. Both BICePs and SVD methods successfully reproduce the “true” Karplus curve. However, BICePs can accurately identify the error present in the data ($\sigma_{\text{data}}$ = 0.471 Hz), as indicated in the marginal posterior of uncertainty $p\left(\sigma_{J}\right)$. The BICePs-predicted maximum a posteriori uncertainty was found to be σ*_J_* = 0.272 with a variance scaling parameter of ϕ*_J_* = 1.98. The marginal posterior distributions of FM parameters for the Good-Bad model were {*A* = 6.6 ± 0.04, *B* = −1.8 ± 0.02, *C* = 1.5 ± 0.03}, and for SVD, {*A* = 6.11 ± 0.06, *B* = −1.63±0.04, *C* = 1.80 ± 0.04}.

In addition to the Good-bad model, we refined parameters using the Student’s model ({*A* = 6.8 ± 0.03, *B* = −1.9±0.03, *C* = 1.4 ± 0.03}) to demonstrate that the Student’s model yields similar performance (Figure S3). The computed Gelman-Rubin ($\hat{R}$) statistic^31^ for these calculations was found to be $\hat{R}=1.01$ for each of the marginal posterior distributions of Karplus coefficients, which demonstrates that our chains converge to the same parameter location with similar variance.

Furthermore, we assessed model performance across varying qualities of prior structural ensembles as illustrated in Figure S4. By introducing varying levels of prior error σ_prior_ (measured in degrees) through perturbations to the “true” *ϕ* angles, even in the presence of random and systematic error, we observed strong correlation between the BICePs score and the quality of the structural ensemble, with a coefficient of determination *R*^2^ of 0.99. For these calculations, we employed the Good-Bad model, utilizing 32 replicas, and conducted 1,000 random perturbations to the *ϕ* angles with errors up to σ_prior_ = 4°, and perturbations to the experimental data σ_data_ = 0.68 ± 0.24 Hz.

The comprehensive evaluation of our algorithm with this toy model underscores its efficacy in accurately determining FM parameters, reflecting scenarios commonly encountered in real-world applications. The robust performance of the algorithm, even in the face of random and systematic errors, can be attributed to BICePs’ sophisticated error-handling within its likelihood models. This approach also ensures that predicted FM parameters derived from sub-optimal structural ensembles remain reliable. Additionally, our findings reveal a strong correlation between the BICePs score and the quality of the structural ensemble, demonstrating an immense utility in this context.

### Variational minimization of the BICePs score to optimize FM parameters and comparison with posterior sampling

Next, we performed variational minimization of the BICePs score to determine optimal FM parameters for our toy model (Figure S2), and compared the results to the posterior sampling approach under the same conditions.

Variational minimization was performed using the Good-Bad model with 4 replicas (for reduced computational cost), where each evaluation of the objective function consisted of running 10,000 MCMC steps. Optimal parameters were determined to be {*A* = 6.31 ± 0.02, *B* = −1.69±0.03, *C* = 1.69 ± 0.01}, averaged over 3 independent runs with very low variance between runs, shown in Figure S6. Regardless of different starting parameters ({*A* = 9, *B* = −1, *C* = 1}, {*A* = 4, *B* = 0, *C* = 3}, {*A* = 0, *B* = 0, *C* = 0}), variational minimization converges to “true” optimal FM parameters. This analysis demonstrated that both the joint posterior sampling approach and variational minimization show nearly equivalent performance when applied to this model.

As a method for forward model optimization, variational minimization of the BICePs score has advantages and disadvantages. This method is particularly advantageous for handling many FM parameters, offering a potential solution to the curse of dimensionality faced by Monte Carlo Markov Chain (MCMC) methods. Additionally, it is easier for users to adapt different forward models, and performs exceptionally well in convex landscapes. When landscapes are non-convex, however, the inverse Hessian may not provide a comprehensive view of the parameter space’s uncertainty; instead, uncertainty estimation could be computed using the variance across multiple BICePs runs starting from different initial parameters. The variational minimization approach also requires careful consideration of disperse starting parameters to ensure global minimization.

The posterior sampling method, which involves sampling the joint posterior distribution of forward model (FM) parameters, has several advantages. One significant benefit is that the posterior distribution provides a direct estimate of the uncertainties of the parameters and their covariance. Compared to variational minimization, this method generally has a faster runtime and is particularly effective in handling non-convex landscapes, allowing for robust parameter estimation even in complex scenarios. However, it is not without drawbacks. As the number of FM parameters increases, the posterior sampling method may encounter the curse of dimensionality, which makes it computationally challenging to explore the parameter space efficiently.

In summary, while both approaches are valuable tools for parameter estimation in parameter and ensemble refinement, each has its strengths and weaknesses. The choice between these methods should be guided by the specific characteristics of the problem at hand, such as the landscape’s convexity and the number of parameters involved.

### Determination of optimal Karplus coefficients for ubiquitin

As an application of our algorithm, we applied BICePs to human ubiquitin to predict Karplus coefficients for six sets of scalar coupling constants: ^3^*J_H^N^H^α^_*, ^3^*J_H^α^C′_*, ^3^*J_H^N^C^β^_*, ^3^*J_H^N^C′_*, ^3^*J_C′C^β^_*, and ^3^*J_C′C′_*. To test the robustness of our algorithm, we conducted a comprehensive evaluation using three different structural ensembles as priors, each derived from distinct computational approaches: (1) 10 conformations from the NMR-refined structural ensemble, 1D3Z^32^, (2) 144 conformations from NMR-restrained simulations, 2NR2^33^, and (3) 25 conformations from the RosettaFold2 (RF2) algorithm.^34^ Further details about these ubiquitin structural ensembles are given in Supporting Information.

We validated the forward model parameters derived from each prior using the BICePs score, *R*^2^ and mean absolute errors (MAE) for forward model predictions. As priors for these validation calculations, we used three independent structural ensembles: 1D3Z and 2NR2 (described above), and a 500-state conformational ensemble derived from a millisecond-long simulation of ubiquitin using CHARMM22*.^35^ Further details about the CHARMM22* ensemble is given in Supporting Information.

To refine the forward model (FM) parameters, we employed full joint posterior distribution sampling. This method was chosen to navigate the non-convex parameter space efficiently, given its relatively low dimensionality (18 FM parameters). Estimates of FM parameters were made by averaging the BICePs results over four Markov Chain Monte Carlo (MCMC) sampling runs, each starting from distinct initial parameters: {*A* = 9, *B* = −1, *C* = 1}, {*A* = 4,*B* = 0,*C* = 3}, {*A* = 0,*B* = 0,*C* = 0}, and {*A* = 6,*B* = −1,*C* = 0}. Flexible residues were excluded from the calculations, consistent with previous studies^2,3^. As a result, a total of 346 *J*-couplings were used in these refinements. We used the Good-Bad model with 32 BICePs replicas, discarding the first 50k steps as burn-in, followed by 50k steps for MCMC sampling. Unlike the parameters derived from 1D3Z and RF2, the Karplus coefficients obtained by using the 2NR2 ensemble required a burn-in of 100k steps to appropriately converge due to a larger number of conformational states. The six sets of refined Karplus coefficients resulting from the 1D3Z, 2NR2 and RF2 ensembles are presented in Table I.

**Figure 3.**
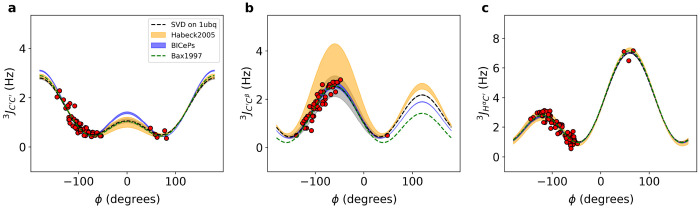
Karplus curves with BICePs-refined Karplus coefficients using the 1d3z ensemble for (a-c) ^3^*J*_*C*′*C*^′^_, ^3^*J_C′C^β^_*, and ^3^*J_H^α^C_*′. For comparison, SVD on 1ubq using experimental scalar coupling constants with *ϕ*-angles derived from the X-ray structure (black dashed line), and red dots correspond to the fitted data points. Additionally, parameterizations from Bax et al. 1997 (green) and parameterization from Habeck et al. 2005 (yellow) were overlaid for comparison. The thickness of the line corresponds to the uncertainty.

Figure 3 compares the Karplus curves derived from BICePs using the 1D3Z ensemble with previously published parameters obtained from NMR refinements, showing subtle differences. Both the marginal posterior distributions of the FM parameters and the Karplus curves for each scalar coupling demonstrate significant congruence with the historical NMR refinement results^3,32^. For all six types of *J*-coupling, see Figure S7.

The predicted parameters have large similarities across structural ensembles. BICePs-predicted coefficients using the 1D3Z ensemble (Figure S8) and predicted coefficients using the RF2 ensemble (Figure S9) are found to strongly overlap. Furthermore, the traces of the FM parameters over time (Figure S10) confirm convergence.

A key advantage of BICePs is its ability to sample the posterior densities of forward model uncertainties, *p*(*σ*) (Figure S11). For certain sets of *J*-coupling constants (e.g., ^3^*J_H^N^H^α^_* and ^3^*J_H^N^C′_*) the marginal posterior distribution of the variance scaling parameter *p*(*ϕ*) has a sampled mean slightly larger than 1.0, indicating that the functional form of the likelihood opted for long tails to account for a few outlier data points deviating from the mean.

#### The BICePs free energy landscape for ^3^J_H^N^C′_ Karplus parameters.

**Figure 4.**
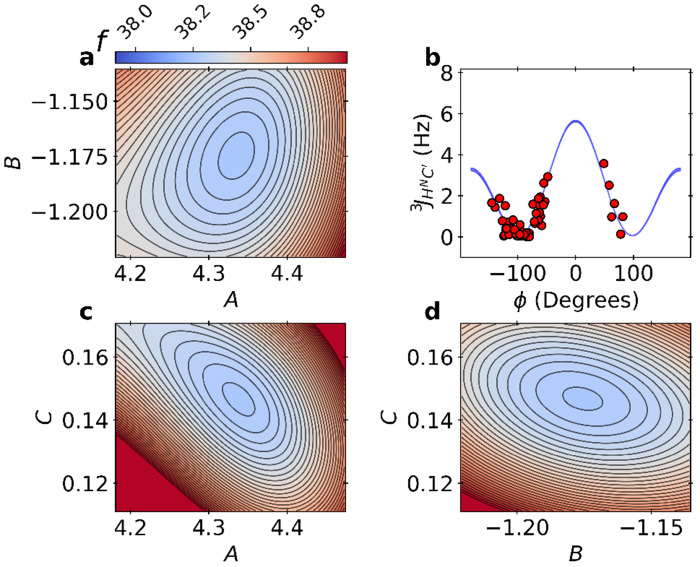
Landscapes of the BICePs score with respect to the predicted Karplus coefficients for ^3^*J_H^N^ C′_*. Panels a, c and d illustrate the energy landscape *f* for pairs of Karplus coefficients when using the 1D3Z structural ensemble during refinement.

In Figure 4, we show the free energy landscape, which is also equivalent to the BICePs score landscape $f_{\xi =0\to 1}$. The Karplus relation for ^3^*J_H^N^C′_* was found to overlap strongly with the results obtained by SVD when using *ϕ* angles from the X-ray crystal structure (Figure S7). Red data points are shown using the experimental *J*-couplings with *ϕ* angles derived from X-ray crystal pose 1UBQ^36^. The joint BICePs score landscape for the six sets of parameters is too complex to visualize. Instead, we constructed a smooth 2-D landscape for each pair of parameters within a set of scalar couplings by training a Gaussian process on the BICePs energy trace using a radial basis function (RBF) kernel with an additive white noise to account for observational uncertainty. The characteristic length scale was bounded within [0.1, 10.0] and the signal variance was set to 1.0 and the noise variance was set to 10^−5^. The landscape matches the computed BICePs scores, and shows minima in the correct locations. All BICePs score landscapes for each of the six sets of Karplus coefficients are illustrated in Figure S12.

**Figure 5.**
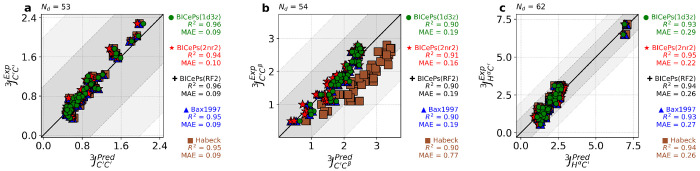
Validation of BICePs-predicted Karplus coefficients perform similarly to Bax1997 and achieve minor improvements over Habeck2005 for scalar coupling predictions for the simulated ensemble of CHARMM22*. Each panel for (a) ^3^*J_H^α^C′_*, (b) ^3^*J_C′C^β^_*, and (c) ^3^*J_C′C′_* shows strong correlations between predictions and experiment. Karplus coefficients derived from BICePs using the 2NR2 ensemble gives the best performance for CHARMM22*. For the remaining sets of *J*-coupling, please see Figure S15.

**Figure 6.**
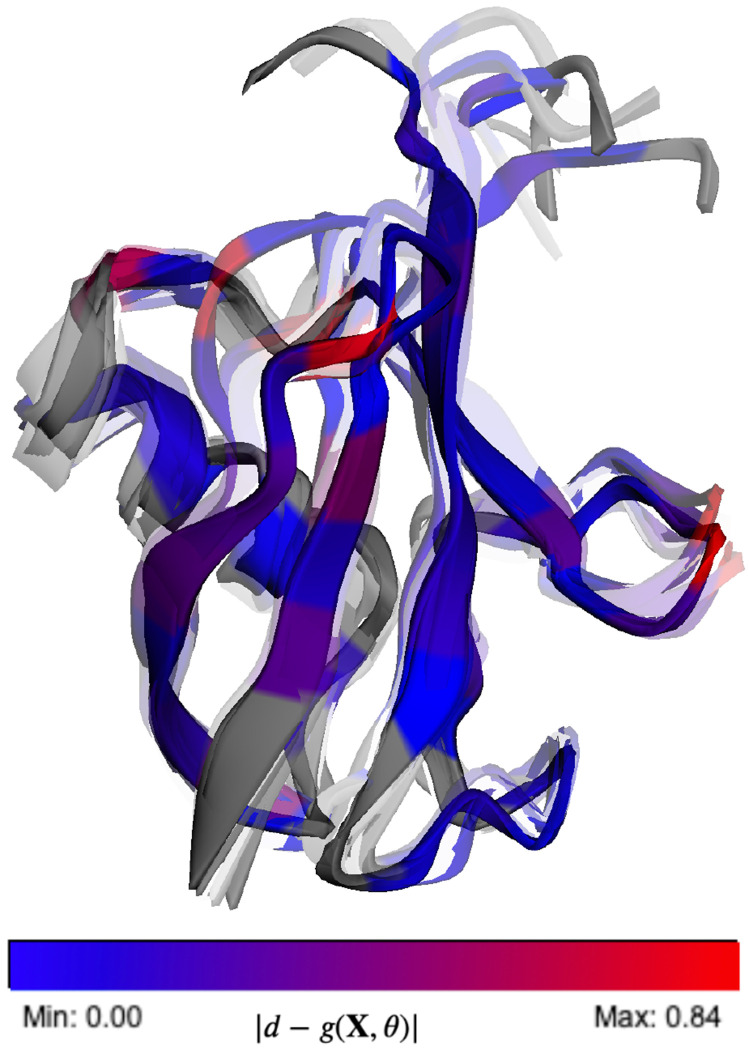
A visualization of the 2NR2 conformational ensemble after joint conformational populations and Karplus parameter optimization using BICePs. Structures (144 in total) with higher transparency have lower populations. Residues are colored by the magnitude of the replica-averaged data deviation from the experiment in ^3^*J_C′C′_*, where the color is normalized from blue (lowest deviation) to red (highest deviation).

To demonstrate the transferability across different generative models and validate our parameters, we evaluated the accuracy of the back-calculated scalar couplings using the different sets of Karplus coefficients. In Figure 5, we illustrate how the various sets of parameters derived from different techniques and different structural ensembles exhibit similar performance metrics. Interestingly, applying BICePs-refined Karplus parameters to an ensemble generated by a molecular dynamics simulation (CHARMM22*),^35^ some parameter sets are revealed to be more transferable than others. The mean absolute error (MAE) and coefficient of determination (*R*^2^) for all six types of scalar couplings across different structural ensembles are shown in Figures S13–S15. On average, the BICePs-refined parameters derived from the 2NR2 ensemble (BICePs(2NR2)) give the lowest MAE between experiment and predictions for the CHARMM22* simulated ensemble, closely followed by BICePs(RF2) parameters, whereas Habeck 2005 has the highest due to known difficulties with ^3^*J_C′C^β^_*. A visualization of the BICePs-reweighted 2NR2 structural ensemble using BICePs(2NR2) parameters is shown in Figure 6 with higher transparency representing lower populations. Residues are colored by the magnitude of the replica-averaged data deviation from the experiment in ^3^*J_C′C′_*, where the color is normalized from blue (lowest deviation) to red (highest deviation).

To quantify which parameters produce the best predictions for ubiquitin, we compute BICePs scores, $f_{\xi =0\to 1}$ for each of the structural ensembles. This score directly relates to the quality of FM parameters and their predictive accuracy at reproducing experimental scalar couplings, integrating over all sources of error. Lower BICePs scores indicate better agreement with experiment. BICePs scores were computed using eleven intermediate ξ-values between 0 and 1, which were optimized using *pylambdaopt*.^29^ The results are shown in Table II. Each row in Table II corresponds to BICePs scores using all six sets of Karplus coefficients used on different structural ensembles. The lowest score is shown in bold. (Note that the BICePs score is an extensive quantity that grows linearly with the number of replicas. For this reason, our results report the *reduced* BICePs score, $f(\theta )/N_{r}$.)

The leftmost column in Table II corresponds to the parameters, where *BICePs(1D3Z)* are the parameters in Table I (set 1), which used 1D3Z ensemble to obtain Karplus coefficients. BICePs score columns, e.g., $f_{\xi =0\to 1}^{1d3z}$ corresponds to BICePs scores evaluated for the 1D3Z ensemble. That is, the superscript corresponds to the structural ensemble used as a validation step. The reported BICePs scores, were averaged over five independent rounds of validation each. BICePs calculations burned for 1k MCMC steps, followed by 50k steps of production-run MCMC sampling.

The BICePs score, $f_{\xi =0\to 1}^{1d3z}=38.14\pm 0.08$(Table II) is equivalent (within error) with the most probable landscape basin *f* = 38.15 ± 0.19 from sampling the energy landscape, computed as an averarge across four chains; an example for one chain is shown in Figure 4. This is additional evidence of the algorithm’s reliability, and corroborates that the results from variational minimization of the BICePs score and full joint posterior sampling are equivalent.

For both the 2NR2 and CHARMM22* structural ensembles, Bax 1997, BICePs(RF2) and BICePs(1D3Z) parameters give very similar BICePs scores, which suggests robust accuracy of FM parameters in reproducing experimental scalar couplings and the transferability of FM parameters across different prior structural ensembles. For the CHARMM22* simulated ensemble, the BICePs(2NR2) parameters give the lowest BICePs score. A partial explanation for this is that the structural ensemble 2NR2 deposited in the Protein Data Bank was generated using the CHARMM22 force field with additional experimental restraints during simulation.^33^

It is difficult to say which of the model parameters are the best for ubiquitin, so we compare the top four: BICePs(RF2), Bax 1997, BICePs(1D3Z), and BICePs(2NR2). The BICePs(2NR2) parameters are objectively better at predicting *J*-couplings from structures of ubiquitin generated from simulations using CHARMM22* force field. Futhermore, our BICePs(RF2) parameters have slightly better transferability across structural ensembles and have a better BICePs score over Bax 1997 parameters. In general, when looking across structural ensembles, the lowest BICePs scores come from the 1D3Z structural ensemble ($f_{\xi =0\to 1}^{1d3z}$) except for BICePs parameters derived from the 2NR2 ensemble (BICePs(2NR2)). This confirms that the 1D3Z structural ensemble gives the strongest agreement with experimental NMR observations.

## IV. DISCUSSION

### Ensembles from deep generative models like RosettaFold2 can be used for parameter refinement.

The booming field of machine learning and artificial intelligence is transforming the field of biomolecular modeling at a swift pace. Recent advancements in generative models, such as AlphaFold^37^, RosettaFold^34^ and others, have heralded a new era in the accurate prediction of structural ensembles. Leveraging the predictive power of these models as structural priors is expected to help refine ensemble predictions when integrated with similar algorithms to BICePs^38^. Here, we have demonstrated that structural ensembles generated from RosettaFold2 (RF2) can be reweighted to better align with experimental measurements, while simultaneously refining Karplus parameters. Validation of these parameters by the BICePs score and other statistics demonstrates improved accuracy across a varity of structural ensembles of ubiquitin.

### Automatic determination of unknown errors.

Our method provides a notable advantage by automatically estimating all potential error sources throughout the ensemble refinement process. This estimation is facilitated through the analysis of posterior distributions, which are instrumental in deriving accurate error assessments for the Karplus coefficients. Consequently, this negates the need for cross-validation techniques commonly used in other approaches^1,39^.

In previous work, we have shown how the BICePs score is a better metric model validation than the traditional χ^2^ test.^6^ Unlike χ^2^, which presupposes a fixed and known error, BICePs dynamically learns the complete posterior distribution of all uncertainty parameters, providing a measure of model quality that integrates over all sources of error. Our work here supports this premise.

### Bayesian ranking of Karplus-type relations

Although here we optimize Karplus relations with a standard functional form, many possible relations can be used to accommodate the diverse characteristics of molecular structures, from rigid to flexible.^40^ The BICePs algorithm can determine coefficients and their uncertainties for any functional form, including those with additional parameters. Although we do pursue this aim in our current work, it is straightforward to use Bayesian model selection to objectively rank empirical models based on their BICePs scores, while automatically accounting for model complexity, thus providing a balance of model accuracy and parsimony.

### Application to Non-Natural and Cyclic Peptides for fine-tuning folding landscapes.

Our framework for forward model optimization has recently been successfully applied to a challenging test case involving a diverse set of non-natural and cyclic *β*-hairpin peptides^41^. In that study, we used BICePs’ forward model optimization approach to refine Karplus parameters for ^3^*J_H^N^H^α^_* predictions directly against experimental NMR data. With an improved Karplus relation, BICePs reshaped the folding landscapes to better agree with NMR data, enabling more accurate predictions of folding stability for each peptide. This work highlights the resolution enhancement gained by the improved forward model predictions and population reweighting using BICePs, enabling the detection of subtle changes in folding stability—such as those introduced by a sidechain hydrogen- or halogen-bonding group, which alters stability by no more than 2 kJ/mol—offering a reliable pathway for designing foldable non-natural and cyclic peptides.

### The BICePs score can be used as a loss function to train neural networks and other differentiable forward models.

A central challenge in physical sciences is integrating domain knowledge with data-driven models. Traditional machine learning often lacks physical consistency or interpretability, while purely physics-based models may miss subtle experimental trends. One way to bridge this gap is to use the BICePs score—a free energy-based Bayesian loss—to train machine learning forward models using ensemble-averaged experimental data. This allows one to parameterize neural networks or other differentiable models in a physically grounded, uncertainty-aware fashion. In this way, BICePs provides a general-purpose learning framework for constructing interpretable, probabilistically-justified forward models for complex observables. This capability may be especially valuable in contexts where observables are too complex to be modeled analytically—such as in chemical shift prediction or other nonlinear structure–observable relationships^13,42,43^.

To demonstrate the viability of using BICePs for neural network training, we constructed a toy model for *J*-coupling prediction. In this model, the backbone dihedral angle *ϕ* was the sole input variable. To appropriately encode the periodicity of angular data, each *ϕ*-angle was embedded into two dimensions via a sine–cosine transformation:

(22) $$x=\left[\begin{array}{c} cos(\phi )\\ sin(\phi ) \end{array}\right]\in {\mathbb{R}}^{2}$$

This representation ensures continuity across the periodic boundary at ±*π* and provides a smooth input feature space for learning.

A fully connected feedforward neural network was constructed using this 2D input, consisting of one hidden layer with 200 neurons and Gaussian Error Linear Unit (GELU) activation functions. The final output was a scalar prediction corresponding to the *J*-coupling value. To ensure stable training dynamics compatible with GELU, the weights were initialized using Le-Cun normal initialization^44^.

The network was trained using ADAM optimization for 2000 epochs with a learning rate of 10^−3^, using the Good-Bad likelihood model with the BICePs score as the loss function. The toy dataset consisted of 5 conformational states, each with 60 synthetic *J*-coupling observables using the Karplus relation *J*(*ϕ*) = *A*cos^2^(*ϕ*) + *B*cos(*ϕ*) + *C*, with the ground truth coefficients *A* = 6.51, *B* = −1.76, and *C* = 1.60. Our training dataset consisted of 300 *ϕ*-angles as NN inputs.

First, we test the performance without adding random or systematic noise to the synthetic experimental J-coupling observables. Figure S16a shows the convergence behavior of the BICePs score across five independent training runs, where a plateau is reached around step 1500. Figure S16b shows the Karplus curves predicted by the five trained networks, along with their mean and standard deviation (blue curve). For comparison, we also include the result of singular value decomposition (SVD) applied directly to the Karplus form under the assumption that backbone *ϕ* angles are known precisely, as would be the case with a high-resolution crystal structure. The parameters from SVD were determined to be *A* = 6.47 ± 0.002, *B* = −1.75 ± 0.001, and *C* = 1.62 ± 0.001.

Next, we evaluate the performance when adding random or systematic noise to the synthetic experimental *J*-coupling observables (σ_data_ = 0.79 Hz). Figure S17a–b shows the convergence behavior of the BICePs score and gradient norm across five independent training runs, where convergence begins around 1000 epochs. The BICePs score was determined to be *f*_*ξ*=0→1_ = 62.29 nats. Figure S17c shows the Karplus curves predicted by the five trained networks, along with their mean and standard deviation (blue curve). Comparison against SVD illustrates that BICePs, when used to train neural networks from conformational ensembles alone, recovers predictive performance comparable to analytical fitting based on ground-truth dihedral angles, even in the presence of random and systematic errors.

Convergence of the BICePs score is typically accompanied by a reduction in the posterior uncertainty parameter σ, which sharpens the likelihood landscape and amplifies gradients with respect to model parameters. Specifically, the derivative of the BICePs score with respect to a parameter *θ* contains a term proportional to 1/σ^2^ (see equations 12 and 13), meaning that as **σ** → 0 the sensitivity of the loss to prediction errors increases.

During training of the NN-based forward model, the posterior sampling of **σ** plays a critical role in regularization. If **σ** were held fixed during training, the model would be limited in its ability to adjust the sharpness of the likelihood surface and could fail to reach an optimal fit to the data. By treating **σ** as a nuisance parameter and marginalizing over it within the BICePs framework, the model adaptively tunes the effective strength of the loss function, yielding an implicit form of regularization. This dynamic adjustment helps stabilize training and ensures that neural network parameters are optimized in a manner consistent with both the experimental data and the underlying physical ensemble.

While this toy model serves as a useful proof-of-concept, the broader applicability of our framework is in modeling more complex observables such as NMR chemical shifts. These observables are typically nonlinear and high-dimensional functions of atomic coordinates, making them difficult to model analytically. In contrast, neural networks trained using BICePs can serve as flexible surrogates for such forward models. Importantly, this framework supports the simultaneous training of multiple neural networks, each targeting a distinct observable—for example, one network may predict ^3^*J*_HN–Hα_ couplings, another ^3^*J_C_*_′˘C′_ couplings, and others may be used for predicting chemical shifts. In the case of backbone chemical shift prediction, a separate neural network is generally constructed for each nucleus type (e.g., C*β*, ^1^H*α*, and ^1^HN), enabling specialized learning tailored to the distinct structural dependencies of each observable. All networks can be trained jointly using a shared conformational ensemble, with BICePs enforcing consistency with the corresponding experimental data.

Models have limited predictive reliability in regions lacking sufficient training data. A central advantage of our framework lies in its ability to incorporate physically relevant information through the choice of input features or explicit regularization. In our example using the toy model system, we employed the backbone dihedral angle *ϕ* as the sole input feature (projected to the unit circle to maintain periodicity). However, neural network inputs can encompass a broader range of features for complex observables such as chemical shifts, including quantum calculations, partial charges, descriptors of local atomic environments, interatomic distances, hydrogen bonding patterns, solvent accessibility, or torsional strain energies. These diverse features encode crucial structural and electronic context and can be precomputed from molecular simulations or quantum calculations before training.

We did not impose physical constraints or priors when training our model to predict *J*-coupling constants. Users might explicitly incorporate such physical priors or constraints through architectural decisions, regularization terms, or physically-informed input features, especially critical when dealing with sparse or unevenly distributed training data. For instance, in our experiments, neural networks employing Rectified Linear Unit (ReLU) activations struggled to accurately predict *J*-couplings at backbone dihedral angles greater than +90°. In contrast, GELU activations provided smoother and more reliable extrapolation in these underrepresented regions. Thus, careful selection of activation functions and comprehensive input feature representations significantly enhances predictive accuracy across the full conformational landscape.

## V. CONCLUSION

In the quest for accurate forward model predictions, specifically for *J*-coupling, researchers often navigate the vast literature seeking Karplus parameters that align with their specific systems, occasionally settling for less-than-ideal solutions. Our work demonstrates that BICePs can be used as a robust tool for determining forward model (FM) parameters by sampling over the full posterior distribution of their values and uncertainties.

We used a toy model of protein dihedral angles to demonstrate that posterior sampling and variational minimization of the BICePs score are valid and robust approaches for FM parameter refinement. Using structural ensembles and experimental data for ubiquitin, we applied BICePs with posterior sampling to optimize six different sets of Karplus coefficients using different types of *J*-coupling measurements, while effectively addressing both random and systematic errors.

From these results, one can see how BICePs can be applied more generally to optimize a wide variety of forward models, including those represented by neural networks. By treating the BICePs score as a physically grounded loss function, our framework enables the training of data-driven forward models that are consistent with ensemble-averaged experimental observables and incorporate principled uncertainty quantification. These advances not only contribute to the refinement of molecular simulations but also provide a scalable and extensible machine learning approach for physical sciences, with applications in structural dynamics, model validation, and the construction of predictive, interpretable models across a broad spectrum of data regimes.

## Supplementary Material

1

## Figures and Tables

**Table I.** Coefficients for the Karplus relation ^3^*J*(*ϕ*) = *A*cos^2^(*ϕ*+*ϕ*_0_)+*B*cos(*ϕ*+*ϕ*_0_)+*C*, determined by BICePs sampling the joint posterior of FM parameters.

		$\phi_{0}$	A (Hz)	B (Hz)	C (Hz)
^3^ *J_C′C_*	1	0°	1.71 ± 0.02	−0.85 ± 0.01	0.54 ± 0.00
	2	0°	1.30 ± 0.03	−0.91 ± 0.01	0.62 ± 0.01
	3	0°	1.62 ± 0.03	−0.87 ± 0.01	0.63 ± 0.01
^3^ *J_C′C^β^_*	1	60°	1.83 ± 0.04	0.34 ± 0.05	0.41 ± 0.02
	2	60°	2.20 ± 0.04	0.34 ± 0.04	0.04 ± 0.02
	3	60°	1.81 ± 0.04	0.38 ± 0.04	0.31 ± 0.02
^3^ *J_H^α^C′_*	1	120°	3.64 ± 0.02	−2.14 ± 0.02	1.27 ± 0.02
	2	120°	4.10 ± 0.03	−2.00 ± 0.02	0.95 ± 0.02
	3	120°	3.78 ± 0.02	−2.12 ± 0.02	1.21 ± 0.02
^3^ *J_H^N^C′_*	1	180°	4.33 ± 0.04	−1.17 ± 0.01	0.14 ± 0.01
	2	180°	4.60 ± 0.12	−0.57 ± 0.03	−0.10 ± 0.01
	3	180°	4.57 ± 0.09	−1.20 ± 0.03	0.13 ± 0.01
^3^ *J_H^N^C^β^_*	1	60°	2.72 ± 0.03	−0.35 ± 0.03	0.12 ± 0.01
	2	60°	3.00 ± 0.04	−0.26 ± 0.03	−0.28 ± 0.02
	3	60°	2.52 ± 0.03	−0.03 ± 0.02	−0.09 ± 0.02
^3^ *J_H^N^H^α^_*	1	−60°	7.11 ± 0.05	−1.38 ± 0.03	1.43 ± 0.04
	2	−60°	7.50 ± 0.07	−1.50 ± 0.02	1.50 ± 0.06
	3	−60°	6.97 ± 0.07	−1.49 ± 0.04	1.63 ± 0.05

1 1D3Z as the structural ensemble

2 2NR2 as the structural ensemble

3 RosettaFold2 (RF2) as the structural ensemble

**Table II.** BICePs scores (32 replicas), *f* for each structural ensemble over all sets of parameters, averaged over five independent rounds of validation each.

Parameters	$f_{\xi =0\to 1}^{1d3z}$	$f_{\xi =0\to 1}^{2nr2}$	$f_{\xi =0\to 1}^{\text{CHARMM22*}}$
Bax 1997^2,32^	61.12 ± 0.08	132.49 ± 0.09	99.27 ± 1.91
Habeck 2005^3^	135.66 ± 0.08	199.32 ± 0.24	165.64 ± 0.47
BICePs(1D3Z)	**38.14** ± **0.08**	141.69 ± 0.16	105.00 ± 0.74
BICePs(2NR2)	118.42 ± 0.14	**113.15** ± **1.34**	**76.27** ± **0.66**
BICePs(RF2)	68.07 ± 0.60	129.42 ± 0.15	88.04 ± 0.21

## Data Availability

The BICePs algorithm is freely available at github.com/vvoelz/biceps. All scripts, Jupyter notebook examples and analysis can be found here. Detailed documentation and tutorials can be found here: biceps.readthedocs.io. For any issues or questions, please submit the request on GitHub.
