# Supplementary material for: Automatic Forward Model Parameterization with Bayesian Inference of Conformational Populations
Source: ArXiv. 2025 Jun 18:arXiv:2405.18532v2. Originally published 2024 May 28. Preprint. [Version 2] (PMC11160882)
Supplement: 1 [file NIHPP2405.18532v2-supplement-1.pdf]

Supplemental Information

Automatic Forward Model Parameterization with Bayesian Inference of Conformational Populations

Robert M. Raddi, Tim Marshall and Vincent A. Voelz

APPENDIX A: SECOND PARTIAL DERIVATIVES OF THE BICEPS SCORE WITH RESPECT TO FORWARD MODEL PARAMETERS.

The second partial derivatives of the BICePs score with respect to the FM parameters can be used for second-order optimization methods and uncertainty quantification. For simplicity, we refrain from showing the complicated second derivatives for the Good-Bad model in the general case. In the case when when $\varphi = 1$, however, the second partial derivatives of the BICePs energy function with respect to parameters θ_a and θ_b are:

$$\frac{\partial^2 u}{\partial\theta_m\partial\theta_n} = N\left[\sum_{j=1}^{N_d} -\frac{\partial^2 g_j(X,\theta)}{\partial\theta_m\partial\theta_n}\frac{(d_j - g_j(\mathbf{X},\theta))}{\sigma_j^2} + \frac{\partial g_j(X,\theta)}{\partial\theta_m}\cdot\frac{\partial g_j(X,\theta)}{\partial\theta_n}\frac{1}{\sigma_j{}^2}\right]. \tag{S1}$$

When $m = n$, the second partial derivative of the energy is just:

$$\frac{\partial^2 u}{\partial\theta_m^2} = N\left[\sum_{j=1}^{N_d}\left(-\frac{\partial^2 g_j(X,\theta)}{\partial\theta_m^2}\frac{(d_j - g_j(\mathbf{X},\theta))}{\sigma_j^2} + \left(\frac{\partial g_j(X,\theta)}{\partial\theta_m}\right)^2\frac{1}{\sigma_j{}^2}\right)\right]. \tag{S2}$$

The second partial derivatives of the BICePs score with respect to parameters θ_m and θ_n are:

$$\frac{\partial^2 f(\theta)}{\partial\theta_m\partial\theta_n} = \left\langle\frac{\partial^2 u}{\partial\theta_m\partial\theta_n}\right\rangle - \left(\left\langle\frac{\partial u}{\partial\theta_m}\cdot\frac{\partial u}{\partial\theta_n}\right\rangle - \left\langle\frac{\partial u}{\partial\theta_m}\cdot\frac{\partial u}{\partial\theta_n}\right\rangle'\right), \tag{S3}$$

where the notation $\langle\cdot\rangle'$ denotes the ensemble average with respect to $\left(\frac{1}{Z}\exp(-u)\right)^2$. The first term on the right is the ensemble-average second derivative of the energy function u with respect to parameters θ_m and θ_n given in equation S2 (when $\varphi = 1$).

When $m = n$, the second partial derivative of the BICePs score reduces to the difference between the ensemble-averaged second derivative of the energy u and the variance of its first partial derivative:

$$\frac{\partial^2 f}{\partial\theta^2} = \left\langle\frac{\partial^2 u}{\partial\theta^2}\right\rangle - \left(\left\langle\left(\frac{\partial u}{\partial\theta}\right)^2\right\rangle - \left\langle\frac{\partial u}{\partial\theta}\right\rangle^2\right) \tag{S4}$$

In practice, this calculation is performed using the MBAR free energy estimator for the BICePs score and it's derivatives, by sampling at several intermediates $\xi = 0 \to 1$, which enables accurate estimates of all quantities.

METHODS

Structural ensembles of human ubiquitin

1D3Z This structural ensemble consists of the 10 conformations deposited in the Protein Data Bank (PDB: 1D3Z) from the NMR structural refinement performed by Cornilescu et al.[32]. The ensemble was calculated by the program X-PLOR using 2727 NOE distance restraints and 98 dihedral angle restraints derived from homo- and heteronuclear J couplings.

2NR2 The 2NR2 structural ensemble (144 conformations) was taken from Richter et al. (PDB: 2NR2), where the refinement was performed using the MUMO (minimal under-restraining minimal over-restraining) method[33]. In this approach, simulations were started from the X-ray crystal pose[36] in the presence of replica-averaged restraints to NOEs distances and S^2 Lipari–Szabo order parameters. Simulations used TIP3P solvent and the CHARM22 force field, with an augmented potential energy function $E_{\text{total}} = E_{\text{CHARMM22}} + E_{\text{restraints}}$. Scalar couplings were not used during the refinement, but were only used as a validation metric.

RosettaFold2 (RF2) The RF2 structural ensemble was generated using RosettaFold2[34] made available through a Colabfold notebook.[46] Default parameters were used, and 25 conformations were generated.

*CHARMM22** The CHARMM22* structural ensemble was derived from a Markov State Model (MSMs) we constructed from a one-millisecond simulation of ubiquitin's native state at 300 K from Piana et al.[35]. The PyEMMA Python package[47] was used to determine appropriate backbone featurizations using the Variational Approach for Markov Processes (VAMP) scoring function VAMP-2[48]. Based on the VAMP-2 scores, inverse distances were selected as features, while torsions were excluded due to lower VAMP-2 scores and their minimal contribution when paired with inverse distances. When comparing distances and inverse distances, the similarity of the average scores and relative standard deviations show that both featurizations are adequate, and inverse distances was selected purely on the higher average scores. Time-lagged independent component analysis (tICA) followed by k-means clustering was used to partition the trajectory data into 500 discrete conformational states for MSM construction[49,50]. For the BICePs calculation, each of the 500 states was given equal statistical weight to enforce the uniform prior $p(X)$.

Details of parameters used in posterior sampling

Uncertainty parameters σ were sampled on a grid of logarithmically-spaced values between 0.001 to 100, to enforce the Jeffrey's prior. Each grid value in the list was a factor of 1.02 larger than the next: `[1.00e-03, 1.02e-03, 1.04e-03, 1.06e-03, ... 9.72e+01, 9.92e+01]`, resulting in a list of 582 values. For the Good-Bad model, sampling of the extra nuisance parameter, φ took place on a grid from 1 to 100 with 1000 equally-spaced points.

Details of ξ optimization

Optimization was performed for a maximum of 2M steps with a tolerance of 10^{-7} and $\alpha = 10^{-5}$. With increasing amounts of data restraint energy, the optimization problem becomes more complicated and more iterations are required to converge. In the case of insuffcent iterations, the ξ-optimization might return negative ξ-values, which is incorrect and not physical. Initially, we start with 11 ξ-values `{0.0, 0.1, ... 0.9, 1.0}` and with shift to lower values of ξ after optimization e.g., `{0.0, 0.02, 0.04, 0.07, 0.12, 0.26, 0.40, 0.60, 0.90, 0.97, 1.0}`.

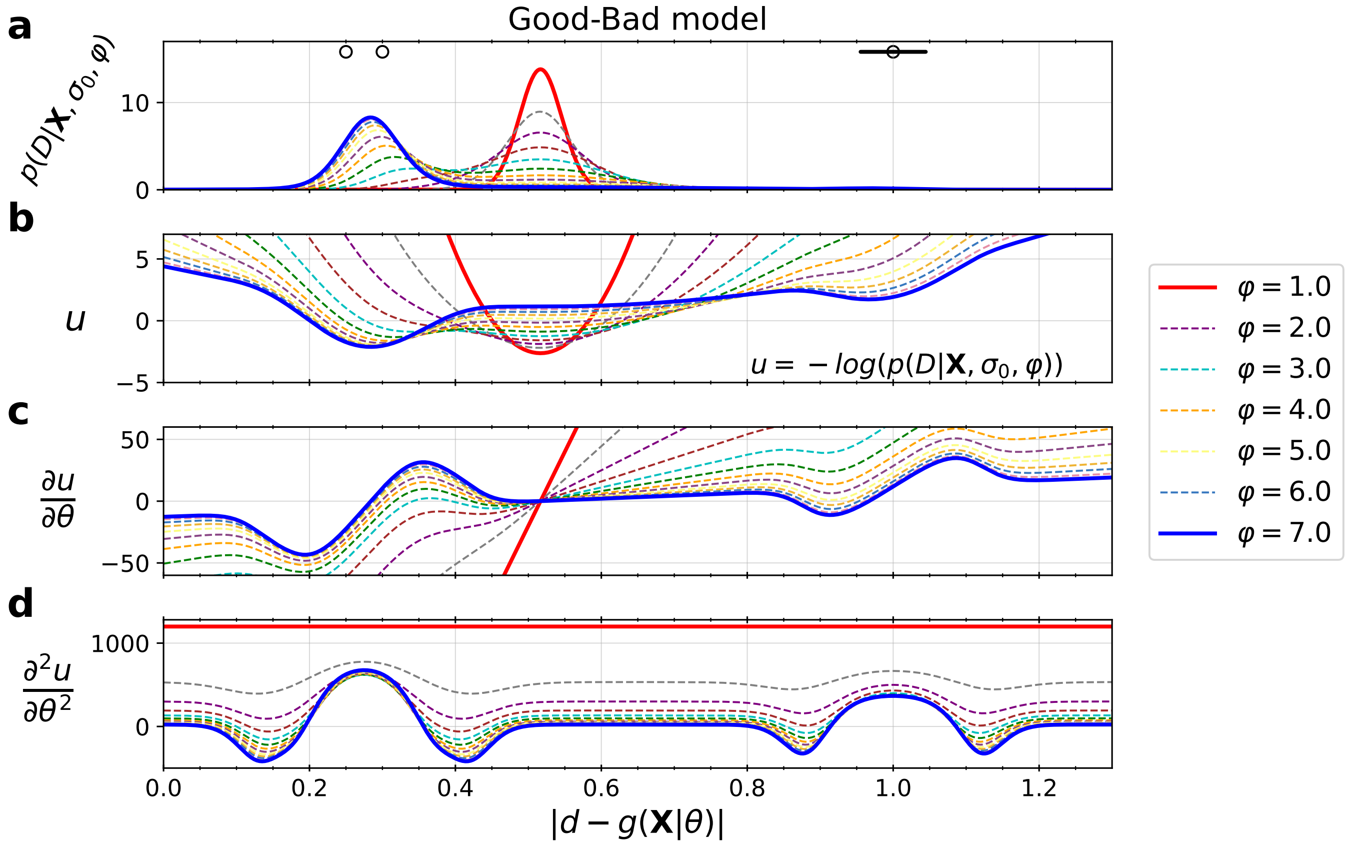

Figure S1. The Good-Bad model properly detects outliers. The probability density function (a) computed as $p(D|\mathbf{X}, \sigma_0, \varphi) = \prod_j^{N_j} p(d_j|\mathbf{X}, \sigma_0, \varphi)$ and energy landscape (b) of the marginal likelihood for the Good-Bad model with respect to the replica-averaged forward model data $f(\mathbf{X})$ using multiple data points. Shown here, are three data points, two good data points $\{0.25, 0.3\}$ and one outlier $\{1.0\}$. The Good-Bad model ($\varphi = 7.0$) is centered about the mean of the two good data points, demonstrating that this model can distinguish the good and bad data. The standard Gaussian likelihood ($\varphi = 1$) is centered about the mean of all three data points. The colored curves are different values of nuisance parameter φ. Subplots (a) and (b) show how the Good-Bad model when $\varphi = 1$ is equivalent to the Gaussian likelihood and harmonic potential energy function. Subplots (c) and (d) are the first and second derivatives of the potential energy curves shown in subplot (b).

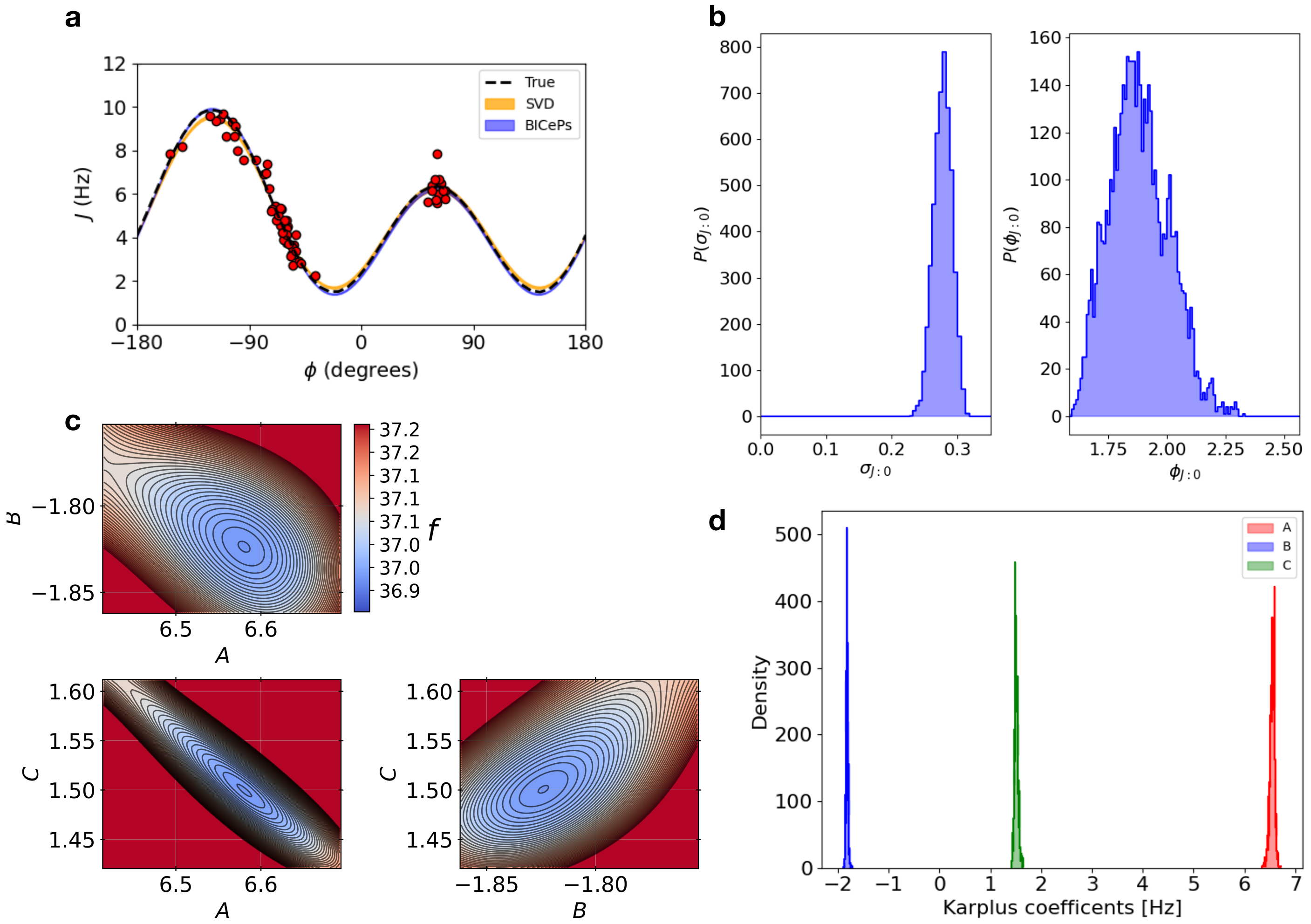

Figure S2. **BICePs predicted forward model parameters in the presence of random and systematic error** ($\sigma_{data} = 0.471$ **Hz) for a toy model system.** (a) Karplus curves predicted for SVD (orange) and BICePs (blue), where the "true" (black dashed line) parameters were set to be $\{A = 6.51, B = -1.76, C = 1.6\}$. The extracted parameters from the SVD fitting were found to be $\{A = 6.11 \pm 0.06, B = -1.63 \pm 0.04, C = 1.80 \pm 0.04\}$ and the BICePs was $\{A = 6.6 \pm 0.037, B = -1.8 \pm 0.016, C = 1.5 \pm 0.027\}$, averaged over three independent chains. The uncertainty is represented by the thickness of the curves. For the BICePs calculation, we used the Good-Bad likelihood model with 32 replicas and burned for 20k steps, followed by 50k steps of additional sampling. Red dots correspond to the syntetic experimental J-coupling data points. (b) The marginal posterior distribution of uncertainty $p(\sigma_J)$. The maximum a posteriori was determined to be $\hat{\sigma}_J = 0.272$ Hz, and the *a posteriori* variance scaling parameter $\hat{\varphi}_J = 1.98$. (c) Landscapes of the BICePs score, f for pairs of Karplus coefficients. (d) The marginal posterior distribution of Karplus coefficients.

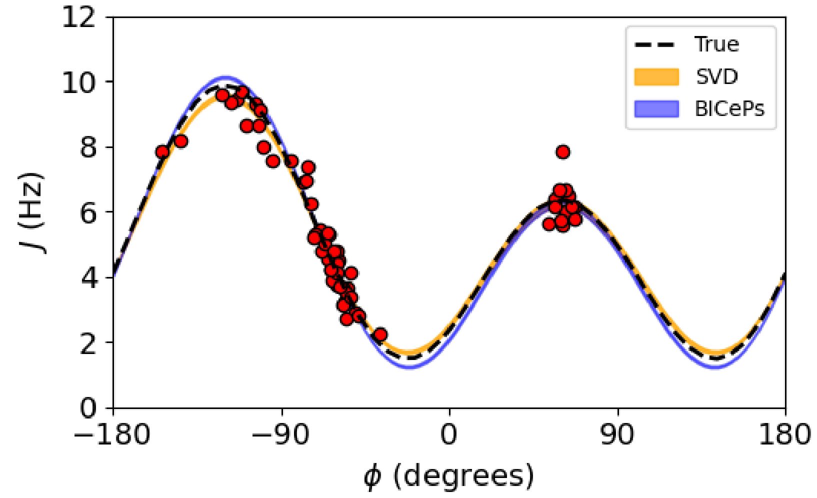

Figure S3. **The Student's model gives similar performance to the Good-Bad model.** Karplus coefficients predicted using the Student's likeliood model ($\{A = 6.8 \pm 0.033, B = -1.9 \pm 0.025, C = 1.4 \pm 0.033\}$) are compared against SVD when faced with random and systematic error (σ_{data} = 0.471 Hz). The "True" parameters were set to be $\{A = 6.51, B = -1.76, C = 1.6\}$, the same as Figure S2.

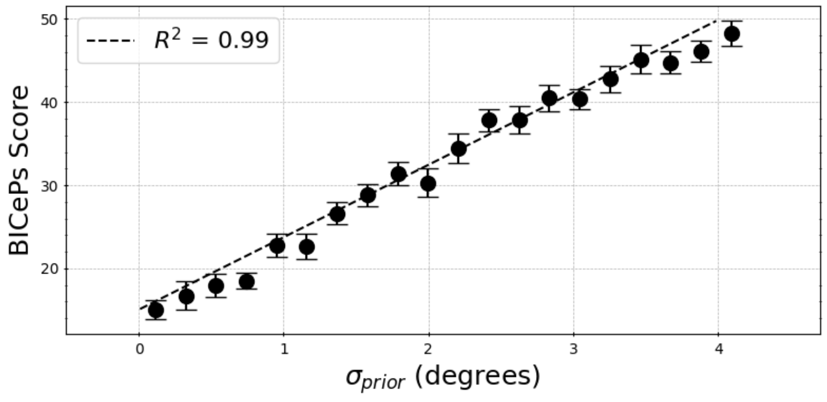

Figure S4. **The BICePs score is a measure of structural ensemble quality.** Using the same toy model system described in the main text. We vary the quality of the prior structural ensemble (σ_{prior}) by perturbing the "true" ϕ angles of the structural ensemble. In these experiments, we induced over 1,000 random perturbations to the prior structural ensemble, and calculated BICePs scores for each. Error bars represent the standard deviation from the mean. The top panel shows the relationship between the BICePs score and the amount of error added to the structural ensemble. Each data point is an average across of 100 BICePs calculations. In these calculations, we used the Good-Bad likelihood model with 32 replicas.

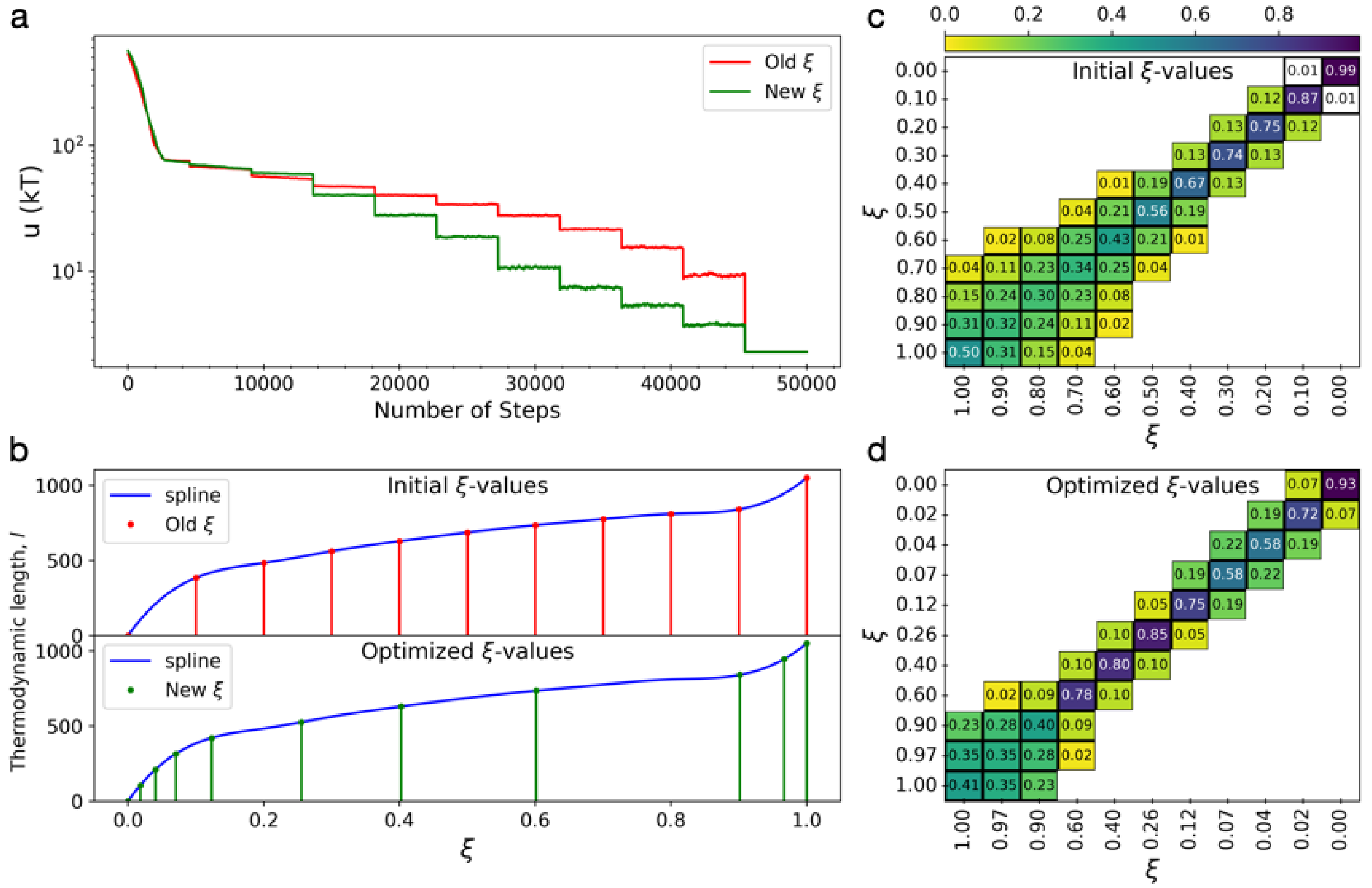

Figure S5. **Optimization of thermodynamic intermediates ξ_k used to calculate the BICePs score, $f_{\xi=0\to1}$.** In this scenario, sampling of the BICePs energy function $u = -\log p(\mathbf{X}, \sigma | D, \theta)$ is performed for a series of intermediates ξ_k progressively from $\xi = 1$ to $\xi = 0$ (a). The samples are used to optimize a new set of ξ_k spaced uniformly in thermodynamic length (b). While the thermodynamic overlap matrix for the unoptimized ξ_k show poor overlap in the region of low ξ values (c), the overlap matrix for the optimized ξ_k shows good overlap for all neighboring intermediates (d). Reproduced from Ref.[29] with permission.

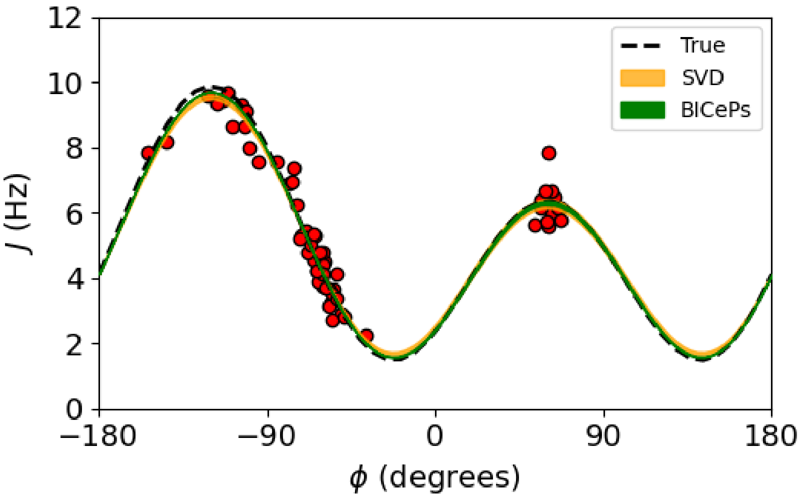

Figure S6. **Variational minimization of the BICePs score can be used to optimize forward model parameters.** By variational minimzation of the BICePs score, f optimization traces converge to the "true" parameters. In these calculations, ξ-values were optimized prior to running the parameter refinement. We used the Good-Bad model with 4 replicas to minimize computational cost. Karplus coefficients predicted using the Good-Bad likeliood model ($\{6.31 \pm 0.02, -1.69 \pm 0.03, 1.69 \pm 0.01\}$) are compared against SVD when faced with random and systematic error ($\sigma_{data} = 0.471$ Hz). The "True" parameters were set to be $\{A = 6.51, B = -1.76, C = 1.6\}$, the same as Figure S2.

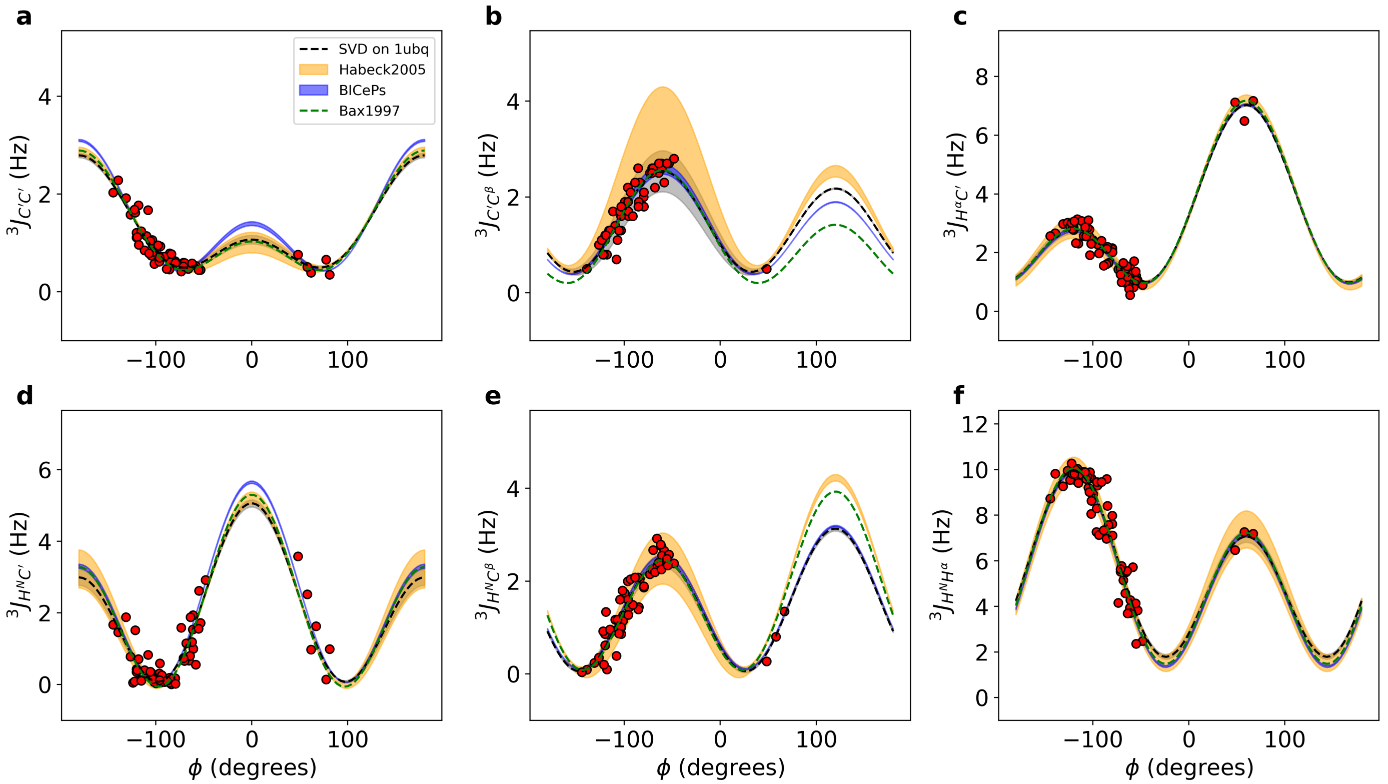

Figure S7. Karplus curves with BICePs refined Karplus coefficients using the 1d3z ensemble for (a-f) $^3J_{C'C'}$, $^3J_{C'C\beta}$, $^3J_{H^\alpha C'}$, $^3J_{H^N C'}$, $^3J_{H^N C\beta}$, $^3J_{H^N H^\alpha}$. BICePs calculations were run using four chains with 32 replicas each, where we burned 50k steps, then sampled for another 50k MCMC steps. For comparison, SVD on 1ubq using experimental scalar coupling constants with ϕ-angles derived from the X-ray structure (black dashed line) and red dots correspond to the fitted data points. Additionally, parameterizations from Bax et al. 1997 (green), and parameterization from Habeck et al. 2005 (yellow) were overlaid for comparison. The thickness of the line corresponds to the uncertainty.

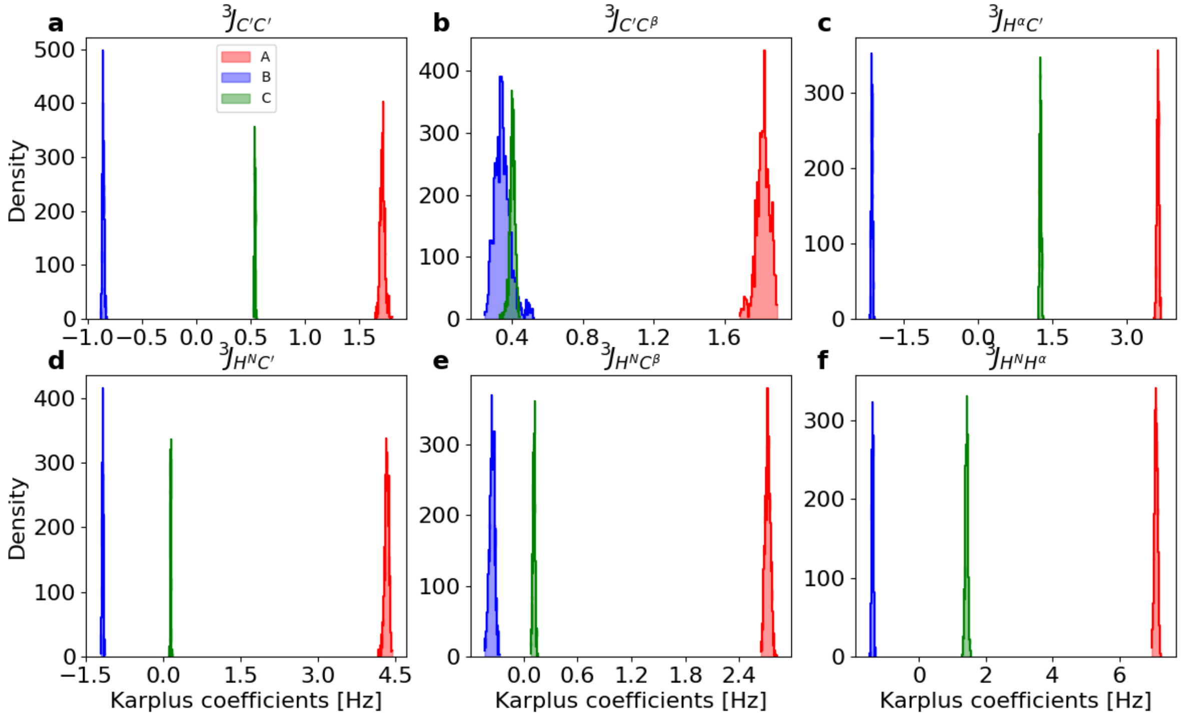

Figure S8. **Sampling the joint posterior distributions of six sets of Karplus coefficients using the Good-Bad model on the 1d3z ensemble.** BICePs calculations were run using four chains with 32 replicas each, where we burned 50k steps, then sampled for another 50k MCMC steps. These marginal posterior distributions shown here are from a randomly selected chain.

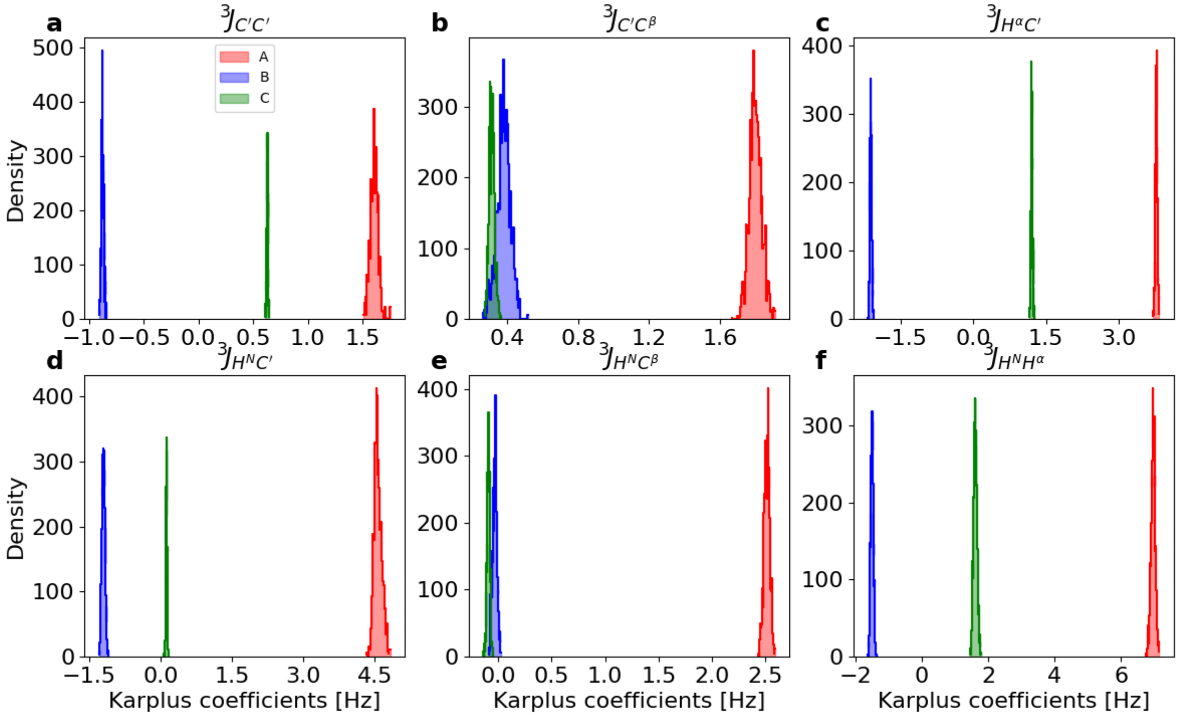

Figure S9. **Sampling the joint posterior distributions of six sets of Karplus coefficients using the Good-Bad model on the RosettaFold2 (RF2) ensemble.** BICePs calculations were run using four chains with 32 replicas each, where we burned 50k steps, then sampled for another 50k MCMC steps. Compare with Figure S8 to see similarities.

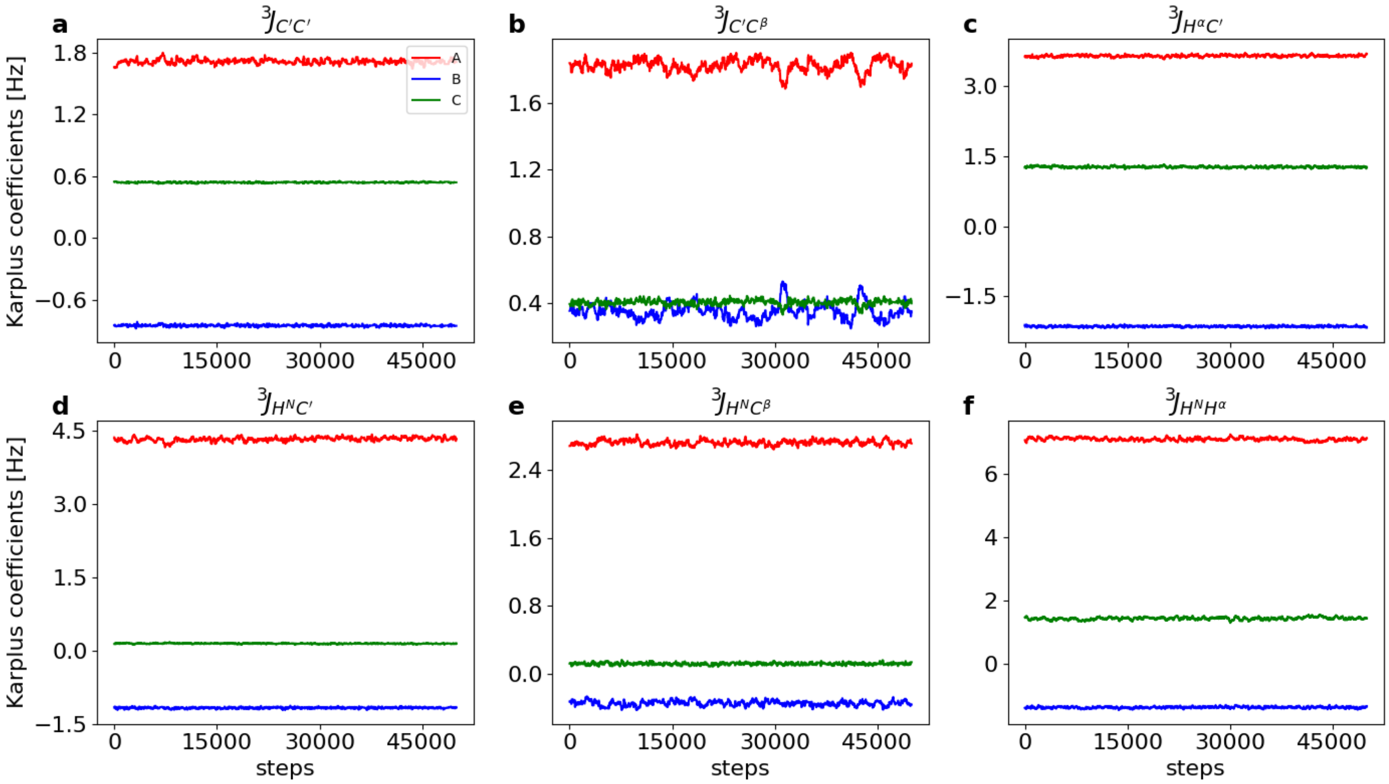

Figure S10. Traces of sampled Karplus coefficients for the 1d3z ensemble over 50k steps of MCMC, post-burn. BICePs calculations used the Good-Bad model with 32 replicas. Traces display low variance with no jumps, which demonstrates converged samples

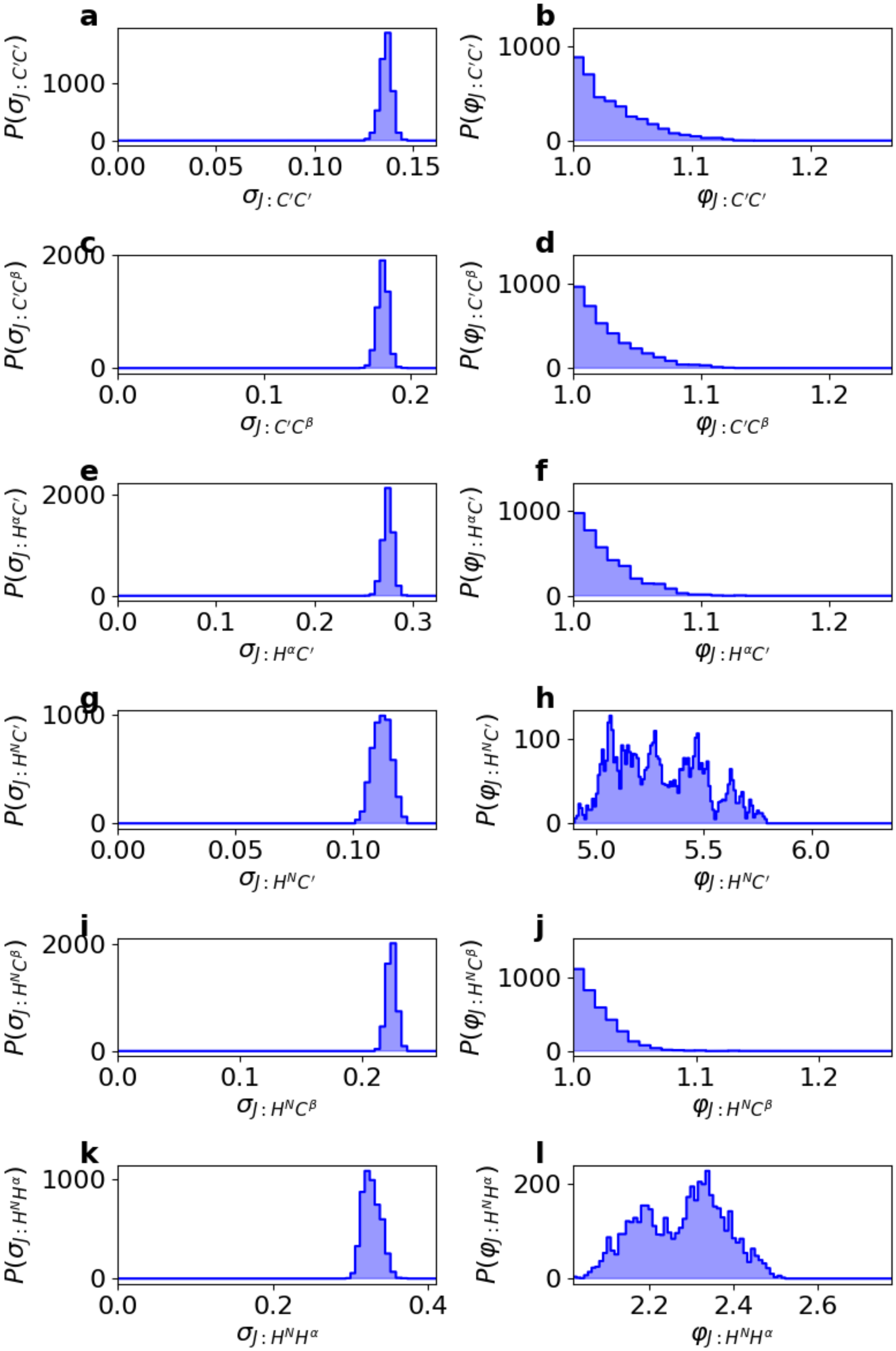

Figure S11. The marginal posterior distribution of σ_J, the uncertainty parameters for each set of J-coupling in the 1d3z ensemble. Densities are a result of posterior sampling of FM parameters during ensemble refinement using the Good-Bad model with 32 replicas. The marginal posterior distributions of the variance scaling parameter φ has a sampled mean slightly larger than 1.0 for particular sets of J-coupling, indicating that the functional form of the likelihood opted for long tails to account for a few outlier data points deviating from the mean.

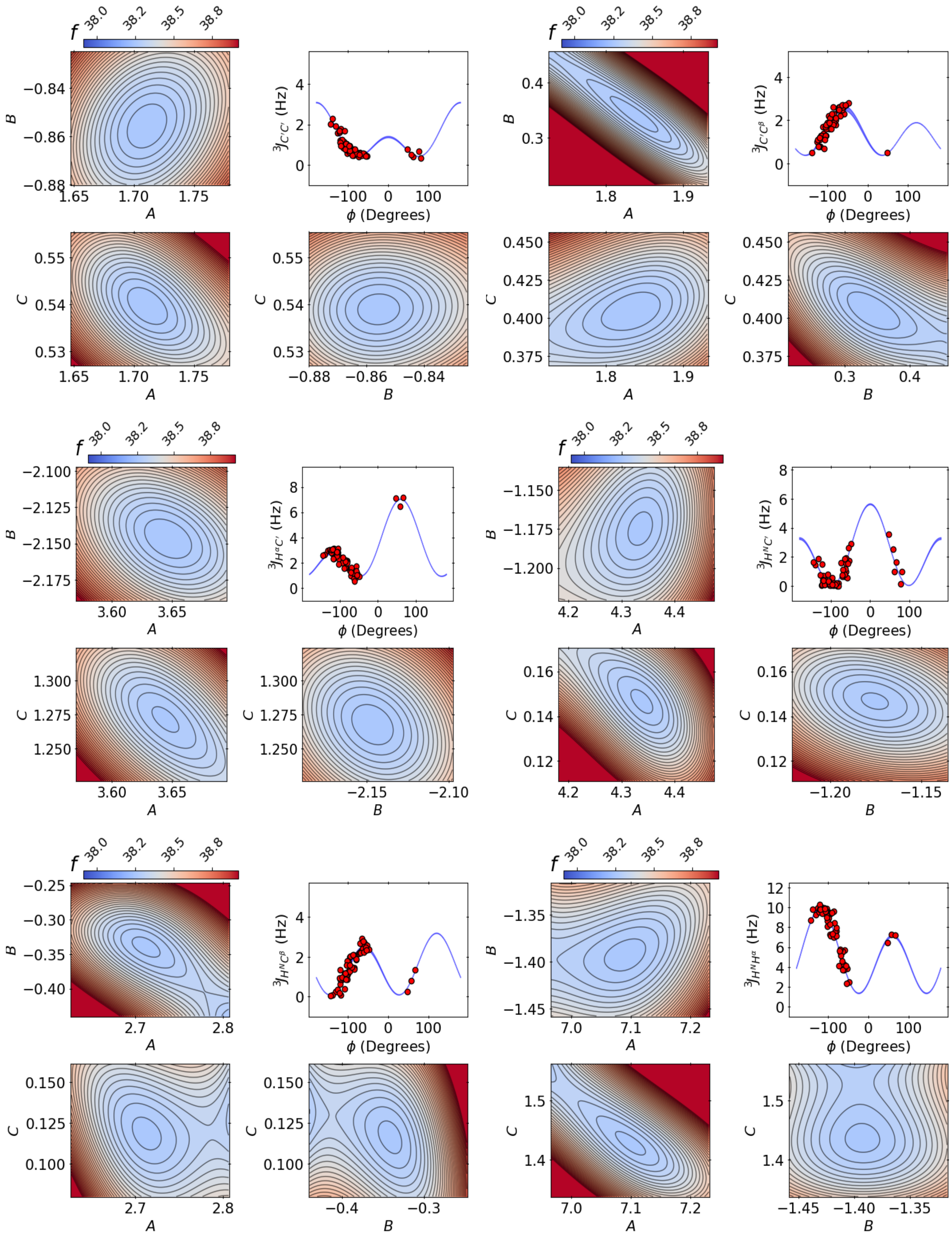

Figure S12. BICePs score landscapes of FM parameters on the 1d3z ensemble, unveiled during ensemble refinement. BICePs calcualtions used the Good-Bad model with 32 replicas. Each set of $\{A, B, C\}$ was included in the joint posterior of FM parameters.

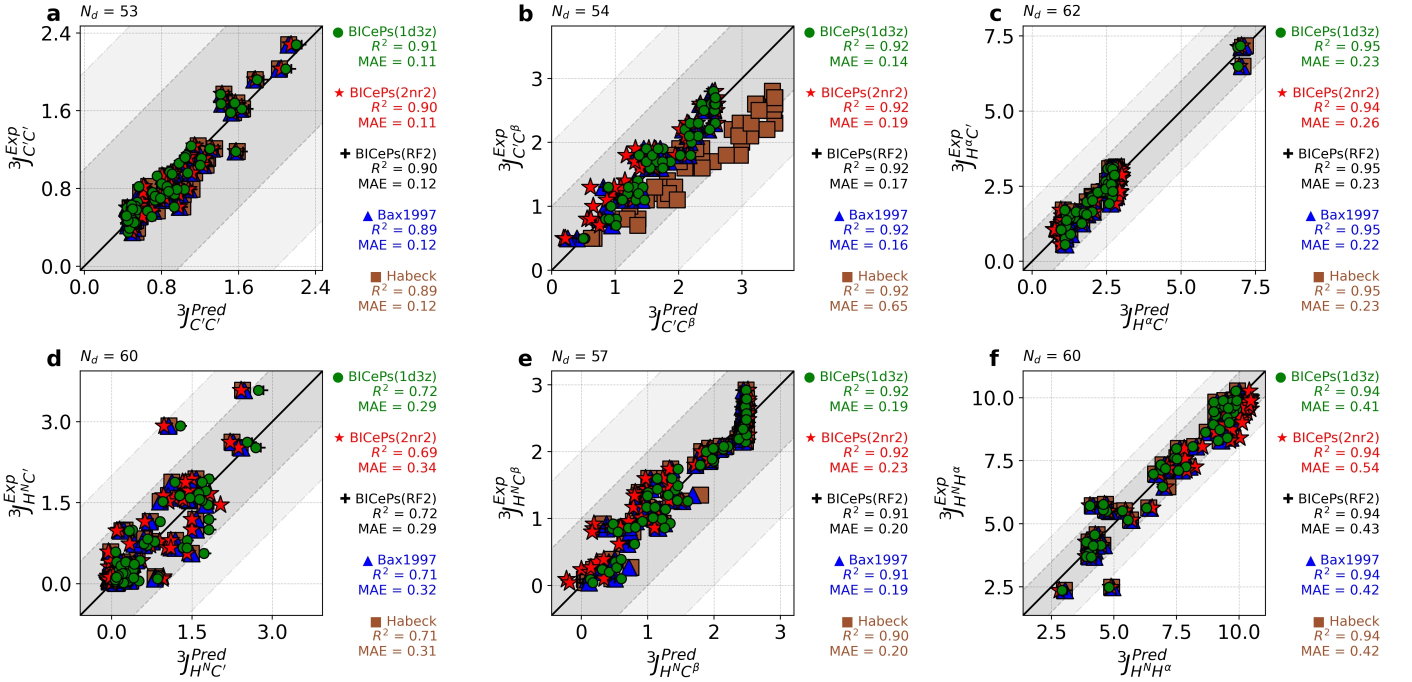

Figure S13. Validation of refined Karplus coefficients using BICePs on the 1D3Z structural ensemble show similar results to Bax1997 and minor improvements over Habeck2005 for scalar coupling predictions. Here, we compare models for predicting six sets of scalar coupling constants. Each panel shows strong correlations and relatively low error.

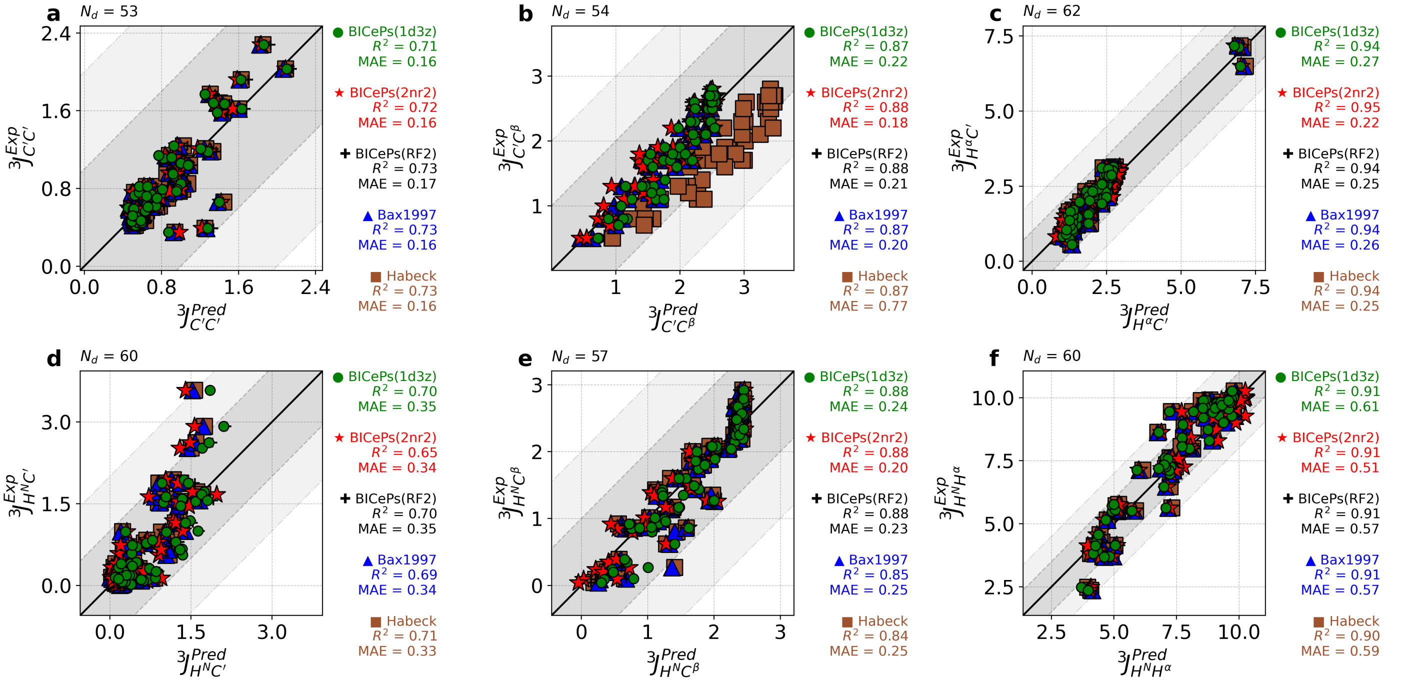

Figure S14. Validation of refined Karplus coefficients using BICePs on the 2NR2 structural ensemble show similar results to Bax1997 and minor improvements over Habeck2005 for scalar coupling predictions. Here, we compare various models for predicting six sets of scalar coupling constants. Each panel shows strong correlations between predictions and experiment.

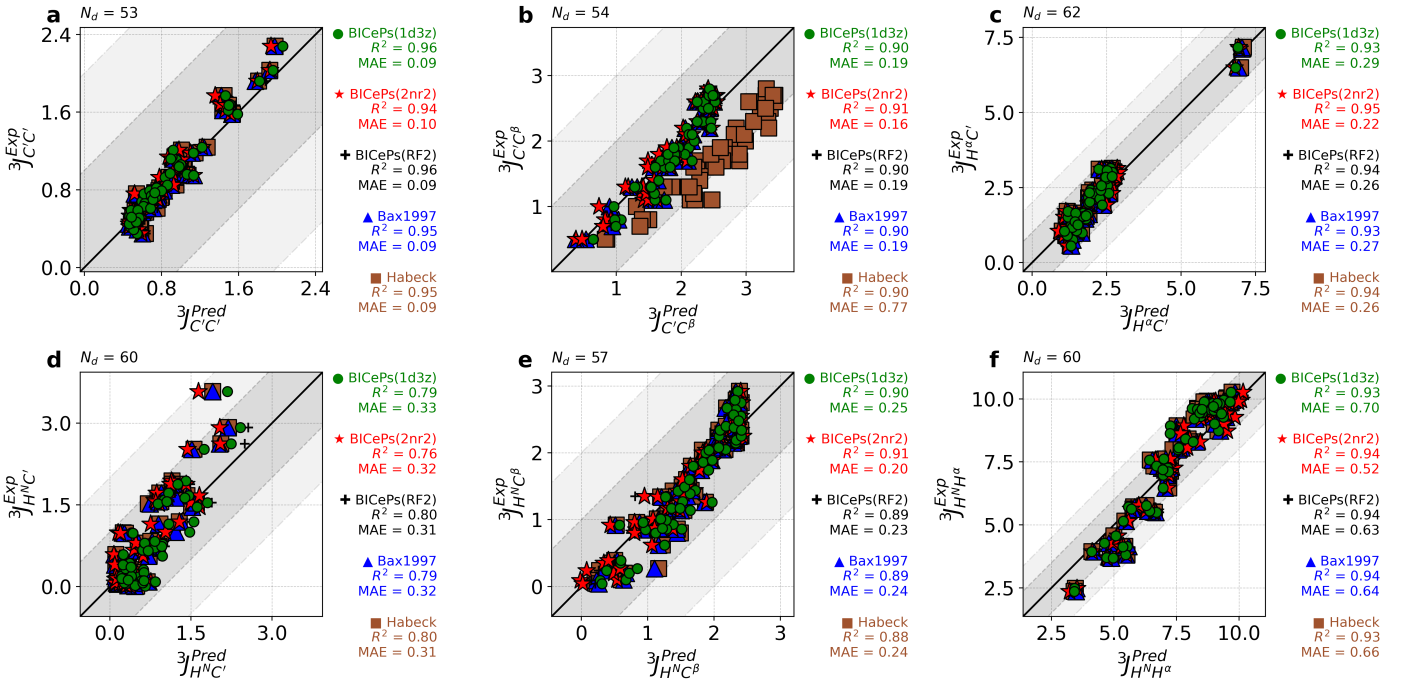

Figure S15. Validation of refined Karplus coefficients using BICePs on the CHARMM22* structural ensemble show similar results to Bax1997 and minor improvements over Habeck2005 for scalar coupling predictions. Here, we compare various sets parameters for predicting six sets of scalar coupling constants. Each panel shows strong correlations between predictions and experiment. On average, BICePs parameters derived from the 2NR2 ensemble give the lowest MAE between experiment and predictions, whereas Habeck2005 has the highest due to $^3J_{C'C^\beta}$.

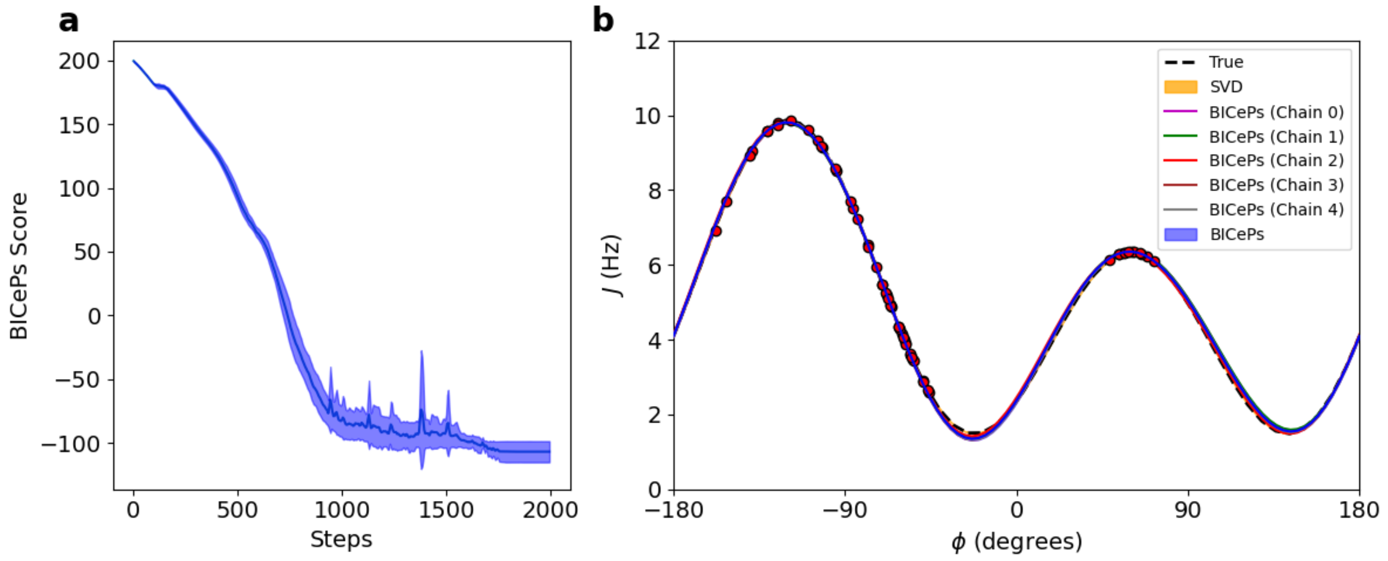

Figure S16. **BICePs trains NN parameters for J-coupling predictions using a toy model system.** (a) Minimization of the BICePs score during training of five independent neural networks (NNs). The solid blue line indicates the average BICePs score across the chains, with the shaded region representing the standard deviation. Convergence is evident by the plateau in score near 1500 training steps. (b) Karplus curves predicted by each of the five independently trained NNs. The mean prediction across all networks is shown in blue with uncertainty indicated by curve thickness (standard deviation across networks). The true curve (black dashed) corresponds to the parameters $A = 6.51, B = -1.76, C = 1.60$. Predictions obtained by singular value decomposition (SVD) are shown in orange, with extracted parameters $A = 6.47 \pm 0.002, B = -1.75 \pm 0.001, C = 1.62 \pm 0.001$. BICePs calculations were performed using a Good-Bad likelihood model and 32 replicas. No random or systematic noise was added to the synthetic data. Red dots correspond to the syntetic experimental J-coupling data points.

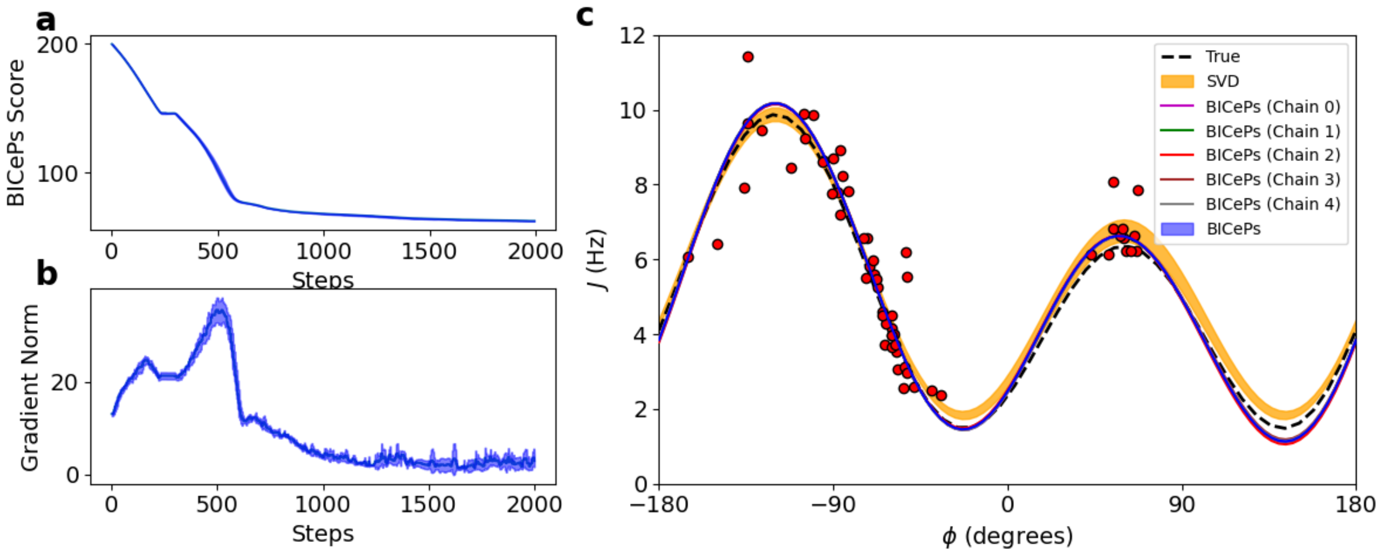

Figure S17. **BICePs trains NN parameters for J-coupling predictions using a toy model system in the presence of random and systematic error ($\sigma_{\mathbf{data}} = 0.79$ Hz).** (a) Minimization of the BICePs score during training of five independent neural networks (NNs). The solid blue line indicates the average BICePs score across the chains, with the shaded region representing the standard deviation. Convergence is evident by the plateau in score near 1000 training steps. (b) The average gradient norm across the five chains during training. (c) Karplus curves predicted by each of the five independently trained NNs. The mean prediction across all networks is shown in blue with uncertainty indicated by curve thickness (standard deviation across networks). The true curve (black dashed) corresponds to the parameters $A = 6.51, B = -1.76, C = 1.60$. Predictions obtained by singular value decomposition (SVD) are shown in orange, with extracted parameters $A = 6.39 \pm 0.13, B = -1.55 \pm 0.06, C = 1.94 \pm 0.10$. BICePs calculations were performed using a Good-Bad likelihood model and 32 replicas. Red dots correspond to the syntetic experimental J-coupling data points.
