# Supplementary material for: Automatic Forward Model Parameterization with Bayesian Inference of Conformational Populations
Source: ArXiv. 2024 May 28:arXiv:2405.18532v1. Preprint. [Version 1] (PMC11160882)
Supplement: Supplementary file 1 [file NIHPP2405.18532v1-supplement-1.pdf]

Appendix A: Variational minimization of the BICePs score to find optimal parameters.

It is likely that the second derivative w.r.t FM parameters would be useful for second-order optimization methods and uncertainty quantification. For the sake of simplicity, we refrain from writing out the second derivative for the Good-Bad model here. However, when $\varphi = 1$ the second partial derivatives of the BICePs energy with respect to parameters θ_i and θ_k are:

$$\frac{\partial^2 u}{\partial\theta_i\partial\theta_k} = N\left[\sum_{j=1}^{N_d} -\frac{\partial^2 g_j(X,\theta)}{\partial\theta_i\partial\theta_k}\frac{(d_j - g_j(\mathbf{X},\theta))}{\sigma_j^2} + \frac{\partial g_j(X,\theta)}{\partial\theta_i}\cdot\frac{\partial g_j(X,\theta)}{\partial\theta_k}\frac{1}{\sigma_j{}^2}\right]. \tag{A1}$$

When $i = k$, the second partial derivative of the energy is just:

$$\frac{\partial^2 u}{\partial\theta^2} = N\left[\sum_{j=1}^{N_d}\left(-\frac{\partial^2 g_j(X,\theta)}{\partial\theta^2}\frac{(d_j - g_j(\mathbf{X},\theta))}{\sigma_j^2} + \left(\frac{\partial g_j(X,\theta)}{\partial\theta}\right)^2\frac{1}{\sigma_j{}^2}\right)\right]. \tag{A2}$$

The second partial derivatives of the BICePs score with respect to parameters θ_i and θ_k are:

$$\frac{\partial^2 f(\theta)}{\partial\theta_i\partial\theta_k} = \left\langle\frac{\partial^2 u}{\partial\theta_i\partial\theta_k}\right\rangle - \left(\left\langle\frac{\partial u}{\partial\theta_i}\cdot\frac{\partial u}{\partial\theta_k}\right\rangle - \left\langle\frac{\partial u}{\partial\theta_i}\cdot\frac{\partial u}{\partial\theta_k}\right\rangle'\right), \tag{A3}$$

where the notation $\langle\cdot\rangle'$ denotes the ensemble average with respect to $\left(\frac{1}{Z}\exp(-u)\right)^2$. The first term on the right is the ensemble-averaged second derivative of the energy function u with respect parameters θ_i and θ_k is equation A2 (when $\varphi = 1$).

When $i = k$, the second partial derivative of the BICePs score reduces to the difference between the ensemble-averaged second derivative of the energy u and the variance of its first partial derivative:

$$\frac{\partial^2 f}{\partial\theta^2} = \left\langle\frac{\partial^2 u}{\partial\theta^2}\right\rangle - \left(\left\langle\left(\frac{\partial u}{\partial\theta}\right)^2\right\rangle - \left\langle\frac{\partial u}{\partial\theta}\right\rangle^2\right) \tag{A4}$$

The calculation is performed using the MBAR free energy estimator for the BICePs score and it's derivatives, by sampling at several intermediates $\xi = 0 \rightarrow 1$, which enables accurate estimates of all quantities.

In the case of convex landscapes, one can compute reported uncertainties in the set of optimized FM parameters θ by estimation of the covariance through inversion of the Hessian (the matrix of second partial derivatives of the BICePs score). Conceptually, this means that the estimated uncertainties in the best-fit values represent the widths of the basins on the BICePs score landscape. For non-convex problems, this can sometimes lead to an under-estimation of uncertainty depending upon the curvature of the basins of the local minima in the BICePs score landscape.

Appendix B: Supplemental Information

1. Methods

Structural ensembles of ubiquitin

1D3Z Ubiquitin NMR structural ensemble that consists of 10 conformations[26].

2NR2 Next, we use an ensemble (144 conformations) from Richter et al. (PDB: 2NR2), where the refinement was performed using the MUMO (minimal under-restraining minimal over-restraining) method[27]. In this approach, simulations started from the X-ray crystal pose[30]. NOEs and S^2 Lipari–Szabo order parameters were used as NMR structural restraints during a replica-averaged restrained simulation with TIP3P solvent and CHARM22 force field. The augmented potential energy function given as: $E_{total} = E_{CHARMM22} + E_{restraints}$. Scalar couplings were not used during the refinement, but were only used as a validation metric.

RosettaFold2 (RF2) Using the Colabfold notebook[35] with default parameters, 25 conformations were predicted using RosettaFold2[28].

*CHARMM22** An ensemble of conformationa states was derived from a Markov State Model (MSMs) were constructed from a 1-ms simulation of ubiquitin's native state at 300 K from Piana et al.[29]. The PyEMMA Python package[36] was used to determine appropriate backbone featurizations using the Variational Approach for Markov Processes (VAMP) scoring function VAMP-2[37]. Based on the obtained scores, inverse distances were selected as features, while torsions were excluded due to lower VAMP-2 scores and minimal contribution when paired with inverse distances. When comparing distances and inverse distances, the similarity of the average scores and relative standard deviations show that both featurizations are adequate, and inverse distances was selected purely on the higher average scores. Time-lagged independent component analysis (tICA) followed by k-means clustering was used to discretize simulations into a structural ensemble of 500 conformational states for MSM construction[38,39]. For the BICePs calculation, each of the 500 states were given equal statistical weight to enforce the uniform prior $p(X)$.

Details of parameters used in posterior sampling

The range of sampled uncertainty parameters σ on a grid of logarithmically-spaced values between 0.001 to 100, to enforce the Jeffrey's prior. Each grid value in the list was a factor of 1.02 larger than the next: [1.00e-03, 1.02e-03, 1.04e-03, 1.06e-03, ... 9.72e+01, 9.92e+01], resulting in a list of 582 values. For the Good-Bad model, sampling of the extra nuisance parameter, φ took place on a grid from 1 to 100 with 1000 equally-spaced points.

Details of ξ optimization

Optimization was performed for a maximum of 2M steps with a tolerance of 1e-7 and $\alpha = 1e-5$. With increasing amounts of data restraint energy, the optimization problem becomes more complicated and more iterations are required to converge. In the case of insuffficent iterations, the ξ-optimization might return negative ξ-values, which is incorrect and not physical. Initially, we start with 11 ξ-values {1.0, 0.9, 0.8,...,0.0} and with shift to lower values of ξ after optimization e.g., {1.0, 0.7, 0.56, 0.45, 0.36, 0.28, 0.2, 0.14, 0.08, 0.04, 0.0}.

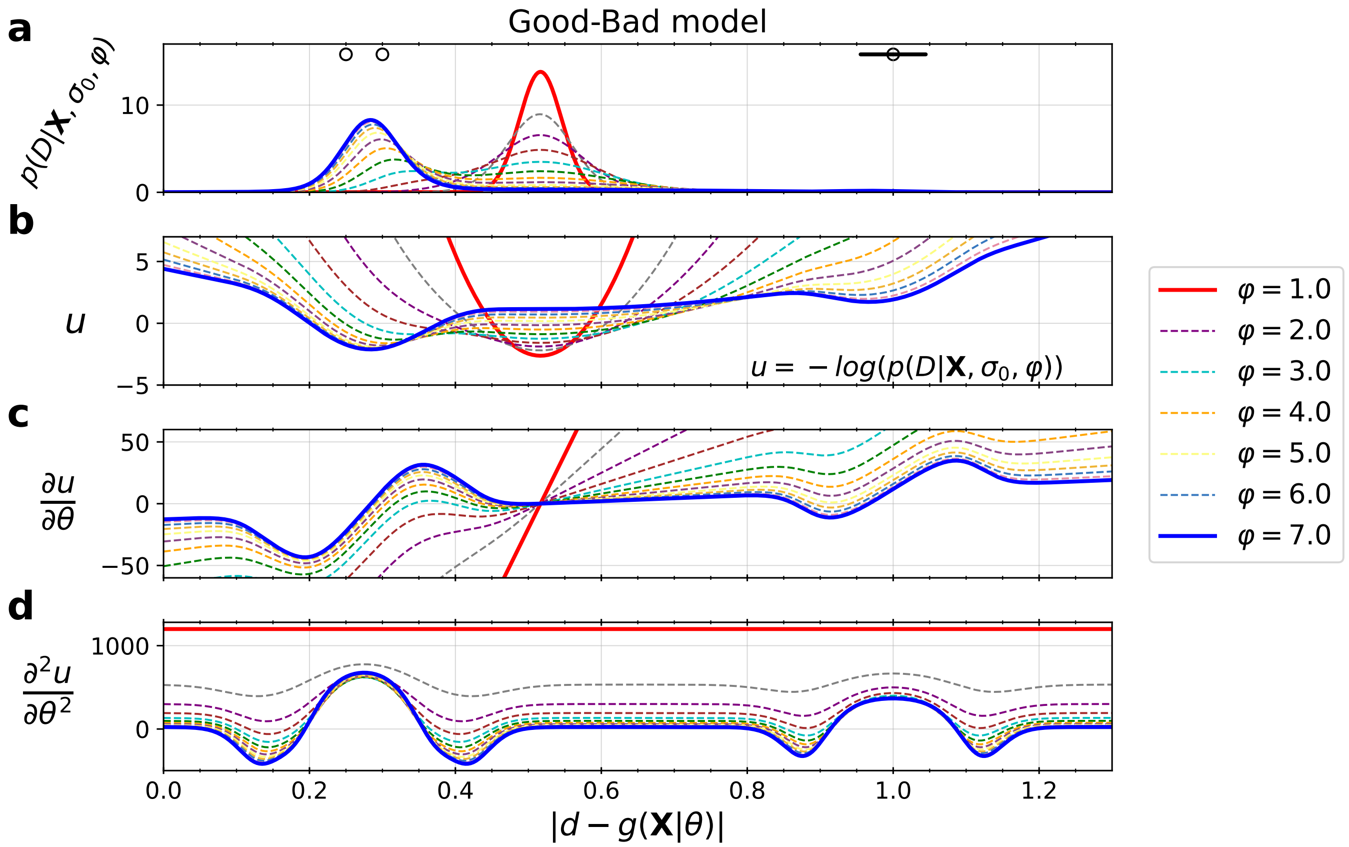

Figure S1. The Good-Bad model properly detects outliers. The probability density function (a) computed as $p(D|\mathbf{X},\sigma_0,\varphi)=\prod_j^{N_j} p(d_j|\mathbf{X},\sigma_0,\varphi)$ and energy landscape (b) of the marginal likelihood for the Good-Bad model with respect to the replica-averaged forward model data $f(\mathbf{X})$ using multiple data points. Shown here, are three data points, two good data points $\{0.25, 0.3\}$ and one outlier $\{1.0\}$. The Good-Bad model ($\varphi = 7.0$) is centered about the mean of the two good data points, demonstrating that this model can distinguish the good and bad data. The standard Gaussian likelihood ($\varphi = 1$) is centered about the mean of all three data points. The colored curves are different values of nuisance parameter φ. Subplots (a) and (b) show how the Good-Bad model when $\varphi = 1$ is equivalent to the Gaussian likelihood and harmonic potential energy function. Subplots (c) and (d) are the first and second derivatives of the potential energy curves shown in subplot (b).

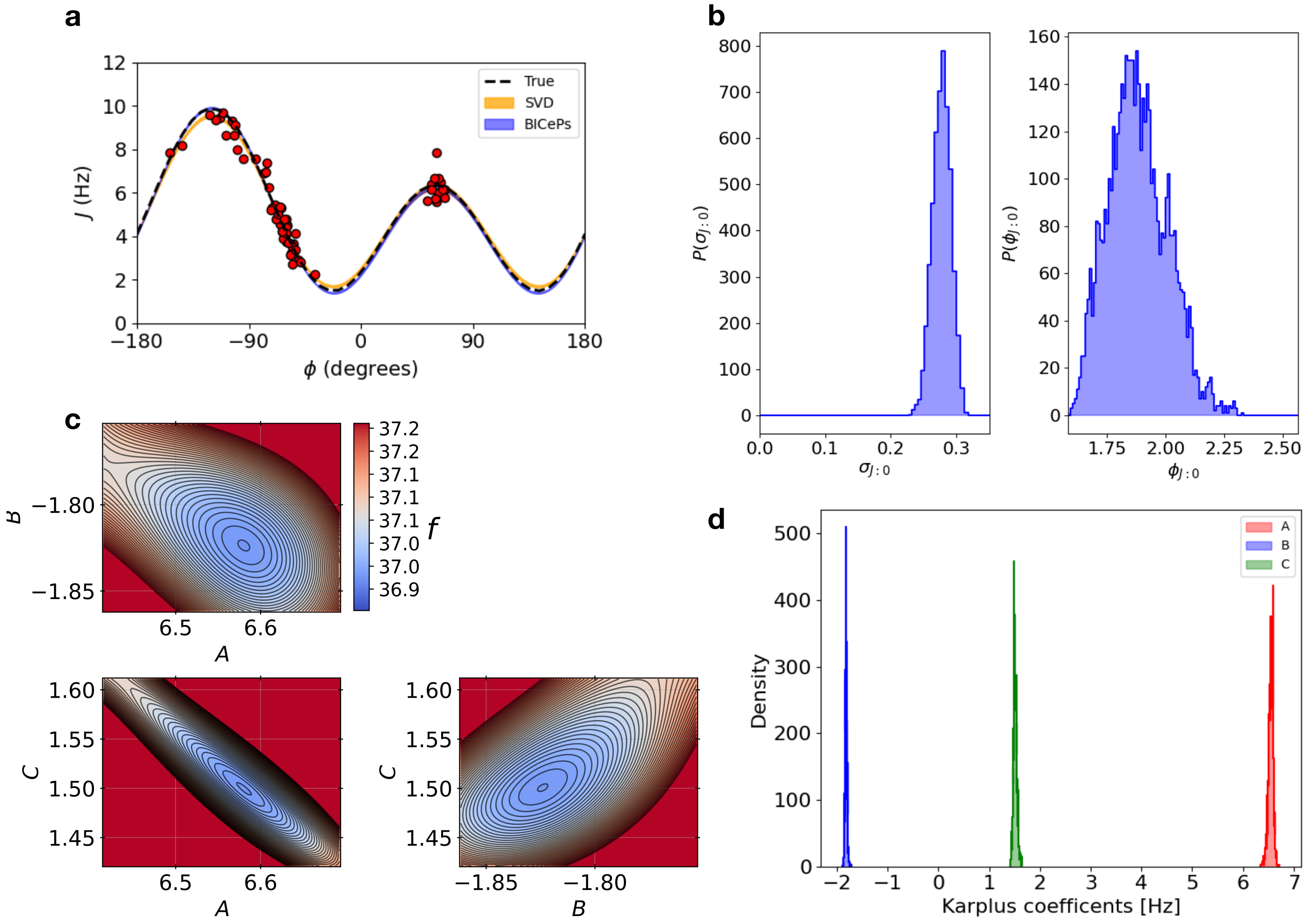

Figure S2. **BICePs predicted forward model parameters in the presence of random and systematic error** ($\sigma_{data} = 0.471$) **for a toy model system.** (a) Karplus curves predicted for SVD (orange) and BICePs (blue), where the "true" (black dashed line) parameters were set to be $\{A = 6.51, B = -1.76, C = 1.6\}$. The extracted parameters from the SVD fitting were found to be $\{A = 6.11 \pm 0.06, B = -1.63 \pm 0.04, C = 1.80 \pm 0.04\}$ and the BICePs was $\{A = 6.6 \pm 0.037, B = -1.8 \pm 0.016, C = 1.5 \pm 0.027\}$, averaged over three independent chains. The uncertainty is represented by the thickness of the curves. For the BICePs calculation, we used the Good-Bad likelihood model with 32 replicas and burned for 20k steps, followed by 50k steps of additional sampling. (b) The marginal posterior distribution of uncertainty $p(\sigma_J)$. The maximum a posteriori was determined to be $\hat{\sigma}_J = 0.272$, and the *a posteriori* variance scaling parameter $\hat{\phi}_J = 1.98$. (c) Landscapes of the BICePs score, f for pairs of Karplus coefficients. (d) The marginal posterior distribution of Karplus coefficients.

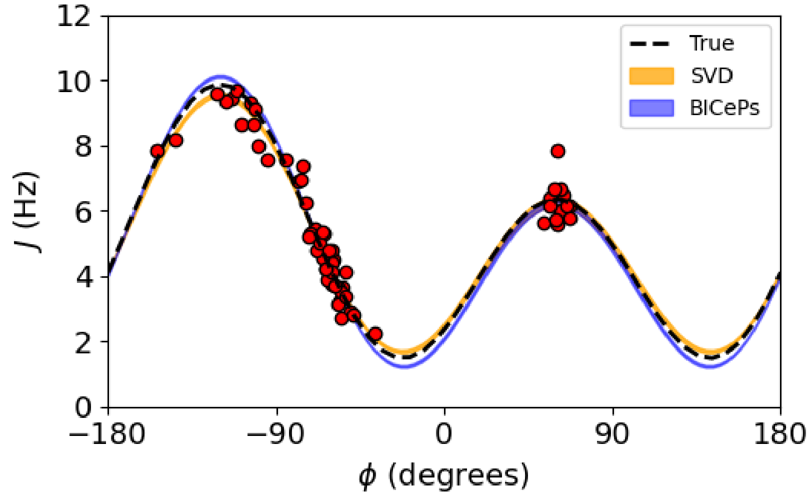

Figure S3. **The Student's model gives similar performance to the Good-Bad model.** Karplus coefficients predicted using the Student's likeliood model ($\{A = 6.8 \pm 0.033, B = -1.9 \pm 0.025, C = 1.4 \pm 0.033\}$) are compared against SVD when faced with random and systematic error ($\sigma_{data} = 0.471$). The "True" parameters were set to be $\{A = 6.51, B = -1.76, C = 1.6\}$, the same as Figure S2.

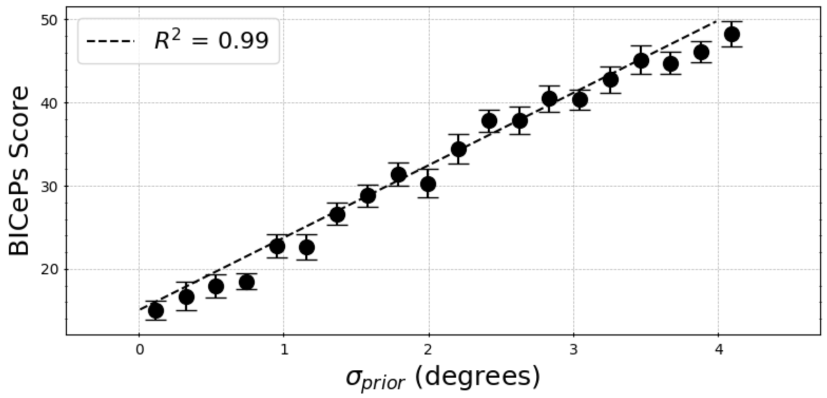

Figure S4. **The BICePs score is a measure of structural ensemble quality.** Using the same toy model system described in the main text. We vary the quality of the prior structural ensemble (σ_{prior}) by perturbing the "true" ϕ angles of the structural ensemble. In these experiments, we induced over 1,000 random perturbations to the prior structural ensemble, and calculated BICePs scores for each. Error bars represent the standard deviation from the mean. The top panel shows the relationship between the BICePs score and the amount of error added to the structural ensemble. Each data point is an average across of 100 BICePs calculations. In these calculations, we used the Good-Bad likelihood model with 32 replicas.

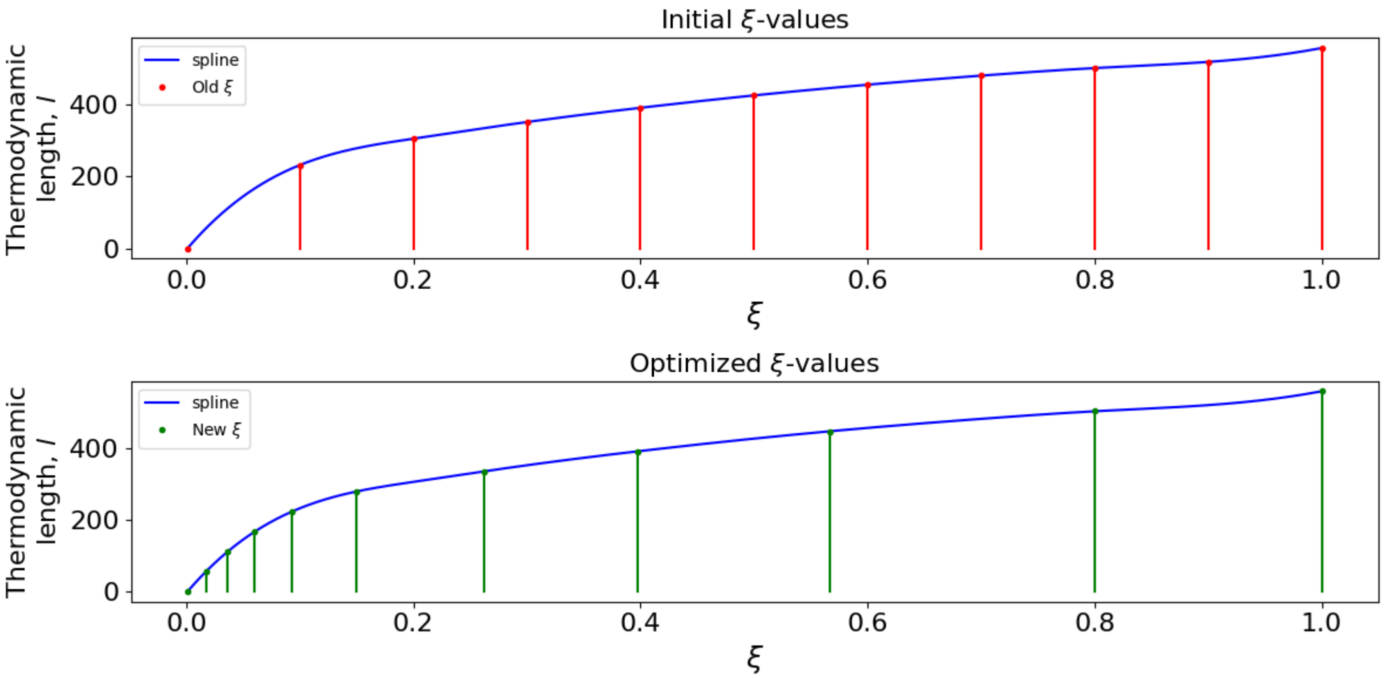

Figure S5. **Optimization of ξ-values to enhance free energy estimation**. The y-axis is thermodyamic length, $|\ell(\xi_{n+1}) - \ell(\xi_n)|$ for each pair of intermediates, derived from the variance in distributions $p(\Delta u_{n,n+1})$. The top panel shows ξ_i values before optimization. The bottom panel shows the optimized ξ_i^* values. Optimized ξ-values have shifted to smaller values to make up for the lack of overlap between these adjacent thermodynamic ensembles.

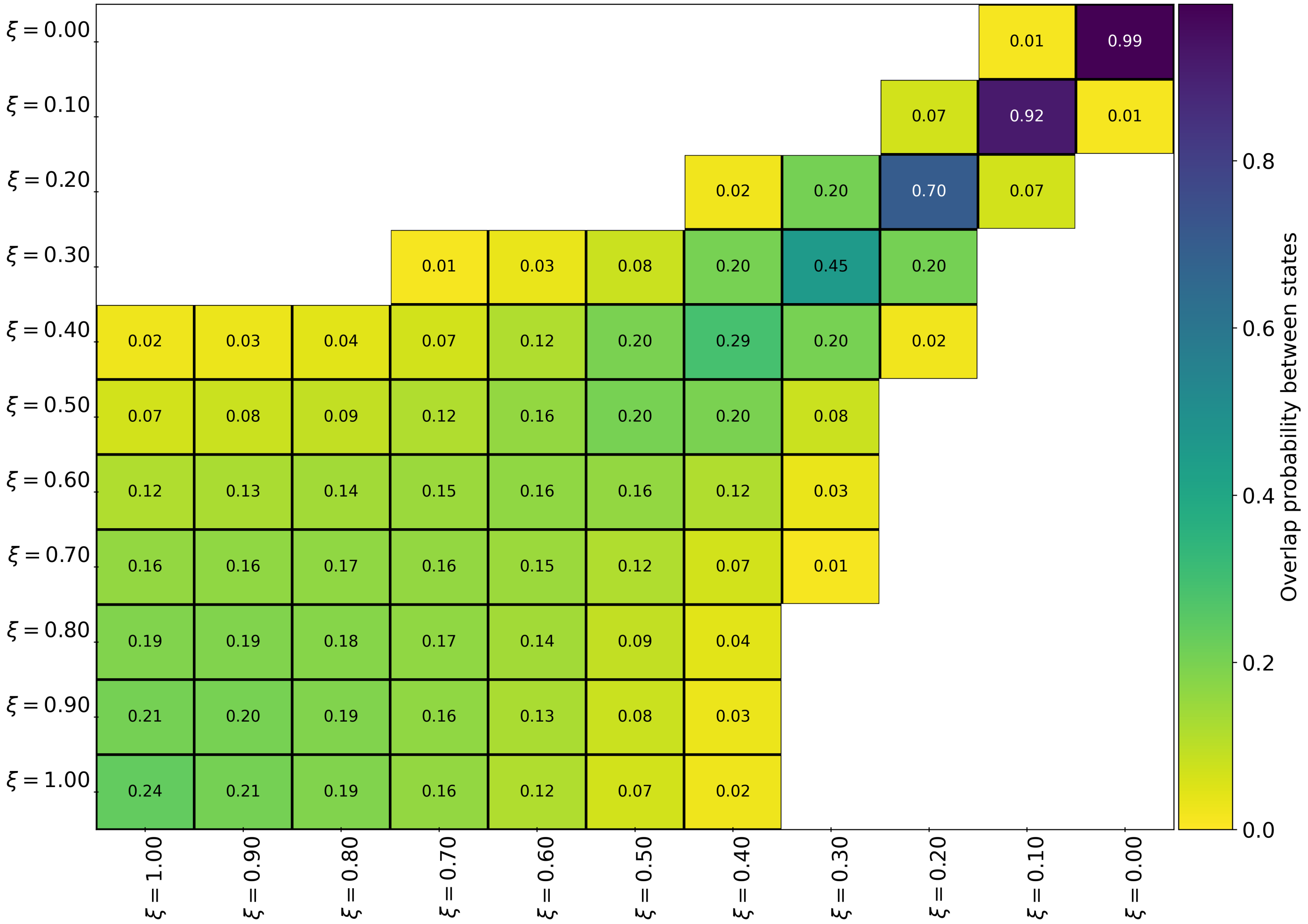

Figure S6. The overlap matrix O_{ij} between thermodynamic states i and j for intermediates along the ($\xi = 0 \rightarrow 1$) transformation, **before** optimization of ξ-values. The overlap matrix (Klimovich et al 2015) is a Monte Carlo estimate of the average probability of samples in thermodynamic state j being observed in thermodynamic state i: $O_{ij} = \left\langle \frac{N_i p_i(\mathbf{x})}{\sum_{k=1}^{K} N_k p_k(\mathbf{x})} \right\rangle_j$, where N_i are the number of samples from thermodynamic state i and $p_i(\mathbf{x})$ are posterior probabilities. Significant off-diagonal overlap provides evidence of reliable free energy estimates.

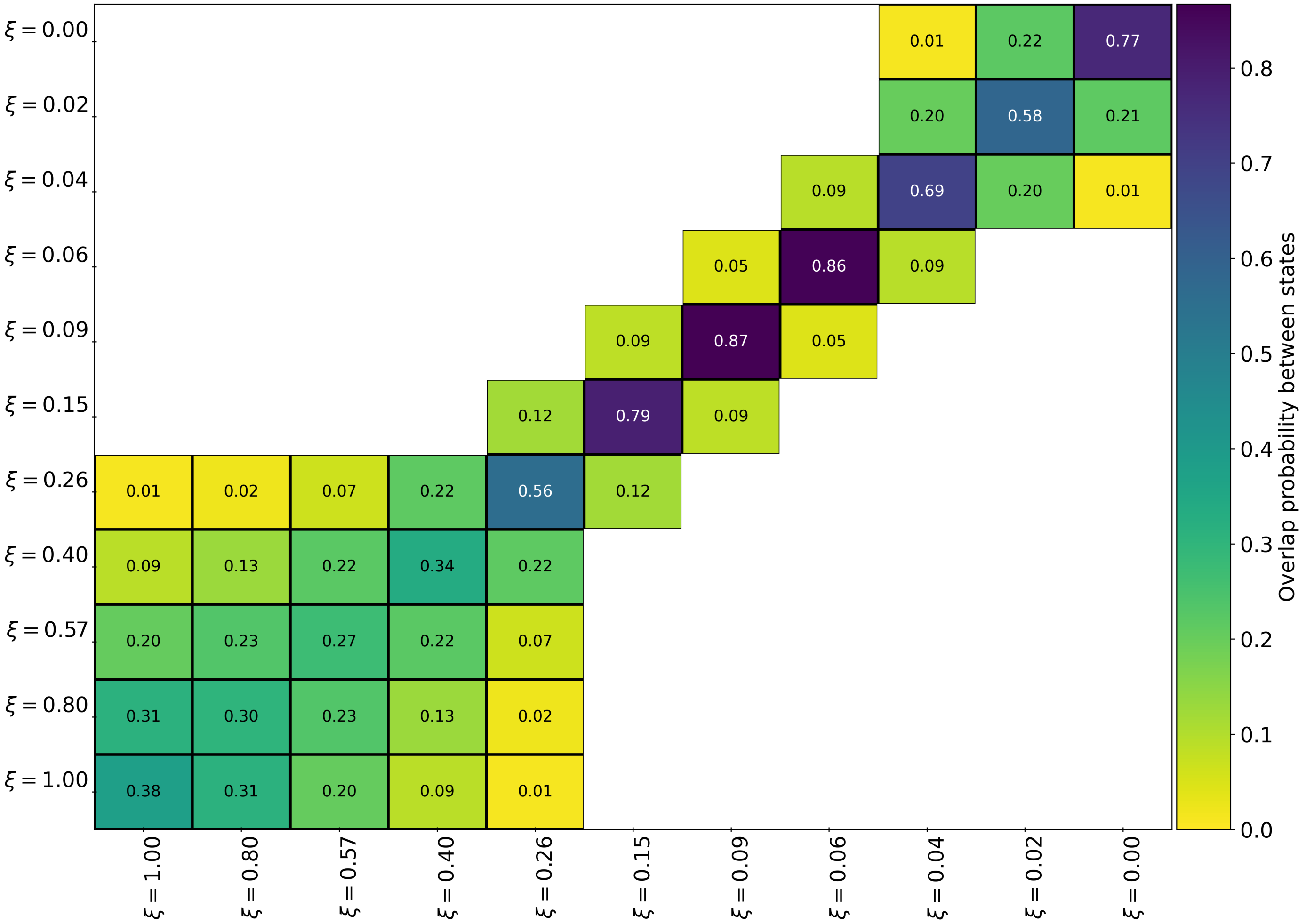

Figure S7. The overlap matrix O_{ij} between thermodynamic states i and j for intermediates along the ($\xi = 0 \rightarrow 1$) transformation, **after** optimization of ξ-values. Stronger overlap is clearly demonstrated by comparison of post- and pre-optimization (Figure S6).

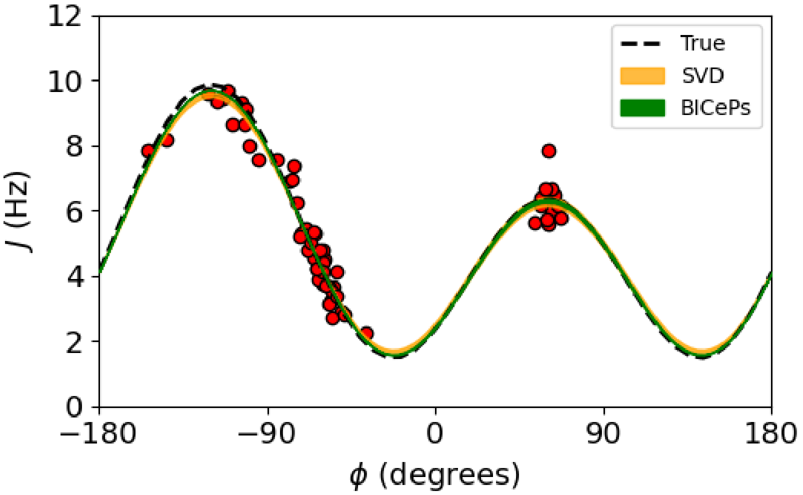

Figure S8. **Variational minimization of the BICePs score can be used to optimize forward model parameters.** By variational minimzation of the BICePs score, f optimization traces converge to the "true" parameters. In these calculations, ξ-values were optimized prior to running the parameter refinement. We used the Good-Bad model with 4 replicas to minimize computational cost. Karplus coefficients predicted using the Good-Bad likeliood model ($\{6.31 \pm 0.02, -1.69 \pm 0.03, 1.69 \pm 0.01\}$) are compared against SVD when faced with random and systematic error ($\sigma_{data} = 0.471$). The "True" parameters were set to be $\{A = 6.51, B = -1.76, C = 1.6\}$, the same as Figure S2.

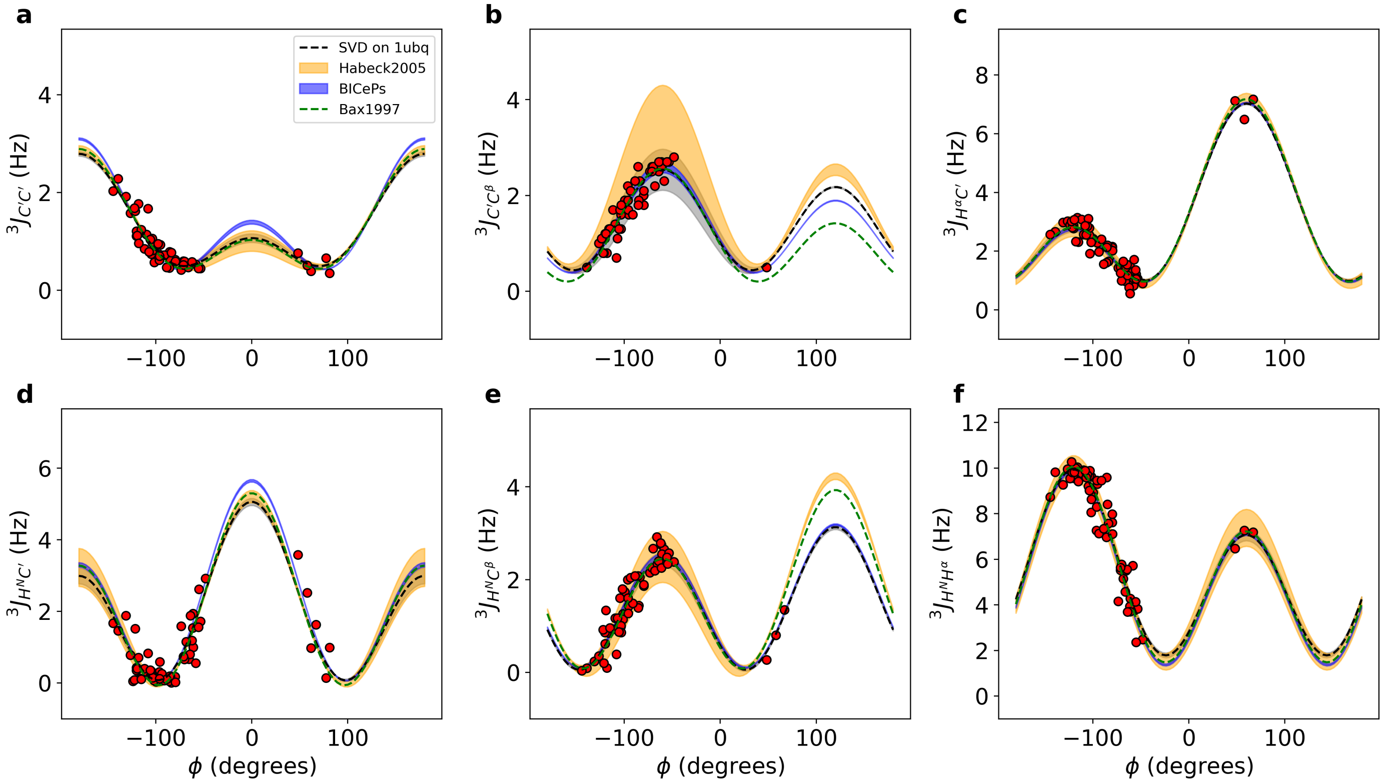

Figure S9. Karplus curves with BICePs refined Karplus coefficients using the 1d3z ensemble for (a-f) $^3J_{C'C'}$, $^3J_{C'C^\beta}$, $^3J_{H^\alpha C'}$, $^3J_{H^N C'}$, $^3J_{H^N C^\beta}$, $^3J_{H^N H^\alpha}$. BICePs calculations were run using four chains with 32 replicas each, where we burned 50k steps, then sampled for another 50k MCMC steps. For comparison, SVD on 1ubq using experimental scalar coupling constants with ϕ-angles derived from the X-ray structure (black dashed line) and red dots correspond to the fitted data points. Additionally, parameterizations from Bax et al. 1997 (green), and parameterization from Habeck et al. 2005 (yellow) were overlaid for comparison. The thickness of the line corresponds to the uncertainty.

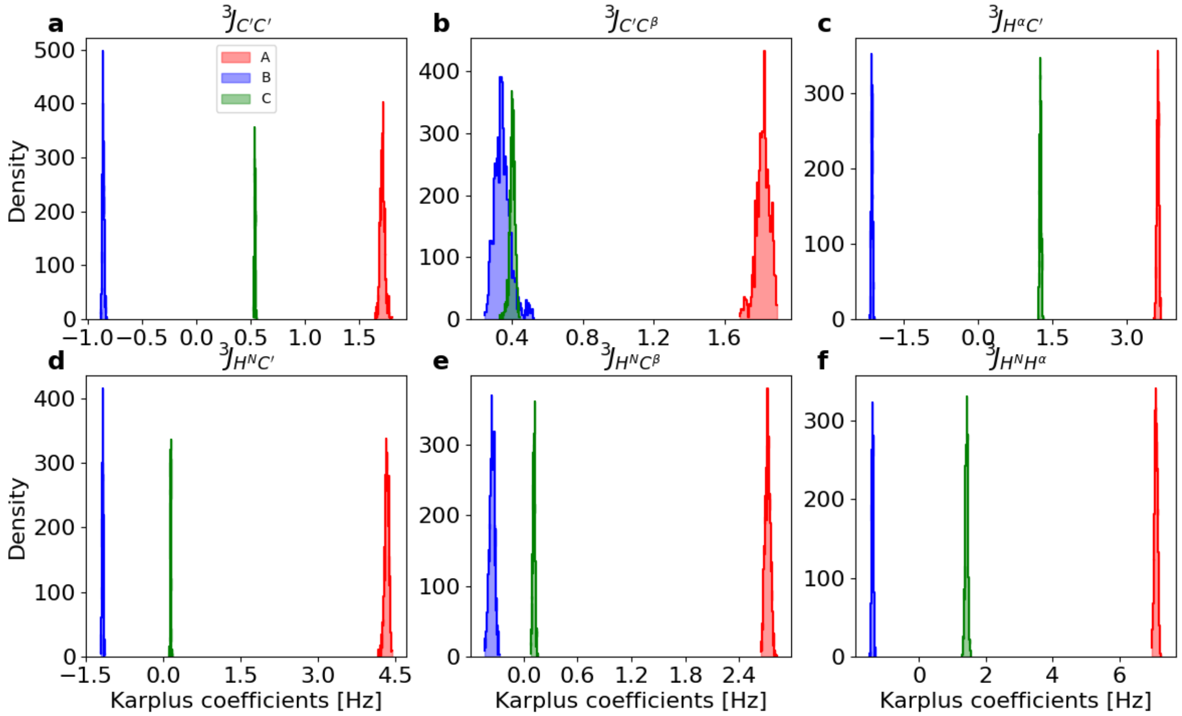

Figure S10. **Sampling the joint posterior distributions of six sets of Karplus coefficients using the Good-Bad model on the 1d3z ensemble.** BICePs calculations were run using four chains with 32 replicas each, where we burned 50k steps, then sampled for another 50k MCMC steps. These marginal posterior distributions shown here are from a randomly selected chain.

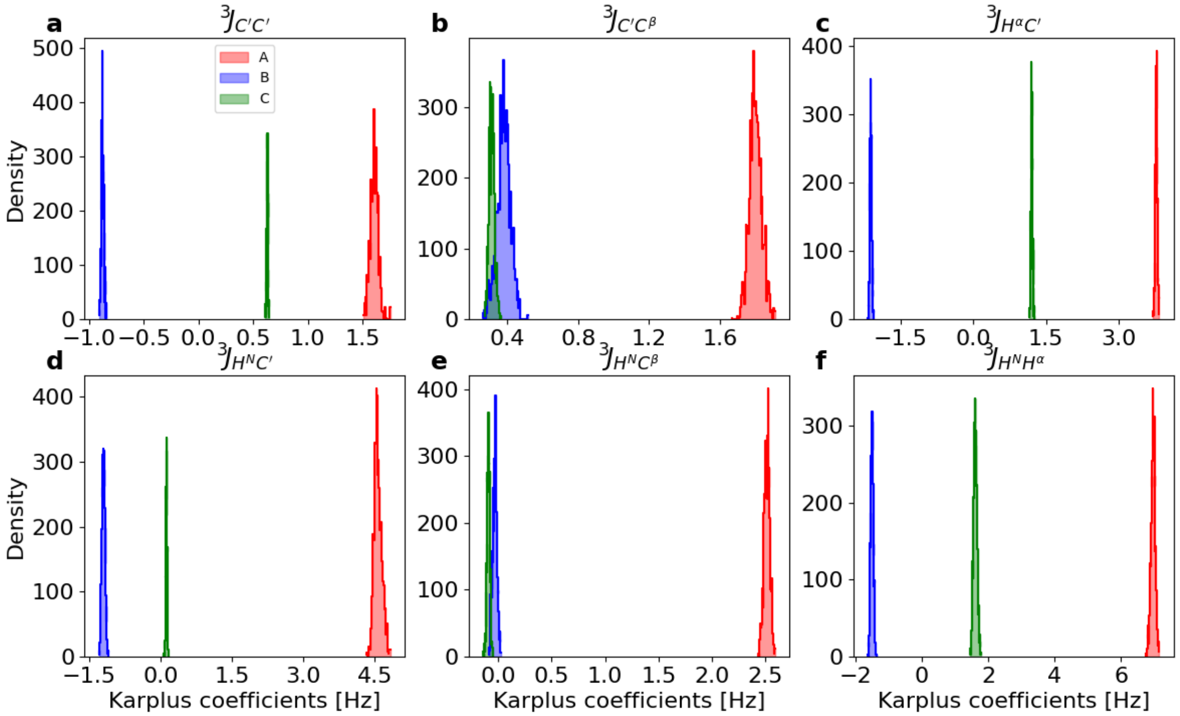

Figure S11. **Sampling the joint posterior distributions of six sets of Karplus coefficients using the Good-Bad model on the RosettaFold2 (RF2) ensemble.** BICePs calculations were run using four chains with 32 replicas each, where we burned 50k steps, then sampled for another 50k MCMC steps. Compare with Figure S10 to see similarities.

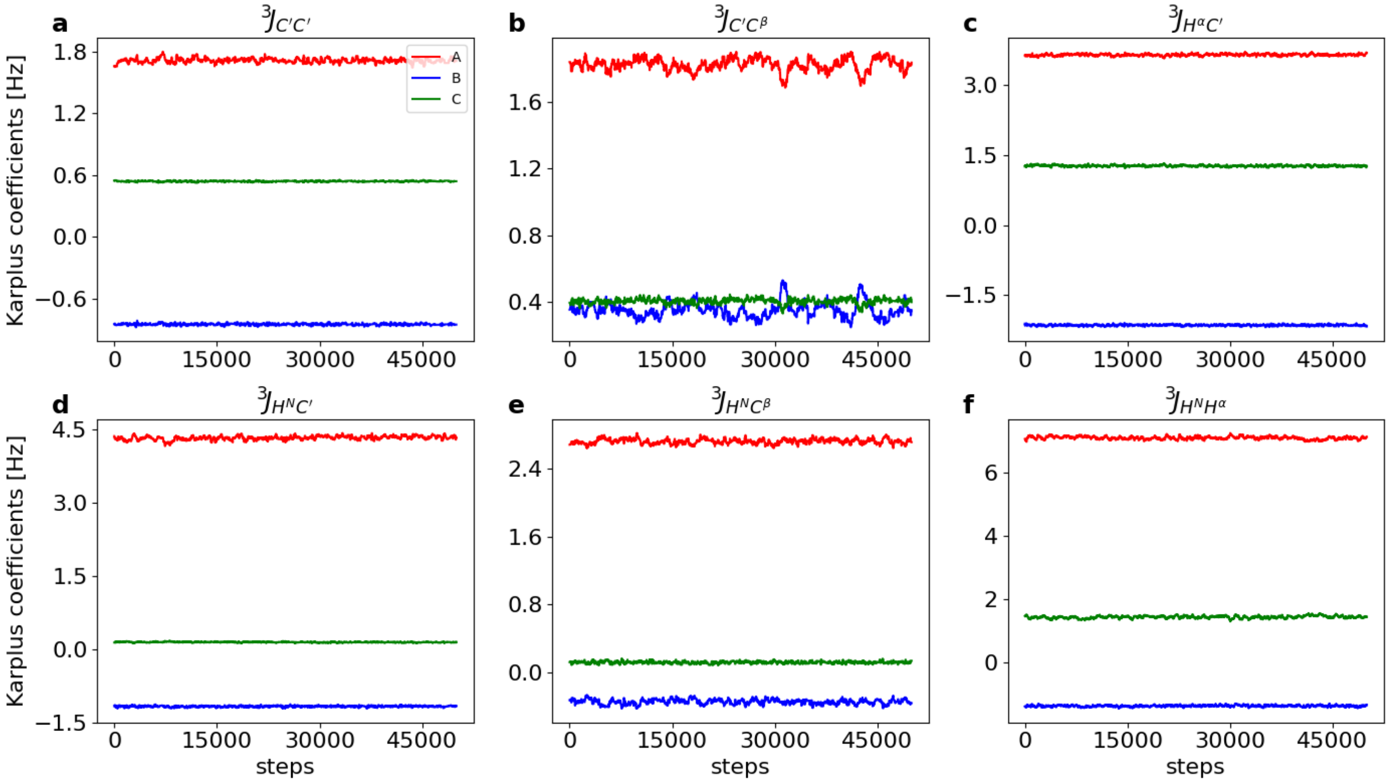

Figure S12. Traces of sampled Karplus coefficients for the 1d3z ensemble over 50k steps of MCMC, post-burn. BICePs calculations used the Good-Bad model with 32 replicas. Traces display low variance with no jumps, which demonstrates converged samples

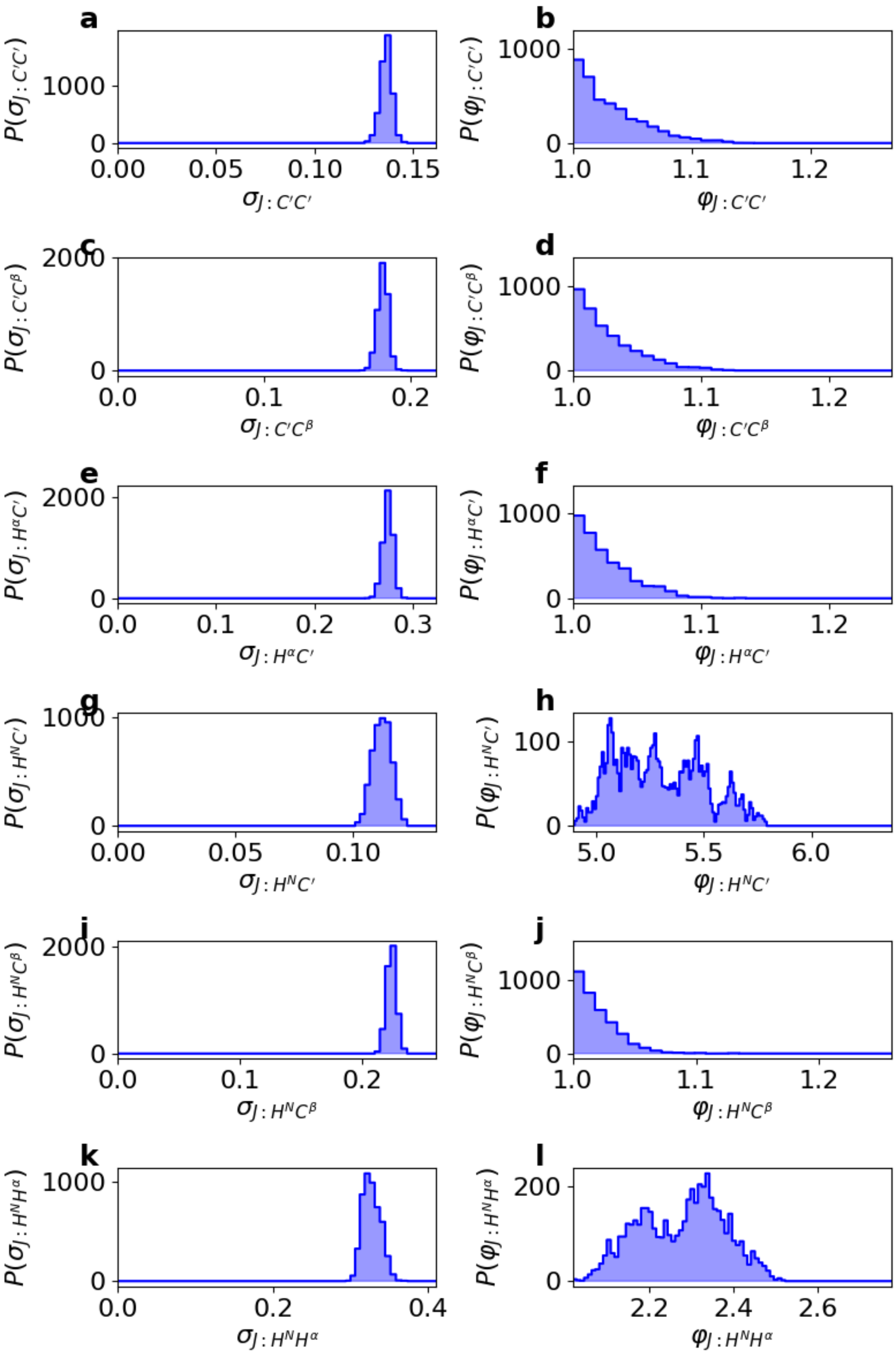

Figure S13. The marginal posterior distribution of σ_J, the uncertainty parameters for each set of J-coupling in the 1d3z ensemble. Densities are a result of posterior sampling of FM parameters during ensemble refinement using the Good-Bad model with 32 replicas. The marginal posterior distributions of the variance scaling parameter φ has a sampled mean slightly larger than 1.0 for particular sets of J-coupling, indicating that the functional form of the likelihood opted for long tails to account for a few outlier data points deviating from the mean.

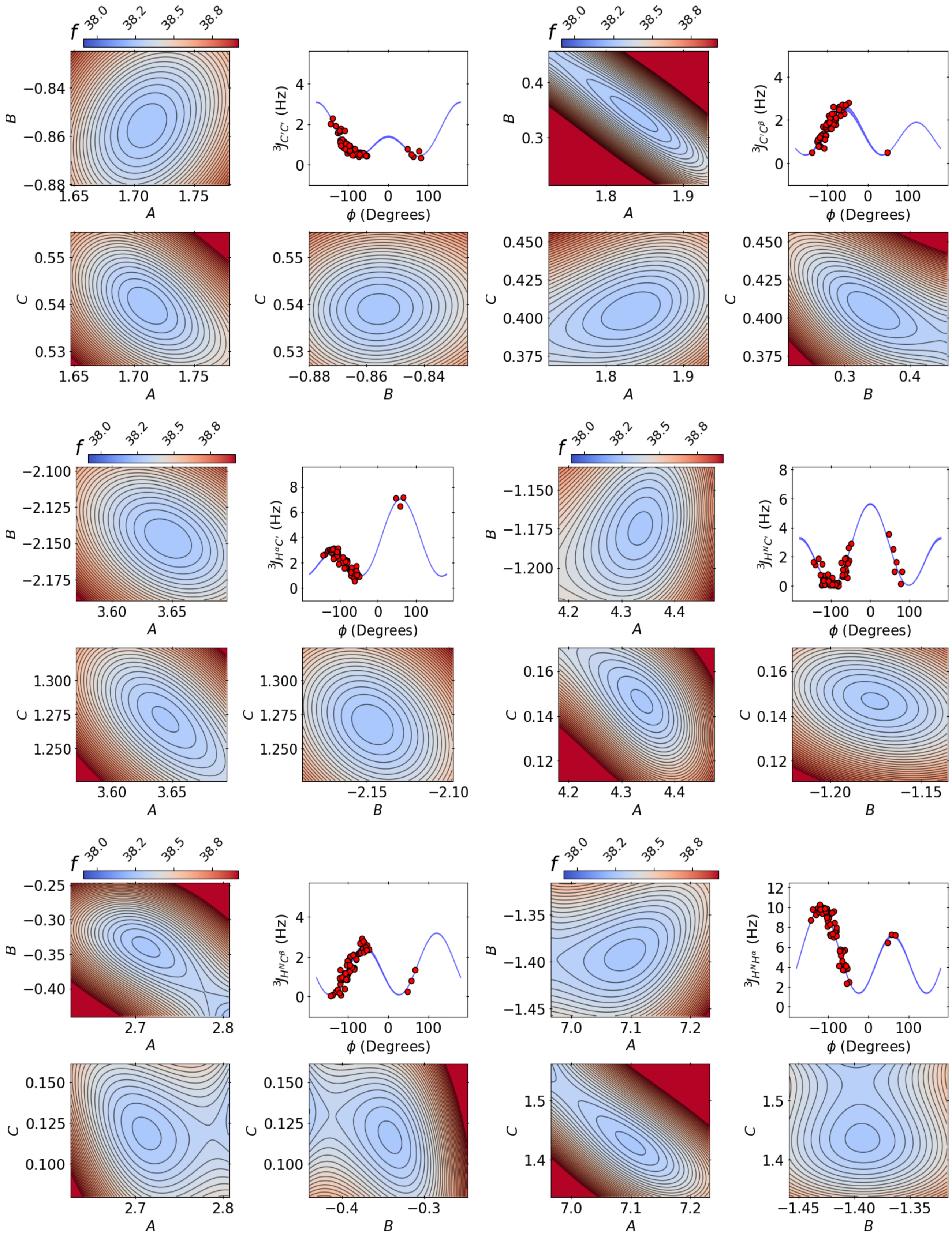

Figure S14. BICePs score landscapes of FM parameters on the 1d3z ensemble, unveiled during ensemble refinement. BICePs calcualtions used the Good-Bad model with 32 replicas. Each set of $\{A, B, C\}$ was included in the joint posterior of FM parameters.

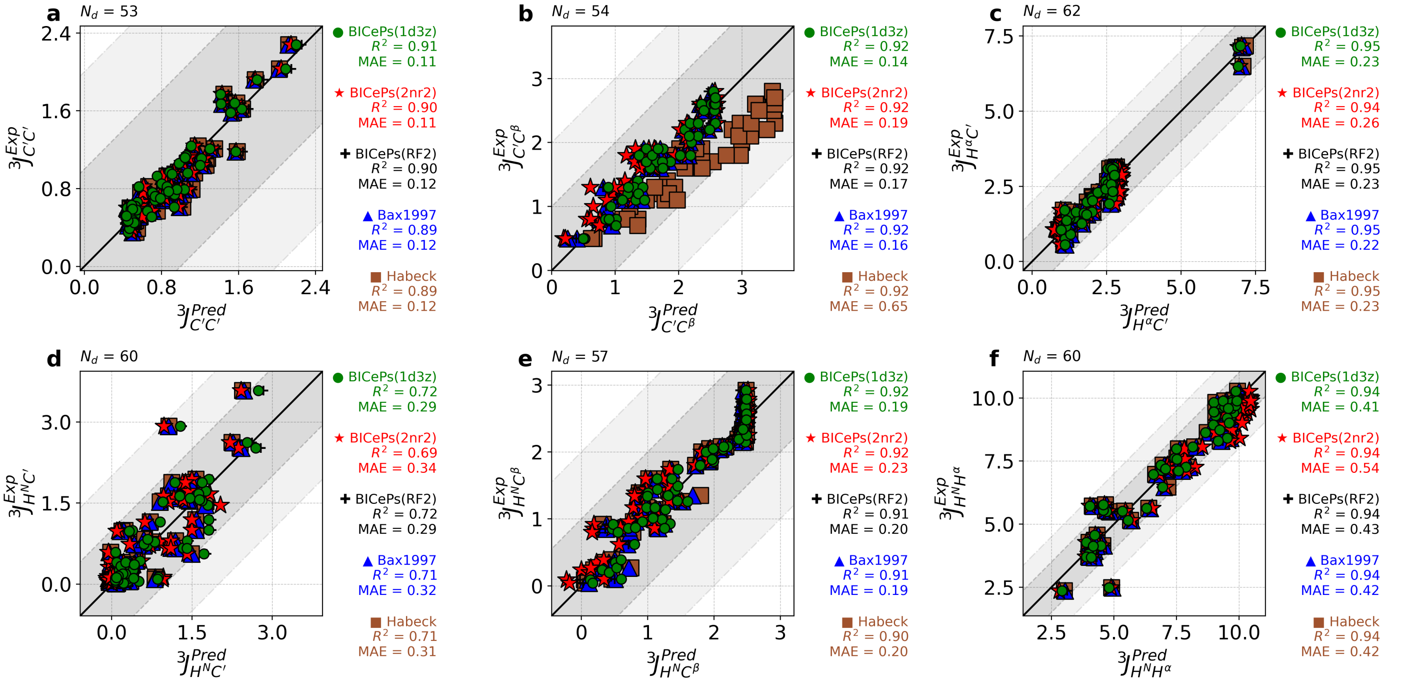

Figure S15. Validation of refined Karplus coefficients using BICePs on the 1D3Z structural ensemble show similar results to Bax1997 and minor improvements over Habeck2005 for scalar coupling predictions. Here, we compare models for predicting six sets of scalar coupling constants. Each panel shows strong correlations and relatively low error.

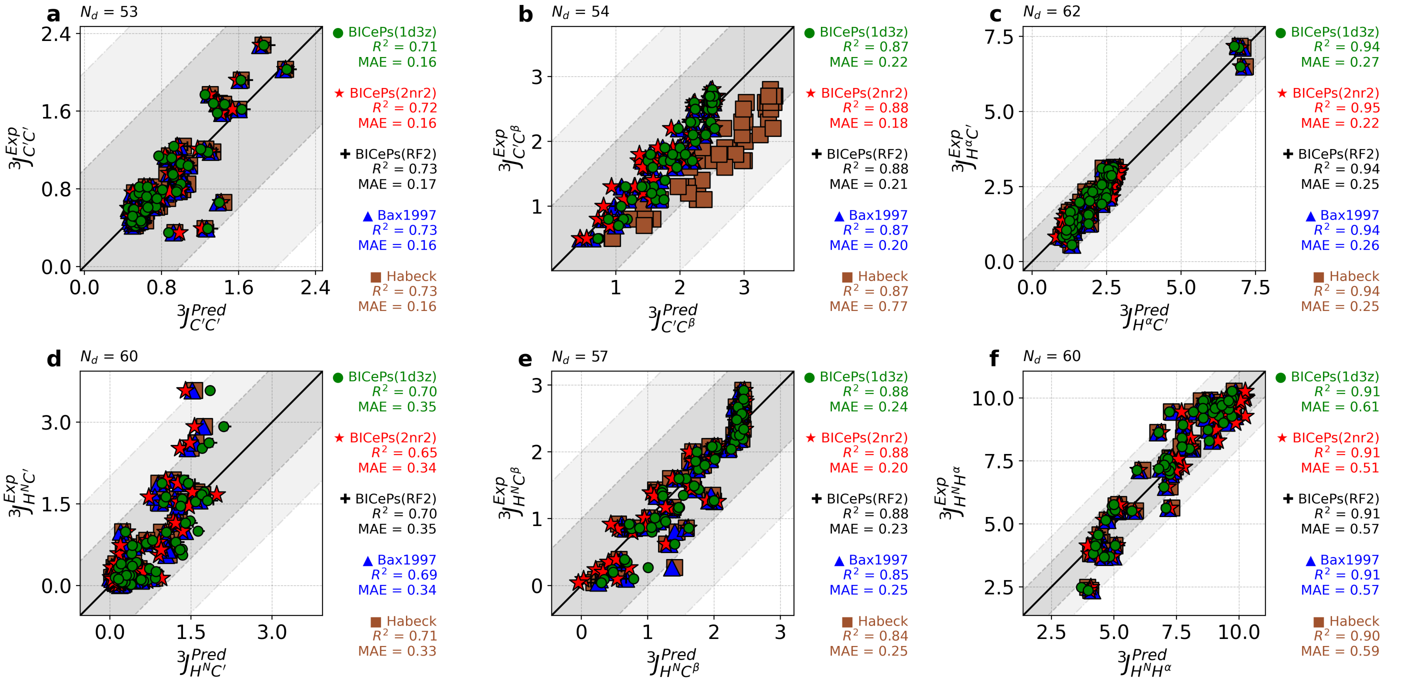

Figure S16. Validation of refined Karplus coefficients using BICePs on the 2NR2 structural ensemble show similar results to Bax1997 and minor improvements over Habeck2005 for scalar coupling predictions. Here, we compare various models for predicting six sets of scalar coupling constants. Each panel shows strong correlations between predictions and experiment.

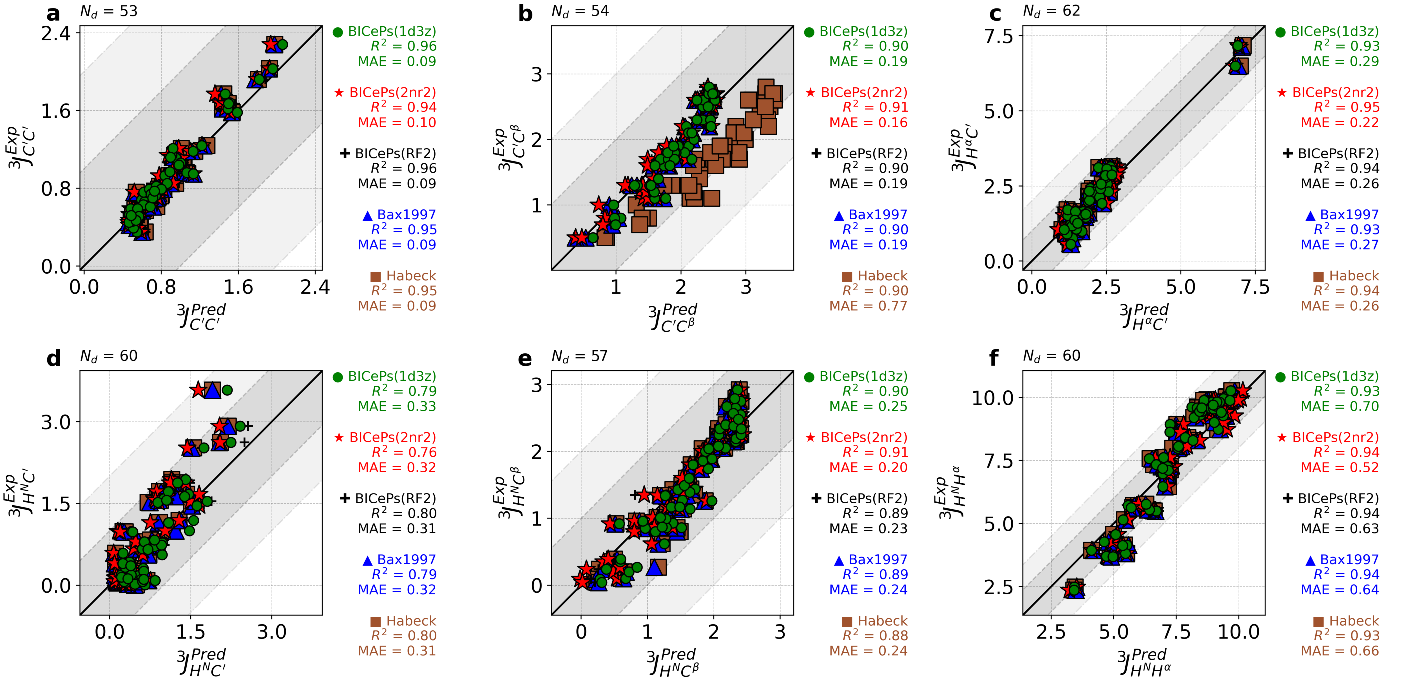

Figure S17. Validation of refined Karplus coefficients using BICePs on the CHARMM22* structural ensemble show similar results to Bax1997 and minor improvements over Habeck2005 for scalar coupling predictions. Here, we compare various sets parameters for predicting six sets of scalar coupling constants. Each panel shows strong correlations between predictions and experiment. On average, BICePs parameters derived from the 2NR2 ensemble give the lowest MAE between experiment and predictions, whereas Habeck2005 has the highest due to ${}^3J_{C'C\beta}$.
